# Impact of Media Guidelines on Suicide-Related Reporting Quality and Suicides: A Systematic Review and Meta-Analysis

**DOI:** 10.1027/0227-5910/a001049

**Published:** 2026-02-13

**Authors:** Simone Scotti Requena, Vikas Arya, Thomas Niederkrotenthaler, Mark Sinyor, Michiko Ueda, Matthew J. Spittal, Jane Pirkis

**Affiliations:** ^1^ Centre for Mental Health and Community Wellbeing, Melbourne School of Population and Global Health, University of Melbourne, Melbourne, VIC, Australia; ^2^ Unit Public Mental Health Research, Department of Social and Preventive Medicine, Center for Public Health, Medical University of Vienna, Vienna, Austria; ^3^ Department of Psychiatry, Sunnybrook Health Sciences Centre, Toronto, ON, Canada; ^4^ Department of Psychiatry, University of Toronto, Toronto, ON, Canada; ^5^ Department of Public Administration and International Affairs, Syracuse University, Syracuse, NY, USA; ^6^ Center for Policy Research, Maxwell School of Citizenship and Public Affairs, Syracuse, NY, USA

**Keywords:** media guidelines, suicide, systematic review, meta-analysis

## Abstract

**Abstract:** *Background:* Suicide-related media reporting can influence suicidal thoughts and behaviors. Stories describing suicide methods can increase suicides (Werther effect), whereas those highlighting coping with suicidal crises can prevent them (Papageno effect). *Aims:* To assess the impact of media guidelines for suicide reporting on reporting quality and suicides. *Method:* Following PRISMA guidelines, we searched PubMed, Scopus, Embase, PsycInfo, and Web of Science up to February 10, 2025, with an update on November 17, 2025. We included ecological pre–post studies assessing changes in reporting quality and time-series analyses assessing changes in suicides after guideline release. Random-effects meta-analyses estimated pooled risk ratios (RRs) for reporting quality and incidence rate ratios (IRRs) for suicides. *Results:* Fifteen studies from 11 countries (2003–2025) were included (14 on reporting quality, three on suicides, two measuring both). Following guideline release, reporting quality improved for “Do educate the public about the facts of suicide and suicide prevention based on accurate information” (k = 13, RR = 1.56, 95% CI = 1.30, −1.88), “Don’t describe the method used” (k = 12, RR = 1.33, 95% CI = 1.15, −1.53), and “Don’t oversimplify the reason for a suicide or reduce it to a single factor” (k = 0, RR = 1.16, 95% CI = 1.02, −1.33). For other recommendations, pooled estimates generally favored the guidelines but were often imprecise, and in some cases, had substantial heterogeneity. We could not draw firm conclusions on their impact on suicides as only three studies assessed this outcome. Contour-enhanced funnel plots and Egger’s tests suggested possible small-study effects for some recommendations. *Limitations:* All included studies included ecological before-and-after designs. Implementation strength was inconsistently reported, preventing analysis of its influence. *Conclusions:* Media guidelines appear to improve several aspects of reporting quality. Although evidence for impact on suicides is limited, guidelines are unlikely to cause any harm and may confer benefits through improved reporting standards. Further research is urgently needed to quantify their impact on suicides.

There is evidence that the media can exert a powerful influence on suicidal thoughts and behaviors. Since the 1970s, numerous studies have demonstrated that media reporting of suicide can have a harmful impact, triggering additional suicides in the general community (Asharani et al., 2024; Domaradzki, 2021; Niederkrotenthaler et al., 2020; Pirkis et al., 2018; Sisask & Värnik, 2012). The likelihood of these additional suicides occurring rises when reports of suicide are prominent and extensive (Chen et al., 2013; Etzersdorfer et al., 2001, 2004; Hassan, 1995; Ueda et al., 2014) and when they involve the death of a celebrity (Niederkrotenthaler et al., 2012) or describe the method or location in detail (Chen et al., 2013; Etzersdorfer et al., 2001, 2004; Ladwig et al., 2012; Nakamura et al., 2012). One of the largest studies in the area, which analyzed 14,638 Austrian media articles, found that high volumes of such reporting were associated with increased suicide rates (Mestas & Arendt, 2024). The harmful impact of media suicide reporting is termed the Werther effect (Phillips, 1974) after suicides that occurred in Europe following the release of Goethe’s novel *The Sorrows of Young Werther* (Goethe, 1774). There were indications that the suicide of the protagonist influenced a number of individuals in their final act (Mestas, 2023; Thorson & Oberg, 2003).

More recently, there has been an emphasis on the preventive influence that the media can have on suicide. Various studies have shown that media stories of coping, hope, and recovery from a suicidal crisis can lead to reductions in suicide and suicidal thinking, and may also promote help-seeking (Domaradzki, 2021; Niederkrotenthaler et al., 2010; Niederkrotenthaler, Till, et al., 2022). The suicide-preventive impact of the media has been called the Papageno effect (Niederkrotenthaler et al., 2010) after a character in Mozart’s opera *The Magic Flute* (Mozart, 1791) who prepares to take his own life but is convinced not to and survives.

Guidelines have been developed in many countries to assist media professionals to minimize the risk of the Werther effect and, to a lesser extent, maximize the likelihood of the Papageno effect (Maloney et al., 2014). They aim to reduce the Werther effect by discouraging sensational or detailed reporting of suicides, and to promote the Papageno effect by encouraging positive narratives of coping. Nonetheless, there remains considerable debate over how – and if – the benefits of these guidelines can be effectively measured. This is not an easy task, as it is challenging to demonstrate change by identifying that something intended to prevent an increase in suicides has led to a reduction (i.e., Werther prevention). Consequently, it may be easier to observe instances of Papageno inducement, such as Logic’s song “1-800-273-8255,” which substantially increased calls to Lifeline (Niederkrotenthaler et al., 2021). However, historically, this has not received sufficient emphasis in media guidelines.

Acknowledging this gap, leading organizations have moved to strengthen guidelines on suicide reporting. The World Health Organization and the International Association for Suicide Prevention (WHO/IASP) have produced an international set of guidelines, originally released by the WHO in 2000, then updated as a joint initiative of both organizations in 2008, 2017, and 2023 (World Health Organization & International Association for Suicide Prevention, 2023). These guidelines have evolved over time, shifting from an early focus on harm minimization to later editions that include explicit Papageno-oriented recommendations and expanded guidance on engaging with people with lived experience (World Health Organization, 2000; WHO/IASP, 2008, 2017, 2023). The recommendations in the WHO/IASP (2023) guidelines are presented in Box 1. Local guidelines are broadly consistent with the international guidelines, depending on the version on which they were based. Media guidelines are consistently recommended as an intervention in national suicide prevention strategies (Pirkis et al., 2024). They tend not to be binding, although some are embedded in the codes of practice of professional journalism bodies (Skehan et al., 2020).

**Box 1:** Recommendations in the media guidelines produced by the WHO and IASP, 2023

1	Do provide accurate information about where to seek help for suicidal thoughts and suicidal crises
2	Do educate the public about the facts of suicide and suicide prevention based on accurate information
3	Do report stories of how to cope with life stressors and/or suicidal thoughts and the importance of help-seeking
4	Do apply particular caution when reporting celebrity suicides
5	Do apply caution when interviewing bereaved family or friends or persons with lived experience
6	Do recognize that media professionals may themselves be affected by covering stories about suicide
7	Do not position suicide-related content as the top story and do not unduly repeat such stories
8	Do not describe the method used
9	Do not name or provide details about the site/location
10	Do not use language/content which sensationalizes, romanticizes, or normalizes suicide, or that presents it as a viable solution to problems
11	Do not oversimplify the reason for a suicide or reduce it to a single factor
12	Do not use sensational language in headlines
13	Do not use photographs, video footage, audio recordings, digital or social media links
14	Do not report the details of a suicide note

A number of studies have evaluated the impact of these media guidelines, typically considering whether the quality of suicide-related media reporting improved once guidelines were introduced. A subset of these evaluations have gone one step further and considered whether the introduction of the guidelines has been associated with decreases in suicide. There have been two reviews of these studies, a systematic review by Bohanna and Wang (2012) and a narrative review by Stack (2020). Bohanna and Wang (2012) concluded that the evidence for the effectiveness of media guidelines was mixed, and Stack (2020) argued that greater attention needed to be paid to which specific guideline recommendations might be effective (or ineffective). Neither of the two reviews summarized data from the relevant studies in a meta-analytic way, and additional studies have been done that were not included in either review. We therefore conducted this meta-analysis to bring together the most up-to-date corpus of studies. Our aim was to assess the overall effect of releasing media guidelines for reporting of suicide on the quality of suicide-related reporting and suicide outcomes, to progress knowledge about whether media guidelines have an impact on reporting quality and suicides.

## Methods

This meta-analysis follows the preferred reporting items for systematic reviews and meta-analyses (PRISMA) guidelines (Page et al., 2021). The protocol was pre-registered in PROSPERO (CRD42023467588). Amendments to the protocol are described in detail on page 1 of the Electronic Supplementary Material 1 (ESM 1).

### Search Strategy and Selection Criteria

We searched PubMed, Scopus, Embase, PsycInfo, and Web of Science up to February 10, 2025, with a search update conducted on November 17, 2025. We used English-language terms related to media guidelines and suicide (ESM 1, page 2). We also searched Google Scholar on the same date using the query “suicide and media” to identify grey literature. In addition, we screened reference lists and citations of relevant studies and reviews and consulted members of the *Suicide and the Media* Special Interest Group of the International Association for Suicide Prevention. Detailed information on the search strategy for all sources is provided in ESM 1 (page 2).

SSR and MU or VA independently screened titles, abstracts, and full-texts using https://covidence.org, resolving disagreements by consensus or consulting the team.

Our eligibility criteria were structured around the Population, Intervention, Comparison, Outcomes, and Study (PICOS) framework. We included studies that:

• Analyzed data from the general population (P).
• Evaluated media guidelines on suicide-related reporting (I). It could involve the release of media guidelines with implementation efforts (e.g., workshops distributing the guidelines) or without implementation efforts.
• Compared at least one pre- and postguideline time-point (C).
• Measured reporting quality (proportion of suicide-related media items adhering to media guidelines) or suicide rates pre- and postguidelines (O).
• Were ecological studies that used before-and-after designs or involved time series analyses (S).

Studies that examined the same dataset had the most recent data selected. If the year of guideline release was unclear, the study was excluded as pre- and post-timepoints could not be determined. Studies were excluded if the intervention aimed to improve suicide-related media reporting but was unrelated to media guidelines (e.g., government appeals).

### Data Extraction

For each study, we recorded country, guideline year, implementation evidence, pre/post period length, and data source. For the reporting quality outcome, we extracted the number of guideline recommendations or media items (including total assessed) per timepoint. For the suicide outcome, we extracted annual suicides and population per timepoint.

Across the included studies, adherence to media guidelines was operationalized as the presence or absence of individual guideline items and compared across time points. Most, although not all, studies reported double coding with interrater reliability checks. Several structured coding systems are available for assessing the quality of suicide-related media reporting and can be adapted to different guideline frameworks, including two validated instruments – the Risk of Imitative Suicide Scale (Nutt et al., 2015) and PRINTQUAL (John et al., 2014) – which align closely with WHO/IASP (2023) media reporting guideline recommendations, as well as other standardized coding schemes (Niederkrotenthaler, Laido, et al., 2022). However, none of the included studies reported using these systems, and we relied on adherence measures as defined in each primary study.

We contacted corresponding authors for clarification when needed. In one study (Sinyor et al., 2024), we adjusted the suicide outcome data to standardize the annual reporting period. In another study (Niederkrotenthaler & Sonneck, 2007), data prior to 1970 and the total number of recommendations (media items) assessed were unavailable; therefore, we included only the post-1970 suicide outcome data. For the reporting quality outcome, each individual guideline recommendation was mapped to the most closely corresponding WHO/IASP 2023 guideline recommendation (WHO/IASP, 2023), based on wording similarity (see ESM 1, pages 3–12). Where multiple study recommendations aligned with the same WHO/IASP recommendation, one was randomly selected to avoid double-counting. Data were recoded where necessary, as shown in the ESM 1 (pages 3–12). Data extraction began on June 1, 2024, and was independently conducted by SSR and VA, with discrepancies resolved by JP.

### Risk of Bias and Synthesis Methods

Publication bias was evaluated using contour-enhanced funnel plots and Egger’s regression test for funnel plot asymmetry in the study-specific estimates.

For studies with multiple pre- or postguideline timepoints (Eriyanto, 2025; Garrido-Fabián et al., 2018; Ju et al., 2024; Ramadas & Kuttichira, 2011), we only analyzed the first timepoint immediately following guideline introduction to reduce the influence of unrelated factors.

We conducted two primary analyses, one for each outcome. For the reporting quality outcome, we examined adherence to 13 of the 14 recommendations from the WHO/IASP media guidelines (WHO/IASP, 2023). We excluded the recommendation “Do recognize that media professionals may themselves be affected by covering stories about suicide” as none of the included studies assessed this recommendation as it relates to journalist wellbeing rather than reporting quality. For each eligible study, we calculated the proportion of media items that adhered to the 13 recommendations pre and post the introduction of the guidelines. We then estimated the pooled risk ratio (RR) and 95% confidence interval (95% CI) to quantify the relative change in adherence to the guidelines following their release, using a random-effects model.

For the studies that presented data on the suicide outcome, we calculated yearly suicide rates per 100,000 people pre and post the introduction of the guidelines. To quantify the relative change in suicides, we calculated the incidence rate ratio (IRR) and 95% CI, comparing post- to preintervention periods using a random-effects model.

We conducted a sensitivity analysis for each outcome, stratifying studies by whether there was a clear gap between the introduction of the guidelines and the start of the postintervention period. We did this to investigate whether studies that allowed time for the guidelines to be fully implemented yielded different results to those that did not, and to mitigate any potential bias arising from our decision to use the immediate timepoint after guideline introduction in our primary analysis.

Heterogeneity across all models was evaluated using the *I*^*2*^ statistic, with values of 25%, 50%, and 75% considered indicative of low, moderate, and high heterogeneity, respectively (Borenstein et al., 2021). All analyses were conducted in R (version 4.4.2), including use of the *metafor* package (Viechtbauer, 2010).

## Results

**Figure 1:**
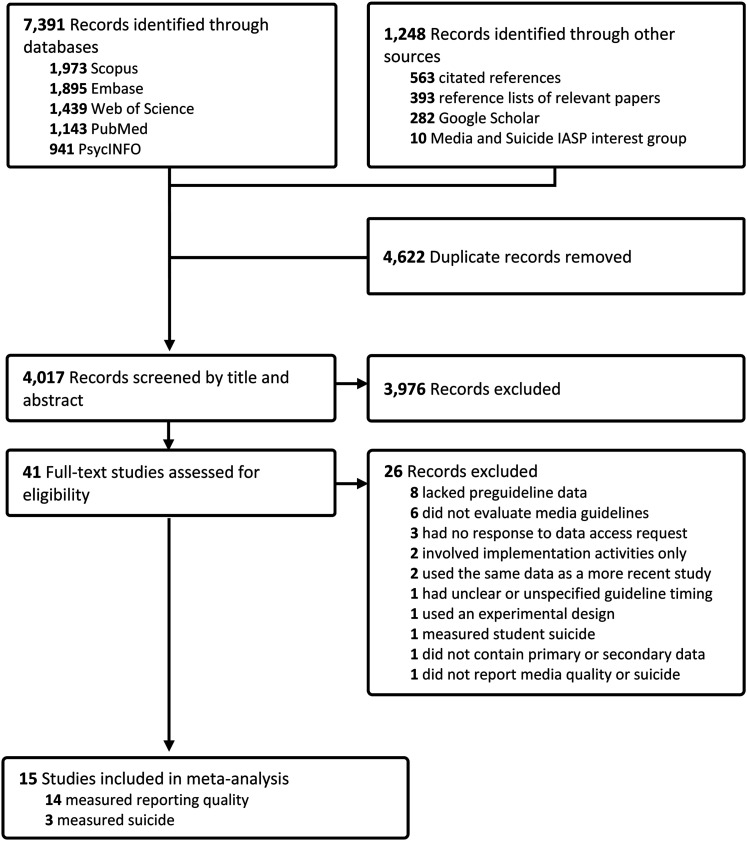
PRISMA flow diagram.

We initially identified 8,639 records: 7,391 from our database search and 1,248 references from other sources (see Figure 1). After removing duplicates, we screened 4,017 titles and abstracts and retrieved 41 for full-text eligibility. Of these, 26 were excluded, leaving 15 studies for inclusion in the review (Acosta et al., 2020; Armstrong et al., 2025; Eriyanto, 2025; Fu & Yip, 2008; Garrido-Fabián et al., 2018; Jamieson et al., 2003; Ju et al., 2024; Kim, 2005; Niederkrotenthaler & Sonneck, 2007; Pirkis et al., 2009; Ramadas & Kuttichira, 2011; Roskar et al., 2017; Shaw et al., 2025; Sinyor et al., 2021; Sinyor et al., 2024). For a list of excluded studies (with reasons) and included studies, see ESM 1, pages 13–14 and 15–16.

Characteristics of the 15 studies are presented in Table 2. The studies were published between 2003 (Jamieson et al., 2003) and 2025 (Armstrong et al., 2025; Eriyanto, 2025; Shaw et al., 2025), and were conducted in 11 different countries or regions: Australia (Pirkis et al., 2009); Austria (Niederkrotenthaler & Sonneck, 2007); Canada (Sinyor et al., 2021; Sinyor et al., 2024); Hong Kong (Fu & Yip, 2008); India (Armstrong et al., 2025; Ramadas & Kuttichira, 2011); Slovenia (Roskar et al., 2017); South Korea (Ju et al., 2024; Kim, 2005); Spain (Acosta et al., 2020; Garrido-Fabián et al., 2018); Guyana (Shaw et al., 2025); Indonesia (Eriyanto, 2025); and the United States (Jamieson et al., 2003). Where a country contributed more than one study, samples did not duplicate one another. For example, the Canadian studies examined different interventions: Sinyor et al. (2021) evaluated the 2009 Canadian guideline, whereas Sinyor et al. (2024) assessed the 2018 Canadian guideline update accompanied by targeted implementation efforts. Similarly, the Spanish studies drew on different guideline versions, with Garrido-Fabián et al. (2018) assessing reporting quality under the WHO 2000 guidelines, and Acosta et al. (2020) examining reporting quality following the WHO/IASP 2017 update after local dissemination efforts. The South Korean studies also used different media outlets and periods. Kim (2005) examined adherence to local guidelines in print newspapers using an immediate pre–post design, whereas Ju et al. (2024) analyzed reporting quality in digital news outlets, with a substantial gap between the guideline release and the postguideline assessment period. A similar pattern was observed among the studies from Indian where Ramadas and Kuttichira (2011) evaluated a locally developed guideline derived from the WHO 2000 recommendations and implemented through a journalist workshop in Kerala, while Armstrong et al. (2025) assessed the national *Suicide Reporting Guideline* released by the Press Council of India in 2019, drawing on broader, system-level monitoring and engagement initiatives.

**Table 1:** Study characteristics of include studies

Study	Country	Guidelines, year of release^a^	Evidence of implementation activities, as described in the study	Outcome measured	Preintervention Outcome assessment period	Postintervention Outcome assessment period	Is there any gap between release of guidelines and start of postintervention assessment period?
Acosta et al. (2020)	Spain	WHO/IASP guidelines (Preventing Suicide: A Resource for Media Professionals, Update 2017), 2017	Training course for media professionals and dissemination of WHO/IASP guidelines by the Mental Health Service, in collaboration with the Ministry of Health Press Office in the Canary Islands	Quality of reporting^b^	6 months (November 1, 2016–April 30, 2017)	6 months (December 15, 2017–June 13, 2018)	Yes: 7.5 months (May 1, 2017–December 14, 2017)
Armstrong et al. (2025)	India	Indian media guidelines (Suicide Reporting Guideline by Press Council of India), 2019	Project Siren (a national-level media monitoring project focused on suicide reporting in English-language print and online media); a SNEHA-hosted forum on media and suicide; and integration of suicide-reporting training within a prominent college of journalism and research studies	Quality of reporting^d^	7 months (June 1, 2016–December 31, 2016)	6 months (July 1, 2023–December 31, 2023)	Yes: 42 months/3.5 years (2019–June 30, 2023)
Eriyanto (2025)	Indonesia	Indonesian media guidelines (Guidelines for Reporting on Acts and Attempts of Suicide), 2019	No description of implementation activities	Quality of reporting^b^	12 months (2018)	12 months (2020)	Yes: up to 12 months
Fu and Yip (2008)	Hong Kong	Hong Kong guidelines (Recommendations on Suicide Reporting for Media Professionals), 2004	Public seminar and press conference at the University of Hong Kong, followed by distribution of guidelines to media professionals in Hong Kong.	Quality of reporting^c^	10 months (January 1, 2004–November 8, 2004)	19 months (November 9, 2004–June 30, 2006)	No
Garrido-Fabián et al. (2018)	Spain	WHO guidelines (Preventing Suicide: A Resource for Media Professionals), 2000	No description of implementation activities.	Quality of reporting^b^	12 months (1995)	12 months (2000)	No
Jamieson et al. (2003)	United States	Centres for Disease Control and Prevention (CDC Recommendations for Suicide Reporting), 1994	No description of implementation activities	Quality of reporting^b^	12 months (1990)	12 months (1995)	No
Ju et al. (2024)	South Korea	Korean guidelines (Suicide Reporting Guideline 1.0), 2004	No description of implementation activities	Quality of reporting^b^	6 months (January 2004 to June 2004)	6 months (January 2013 to June 2013)	Yes: 102 months/8.5 years (July 2004 to December 2012)
Kim (2005)	South Korea	Korean media guidelines (Suicide Reporting Guideline 1.0), 2004	No description of implementation activities	Quality of reporting^d^	8 months (December 2003–July 2004)	8 months (July 2004–March 2005)	No
Niederkrotenthaler and Sonneck (2007)	Austria	Austrian media guidelines (Media Guidelines for the Reporting on Suicides), 1987	Dissemination of the guidelines in Vienna and in different parts of Austria with strong or weak media collaboration.	Suicide^e,h^	204 months/ 17 years (1970/71–1986/87)	216 months/ 18 years (1987/88–2004/05)	No
Pirkis et al. (2009)	Australia	Australian media guidelines (Reporting Suicide and Mental Illness), 2002	Guideline disseminated through the Mindframe Media and Mental Health Project using face-to-face briefings, drop-in visits, ad hoc advice, distribution of materials, and follow-up support for media professionals	Quality of reporting^c^	12 months (1 March 2000 to 28 February 2001)	12 months (September 1, 2006–August 31, 2007	Yes: 78 months/6.5 years (March 1, 2001–August 31, 2006)
Ramadas and Kuttichira (2011)	India	Local 15-item media guideline based on the WHO (2000) media guidelines (Preventing Suicide: A Resource for Media Professionals), 2000	Workshop with journalists with active collaboration of media and mental health professionals.	Quality of reporting^d^	12 months (2000–2001)	12 months (2001–2002)	No
Roskar et al. (2017)	Slovenia	Slovenian media guidelines (Let’s Talk About Suicide and the Media: Suicide Prevention – Professional Guidelines for Responsible Journalistic Reporting), 2010	Workshop held with local media representatives in each of Slovenia’s nine health regions. Guidelines sent to the Journalists Honour Court and The Chamber of Slovenian Journalists for further dissemination	Quality of reporting^b^	12 months (1 May 2009–30 April 2010)	12 months (1 May 2011–30 April 2012)	No
Shaw et al. (2025)	Guyana	Guyanese media guidelines (Guidelines for Responsible Reporting on Suicides in Guyana), 2021	One-day consultation workshop with the editors of all major media companies	Quality of reporting^b^	12 months (September 2020–August 2021)	12 months (October 2021–September 2022)	No
Sinyor et al. (2021)	Canada	Canadian media guidelines (Media Guidelines for Reporting Suicide), 2009	No description of implementation activities	Quality of reporting^c^	84 months/7 years (2002–2008)	84 months/7 years (2009–2015)	No
				Suicide^f^	84 months/7 years (2002–2008)	84 months/7 years (2009 to 2015)	No
Sinyor et al. (2024)	Canada	Canadian media guidelines (Media Guidelines for Reporting on Suicide: 2017 Update), 2018	Guideline disseminated through forums with international experts, symposia at Canadian Psychiatric Association annual meetings, presentation at the Canadian Association of Journalists annual meeting, and ongoing informal discussions with media professionals	Quality of reporting^c^	72 months/6 years (November 2009–November 5, 2015)	34 months/∼3 years (November 10, 2018–October 2021)	No
				Suicide^f^	108 months/9 years (January 2010–December 2018)^g^	36 months/3 years (January 2019–December 2021)^g^	No
*Note.* ^a^Year of guideline release as relevant to the outcome of interest. ^b^Source: Digital version of newspaper articles. ^c^Source: Print and digital versions of newspaper articles. ^d^Source: Print version of newspaper articles. ^e^Source: Statistics Austria. ^f^Source: Office of the Chief Coroner of Ontario. ^g^The preintervention period originally ran from November 2009, and the postintervention period originally ended in October 2021; data were analyzed from January 2010 to December 2018 (preintervention) and from January 2019 to December 2021 (postintervention). ^h^Niederkrotenthaler & Sonneck, 2007 also measured reporting quality, but total media counts were unavailable, and 1946–1969 suicide data were missing.							

**Table 2:** Summary of pooled results for primary analyses

Outcome	k	Pooled RR/IRR [95% CI]	*I* ^2^	Favors guidelines
Quality of reporting				
Do provide accurate information about where to seek help for suicidal thoughts and suicidal crises	8	RR = 3.48 [0.92, −13.10]	95.3%	Yes
Do educate the public about the facts of suicide and suicide prevention based on accurate information	13	RR = 1.56 [1.30, −1.88]	20.6%	Yes
Do report stories of how to cope with life stressors and/or suicidal thoughts and the importance of help-seeking	7	RR = 2.04 [0.83, −5.03]	79.3%	Yes
Do apply particular caution when reporting celebrity suicides	5	RR = 1.79 [0.82, −3.91]	99.7%	Yes
Do apply caution when interviewing bereaved family or friends or persons with lived experience	5	RR = 1.03 [1.00, −1.06]	41.9%	Yes
Do not position suicide-related content as the top story and do not unduly repeat such stories	6	RR = 0.98 [0.78, −1.12]	92.8%	No
Do not describe the method used	12	RR = 1.33 [1.15, −1.53]	79.8%	Yes
Do not name or provide details about the site/location	7	RR = 1.12 [0.90, −1.38]	77.8%	Yes
Do not use language/content which sensationalizes, romanticizes, or normalizes suicide, or that presents it as a viable solution to problems	11	RR = 1.12 [0.98, −1.29]	98.8%	Yes
Do not oversimplify the reason for a suicide or reduce it to a single factor	10	RR = 1.16 [1.02, −1.33]	86.1%	Yes
Do not use sensational language in headlines	12	RR = 1.06 [0.99, −1.13]	60.1%	Yes
Do not use photographs, video footage, audio recordings, digital or social media links	11	RR = 0.99 [0.94, −1.04]	92.0%	No
Do not report the details of a suicide note	6	RR = 0.96 [0.93, −1.00]	40.5%	No
Suicides	3	IRR = 0.97 [0.84, −1.11]	98.6%	Yes
*Note*. k = number of studies contributing to each pooled estimate. RR = risk ratio; IRR = incidence rate ratio; CI = confidence interval. Effect sizes compare post-guideline with pre-guideline periods and are pooled across studies. For the quality of reporting outcomes, RR >1 indicates greater adherence post-guideline. For the suicide outcome, IRR <1 indicates fewer deaths post-guideline. *I*^*2*^ quantifies between-study heterogeneity. “Favors guidelines” reflects the direction of the point estimate (RR >1 for reporting quality; IRR <1 for suicides).				

Most studies examined the implementation of local guidelines (Armstrong et al., 2025; Eriyanto, 2025; Fu & Yip, 2008; Jamieson et al., 2003; Ju et al., 2024; Kim, 2005; Niederkrotenthaler & Sonneck, 2007; Pirkis et al., 2009; Ramadas & Kuttichira, 2011; Roskar et al., 2017; Shaw et al., 2025; Sinyor et al., 2021; Sinyor et al., 2024), but the two Spanish studies considered the WHO/IASP guidelines (Acosta et al., 2020; Garrido-Fabián et al., 2018). In many cases, implementation activities relating to the dissemination of the guidelines were described; these typically involved awareness-raising activities and training and support for media professionals, often delivered via workshops or other engagement forums.

Fourteen of the studies assessed the reporting quality outcome (Acosta et al., 2020; Armstrong et al., 2025; Eriyanto, 2025; Fu & Yip, 2008; Garrido-Fabián et al., 2018; Jamieson et al., 2003; Ju et al., 2024; Kim, 2005; Pirkis et al., 2009; Ramadas & Kuttichira, 2011; Roskar et al., 2017; Shaw et al., 2025; Sinyor et al., 2021; Sinyor et al., 2024), and three examined suicide (Niederkrotenthaler & Sonneck, 2007; Sinyor et al., 2021; Sinyor et al., 2024), with two of these considering both outcomes (Sinyor et al., 2021; Sinyor et al., 2024). All studies assessing reporting quality used before-and-after designs, whereas the three suicide-outcome studies used interrupted or ecological time-series designs. These time-series analyses accounted for underlying time trends and autocorrelation; the Canadian studies additionally adjusted for unemployment and consumer price index, and the most recent also controlled for population size.

The shortest pre- and postintervention outcome assessment periods were six months (Acosta et al., 2020; Ju et al., 2024), and the longest were about 17–18 years (Niederkrotenthaler & Sonneck, 2007). For five of the studies, all assessing the reporting quality outcome, there was a gap between the implementation of the guidelines and the beginning of the postintervention data collection period (Acosta et al., 2020; Armstrong et al., 2025; Eriyanto, 2025; Ju et al., 2024; Pirkis et al., 2009); these gaps ranged from 7.5 months (Acosta et al., 2020) to 8.5 years (Ju et al., 2024). We conducted a sensitivity analysis to account for the potential impact of these gaps, and the results are described below.

Table 2 summarizes the results of our 14 primary analyses (13 for the reporting quality outcome and one for the suicide outcome; their forest plots are available on pages 17–24 in ESM 1). Overall, we found evidence of improved reporting following the introduction of media guidelines for three WHO/IASP recommendations: “Do educate the public about the facts of suicide and suicide prevention based on accurate information” (k = 13; RR = 1.56; 95% CI = 1.30, −1.88), “Don’t describe the method used” (k = 12; RR = 1.33; 95% CI = 1.15, −1.53), and “Don’t oversimplify the reason for a suicide or reduce it to a single factor” (k = 10; RR = 1.16; 95% CI = 1.02, −1.33). For the remaining recommendations, we did not observe evidence of change; however, several of these estimates should be interpreted cautiously given their wide confidence intervals. We also did not find evidence of a reduction in suicides; however, it is worth noting that only three studies assessed this outcome. In the main, pooled estimates tended to favor the guidelines. Heterogeneity in these models was low to considerable, with *I*^2^ ranging from 20.6% to 99.7% for reporting quality and 98.6% for the suicide outcome, as shown in Table 2.

A summary of the sensitivity analysis findings related to reporting quality and suicides is provided on pages 25–26 in ESM 1, with the corresponding forest plots on pages 27–39. Sensitivity analyses stratified by the presence of a gap between guideline implementation and postintervention assessment produced broadly similar effect estimates to the primary analyses, with “Don’t describe the method used” and “Don’t oversimplify the reason for a suicide or reduce it to a single factor” remaining consistently associated with improved reporting, while some effects (e.g., educating the public and omitting site details) appeared stronger in studies with no implementation gap compared to those with implementation gap and several outcomes became less precise due to smaller numbers of studies.

Contour-enhanced funnel plots for each outcome in the primary and sensitivity analyses were inspected for evidence of small-study effects (see ESM 1, pages 40–60). In the primary analysis of reporting quality, some plots appeared asymmetrical, and Egger’s tests were significant for the recommendations “Don’t oversimplify the reason for a suicide or reduce it to a single factor” (k = 10, *p* = .002) and “Don’t use sensational language in headlines” (k = 12, *p* = .012), suggesting possible small-study effects. We only interpreted Egger’s regression for meta-analyses with at least 10 studies; in the sensitivity analyses, and for the suicide outcome and other recommendations with fewer than 10 studies, Egger’s test was not considered reliable (Sterne et al., 2011) and is not interpreted further.

## Discussion

Our meta-analysis provides evidence that media guidelines can improve the quality of suicide-related media reporting. However, more research is needed to reach firmer conclusions, particularly regarding their impact on suicide rates, as only three studies assessed this outcome. In the meta-analysis, the point estimates tended to favor the guidelines in most cases.

Some guideline recommendations in our primary analysis were associated with improvements in suicide-related media reporting. These are important because they concern content shown to be particularly likely to prevent the Werther effect. For instance, the recommendation “Don’t describe the method used” is critical because explicitly detailing suicide methods can substantially increase the risk of further suicides by that method, as shown in cases involving charcoal burning (Chen et al., 2013), hydrogen sulphide ingestion (Nakamura et al., 2012), and jumping in front of trains (Etzersdorfer et al., 2004; Ladwig et al., 2012).

Similarly, we found evidence for the recommendations “Do educate the public about the facts of suicide and suicide prevention based on accurate information” and “Don’t oversimplify the reason for a suicide or reduce it to a single factor.” These recommendations are important because they address widespread misconceptions that can shape public understanding and behavior, as supported by evidence showing that mass media can help to reduce stigma and improve help-seeking among people at risk of suicide (Niederkrotenthaler et al., 2014).

However, these clearer effects were not observed for the remaining recommendations, and the apparent lack of impact should be interpreted cautiously. For several recommendations, the pooled risk ratios had wide confidence intervals and, in some cases, substantial heterogeneity, indicating that the corresponding estimates are likely imprecise. This means that although we observed stronger and more consistent evidence for a small number of recommendations, the overall pattern across all items is better viewed as suggestive rather than definitive.

One factor that may help explain the variability in effects is the intensity and quality of implementation efforts for guidelines. Studies examining journalists’ responses suggest that guidelines accompanied by a strong implementation strategy are more likely to resonate with, and be used by, media professionals (Gandy & Terrion, 2015; Skehan et al., 2006; Skehan et al., 2025). We attempted to extract details from the original studies on any implementation activities that accompanied the release of guidelines (e.g., training sessions, workshops) but were unable to do so confidently because we could not be sure that none occurred if they were not mentioned in the study (we did not want to treat absence of evidence as evidence of absence). As a result, we were unable to analyze the potential effect of implementation strength as initially planned. Without a reliable measure of implementation intensity, it is challenging to differentiate between the intrinsic effectiveness of the guidelines and the effectiveness of their dissemination.

In a similar vein, future research should consider the extent to which journalists and editors are involved in the development and roll-out of guidelines from the outset. In discussing why the evidence of effectiveness for media guidelines is mixed, Bohanna and Wang (2012) noted that improvements in reporting and prevention of subsequent suicides tended to occur when media professionals are engaged throughout, an observation reinforced by Notredame and colleagues (Notredame et al., 2016). As the authors of several of the included studies, we can comment on the collaborative efforts that are likely to have influenced the results. Australia’s guidelines were associated with improved compliance with recommendations on which other guidelines did not fare so well (e.g., “Do apply particular caution when reporting celebrity suicides”). This may be because Australia’s guidelines were developed (and have been regularly updated) with media professionals’ input and are actively disseminated to journalists (Pirkis et al., 2009; Skehan et al., 2006). Similarly, the Austrian guidelines led to the largest observed reduction in suicides in our meta-analysis, and the impact was most evident in regions with strong collaboration between guideline developers and media professionals (Niederkrotenthaler & Sonneck, 2007). The Canadian guidelines did not achieve the same success, although the results were more positive in Ontario than in Canada as a whole. This is likely to reflect implementation struggles and opposition from some media professionals in some parts of the country (Sinyor et al., 2021; Sinyor et al., 2024).

### Strengths and Limitations

This meta-analysis is the first to systematically bring together data from studies that used pre- and postguideline data to assess the impact of media guidelines on the quality of suicide-related media reporting and suicide rates. By explicitly focusing on changes before and after the release of guidelines, it offers a novel and valuable contribution to the field. However, it had several limitations.

A major limitation is that all included studies, by necessity, used ecological pre–post designs. This restricted the extent to which any observed changes in outcomes can be confidently attributed to the introduction of the guidelines. Another important limitation, as noted earlier, was our inability to reliably assess the level or intensity of implementation efforts across studies. This constraint reduced our capacity to determine whether improvements in outcomes reflected the guidelines themselves or the effectiveness of their dissemination activities. As a result, interpreting the real-world impact of media guidelines remains challenging without considering how they are implemented in practice.

Another key challenge relates to the subjective nature of evaluating reporting quality against guideline recommendations. For example, determining whether a story “oversimplifies the reason for a suicide” or “educates the public” can involve interpretation rather than objective coding. This challenge applies both to the methods used in the included studies and to our process of mapping recommendations from individual study guidelines onto the WHO/IASP (2023) framework. While necessary for synthesis, this process may have introduced some subjectivity into how adherence was classified. As already noted, several structured coding systems with demonstrated intercoder reliability are available and can be used and adapted to assess the quality of suicide-related media reporting (John et al., 2014; Niederkrotenthaler, Laido, et al., 2022; Nutt et al., 2015), but none of the studies in this review used these more standardized approaches. Future studies should consider applying and, where necessary, adapting such coding systems to improve consistency and reduce subjectivity in assessments.

Several studies were based on earlier versions of the WHO/IASP guidelines. While most core recommendations have remained consistent over time, some differences in findings may reflect changes in guideline content rather than inconsistencies in implementation or reporting. Still, this mapping approach is unlikely to be the primary source of the observed heterogeneity. It is more plausible that contextual differences across studies (e.g., national setting and strength of implementation strategies) played an important role. Due to limited amount of data, we were unable to carry out the planned subgroup analyses to explore these potential sources of heterogeneity, as doing so would have further reduced statistical power. As the evidence base grows, future syntheses will be better placed to address these questions.

### Conclusion

Overall, media guidelines show promise for improving suicide-related media reporting, particularly those known to prevent the Werther effect. Although only a limited number of studies have directly assessed their impact on suicide rates, the evidence suggests that they cause no harm and likely confer benefits through better reporting practices. Media guidelines represent a relatively simple and good value intervention with considerable potential (Flego et al., 2022) and should remain a key component of national suicide prevention strategies (Pirkis et al., 2024). However, more research is urgently needed to better understand their impact on suicides more precisely.

## Electronic Supplementary Material

The following electronic supplementary material is available at https://doi.org/10.1027/0227-5910/a001049**ESM 1**. PROSPERO protocol; search strategy; guideline recommendations compared to WHO/IASP 2023 guidelines; excluded and included studies; primary and sensitivity analysis forest plots; pooled results for sensitivity analysis; primary and sensitivity analysis funnel plots.

## Notes

**Publication Ethics:** Ethical approval was not required for this study, as it is a systematic review and meta-analysis of previously published data.

Thomas Niederkrotenthaler is the editor-in-chief of the journal. He was not involved in the peer review and editorial evaluation of the manuscript prior to acceptance. Benedikt Till handled the peer review of this paper.

**Authorship:** Simone Scotti Requena, methodology, software, validation, formal analysis, investigation, data curation, project administration, writing – original draft, writing – review & editing; Vikas Arya, methodology, software, validation, formal analysis, investigation, data curation, writing – review & editing; Thomas Niederkrotenthaler, conceptualization, methodology, investigation, writing – review & editing; MS, conceptualization, methodology, writing – review & editing; Michiko Ueda, conceptualization, methodology, investigation, writing – review & editing; Matthew J. Spittal, conceptualization, methodology, software, supervision, writing – review & editing; Jane Pirkis, conceptualization, methodology, investigation, supervision, writing – original draft, writing – review & editing.

**Open Science:** Data extracted from the reviewed studies are provided in the main manuscript or electronic supplementary material (ESM 1). Additional aggregated suicide counts, obtained from the corresponding authors of relevant studies, are available upon reasonable request.

The study protocol was preregistered on PROSPERO (CRD42023467588) and can be found here https://www.crd.york.ac.uk/PROSPERO/view/CRD42023467588.
