# Supplementary material for: Impact of Media Guidelines on Suicide-Related Reporting Quality and Suicides: A Systematic Review and Meta-Analysis
Source: Crisis. 2026 Feb 13;47(3):188–200. doi: 10.1027/0227-5910/a001049 (PMC13231429; doi:10.1027/0227-5910/a001049)

Electronic Supplementary Material 1

to

Impact of Media Guidelines on Suicide-Related Reporting Quality and Suicides: A Systematic Review and Meta-analysis

https://doi.org/10.1027/0227-5910/a001049

© 2026 The Author(s). Distributed as a Hogrefe OpenMind article under the license CC BY 4.0 (https://creativecommons.org/licenses/by/4.0)

Simone Scotti Requena, PhD

Vikas Arya, PhD

Thomas Niederkrotenthaler, PhD

Mark Sinyor, MD

Michiko Ueda, PhD

Matthew J. Spittal, PhD

Jane Pirkis, PhD

Table of Contents

PROSPERO 1
Detailed search strategy 2
eTable 1. Full list of each individual recommendation from the guidelines used in any given study, mapped against the recommendations in the WHO/IASP 2023 guidelines 3
Studies excluded after full-text screening 13
Studies included in the meta-analysis 15
Primary analysis forest plot – suicide rates outcome 17
Primary analysis forest plots – quality of suicide-related media reporting outcome 18
- Do provide accurate information about where to seek help for suicidal thoughts and suicidal crises 18
- Do educate the public about the facts of suicide and suicide prevention based on accurate information 18
- Do report stories of how to cope with life stressors and/or suicidal thoughts and the importance of help-seeking 19
- Do apply particular caution when reporting celebrity suicides 19
- Do apply caution when interviewing bereaved family or friends or persons with lived experience 20
- Don't position suicide-related content as the top story and don't unduly repeat such stories 20
- Don't describe the method used 21
- Don't name or provide details about the site/location 21
- Don't use language/content which sensationalizes, romanticizes or normalizes suicide, or that presents it as a viable solution to problems 22
- Don't oversimplify the reason for a suicide or reduce it to a single factor 22
- Don't use sensational language in headlines 23
- Don't use photographs, video footage, audio recordings, digital or social media links 23
- Don't report the details of a suicide note 24

eTable 2. Summary of pooled results for sensitivity analysis 25
eTable 2 (cont): Summary of pooled results for sensitivity analysis 26
Sensitivity analysis forest plots – suicide rates outcome 27
- Studies with no gap between guideline release and the start of the post-intervention assessment period 27

Sensitivity analysis forest plots – quality of suicide-related media reporting outcome 28
- Studies with a gap between guideline release and the start of the post-intervention assessment period 28
  - Do provide accurate information about where to seek help for suicidal thoughts and suicidal crises 28
  - Do educate the public about the facts of suicide and suicide prevention based on accurate information 28
  - Do report stories of how to cope with life stressors and/or suicidal thoughts and the importance of help-seeking 28
  - Do apply particular caution when reporting celebrity suicides 29
  - Do apply caution when interviewing bereaved family or friends or persons with lived experience 29
  - Don't position suicide-related content as the top story and don't unduly repeat such stories 29
  - Don't describe the method used 30
  - Don't name or provide details about the site/location 30
  - Don't use language/content which sensationalizes, romanticizes or normalizes suicide, or that presents it as a viable solution to problems 31
  - Don't oversimplify the reason for a suicide or reduce it to a single factor 31
  - Don't use sensational language in headlines 32
  - Don't use photographs, video footage, audio recordings, digital or social media links 32
  - Don't report the details of a suicide note 33

Studies with no gap between guideline release and the start of the post-intervention assessment period....34
Do provide accurate information about where to seek help for suicidal thoughts and suicidal crises 34
Do educate the public about the facts of suicide and suicide prevention based on accurate information....34
Do report stories of how to cope with life stressors and/or suicidal thoughts and the importance of help-seeking....35
Do apply particular caution when reporting celebrity suicides....35
Do apply caution when interviewing bereaved family or friends or persons with lived experience ...36
Don't position suicide-related content as the top story and don't unduly repeat such stories....36
Don't describe the method used....36
Don't name or provide details about the site/location....37
Don't use language/content which sensationalizes, romanticizes or normalizes suicide, or that presents it as a viable solution to problems....37
Don't oversimplify the reason for a suicide or reduce it to a single factor....38
Don't use sensational language in headlines....38
Don't use photographs, video footage, audio recordings, digital or social media links....39
Don't report the details of a suicide note....39
Primary analysis contour-enhanced funnel plots – suicide rates outcome....40
Primary analysis contour-enhanced funnel plots – quality of suicide-related media reporting outcome ...41
Do provide accurate information about where to seek help for suicidal thoughts and suicidal crises....41
Do educate the public about the facts of suicide and suicide prevention based on accurate information....41
Do report stories of how to cope with life stressors and/or suicidal thoughts and the importance of help-seeking....42
Do apply particular caution when reporting celebrity suicides....42
Do apply caution when interviewing bereaved family or friends or persons with lived experience....43
Don't position suicide-related content as the top story and don't unduly repeat such stories....43
Don't describe the method used....44
Don't name or provide details about the site/location....44
Don't use language/content which sensationalizes, romanticizes or normalizes suicide, or that presents it as a viable solution to problems....45
Don't oversimplify the reason for a suicide or reduce it to a single factor....45
Don't use sensational language in headlines....46
Don't use photographs, video footage, audio recordings, digital or social media links....46
Don't report the details of a suicide note....47
Sensitivity analysis contour-enhanced funnel plots – suicide outcome....48
Studies with no gap between guideline release and the start of the post-intervention assessment period....48
Sensitivity analysis contour-enhanced funnel plots – quality of suicide-related media reporting outcome 49
Studies with a gap between guideline release and the start of the post-intervention assessment period....49
Do provide accurate information about where to seek help for suicidal thoughts and suicidal crises 49
Do educate the public about the facts of suicide and suicide prevention based on accurate information....50
Do report stories of how to cope with life stressors and/or suicidal thoughts and the importance of help-seeking....50
Do apply particular caution when reporting celebrity suicides....50
Do apply caution when interviewing bereaved family or friends or persons with lived experience ...50
Don't position suicide-related content as the top story and don't unduly repeat such stories....51
Don't describe the method used....51

Don’t name or provide details about the site/location 52
Don’t use language/content which sensationalizes, romanticizes or normalizes suicide, or that presents it as a viable solution to problems 52
Don’t oversimplify the reason for a suicide or reduce it to a single factor 53
Don’t use sensational language in headlines 53
Don’t use photographs, video footage, audio recordings, digital or social media links 54
Don’t report the details of a suicide note..54

Studies with no gap between guideline release and the start of the post-intervention assessment period...55

Do provide accurate information about where to seek help for suicidal thoughts and suicidal crises55
Do educate the public about the facts of suicide and suicide prevention based on accurate information 55
Do report stories of how to cope with life stressors and/or suicidal thoughts and the importance of help-seeking 56
Do apply particular caution when reporting celebrity suicides...56
Do apply caution when interviewing bereaved family or friends or persons with lived experience ...57
Don’t position suicide-related content as the top story and don’t unduly repeat such stories 57
Don’t describe the method used 57
Don’t name or provide details about the site/location 58
Don’t use language/content which sensationalizes, romanticizes or normalizes suicide, or that presents it as a viable solution to problems 58
Don’t oversimplify the reason for a suicide or reduce it to a single factor 59
Don’t use sensational language in headlines 59
Don’t use photographs, video footage, audio recordings, digital or social media links 60
Don’t report the details of a suicide note..60

PROSPERO

The protocol was preregistered with PROSPERO and the registration number is CRD42023467588. We have made some changes to the protocol, none of which affected the studies included in the systematic review and meta-analysis.

- We initially planned to explore a secondary research question ("What factors might explain heterogeneity between studies?") but did not pursue it due to insufficient studies.
- We originally planned to use the Robins-I tool. However, since all included studies were uncontrolled pre-post designs (lacking control groups and unlikely to be rated as low or moderate risk), we opted not to conduct a formal risk-of-bias assessment.
- We initially planned subgroup (exploratory) analyses (analysing high-quality studies separately and exploring heterogeneity by region, decade, implementation strength, and suicide subgroups). However, we did not conduct these analyses because the available data were insufficient for meaningful comparisons.

Detailed search strategy

Electronic database searches

We searched PubMed (including MEDLINE), Scopus, Embase (Ovid), PsycINFO (Ovid), and Web of Science from database inception to 10 February 2025 (with a search update conducted on 17 November 2025). Searches targeted titles, abstracts, and keywords using the following strategy applied across databases: ((suicid* OR self-harm) AND (media OR newspaper* OR print OR press OR television* OR radio* OR internet*) AND (report* OR cover* OR portray*) AND (guide* OR recommend* OR advice* OR advise* OR prevent*)). No language restrictions were applied.

Google Scholar search

To capture grey literature, we conducted an additional search in Google Scholar using the query "suicide AND media" on 10 February 2025 (with a search update conducted on 17 November 2025). All available result pages were screened in full. Records were exported manually and imported into Covidence for management and screening. No language restrictions were applied.

Searching other resources

To identify additional literature, we contacted members of the International Association for Suicide Prevention's (IASP) *Suicide and the Media* Special Interest Group. We also screened the reference lists of identified reviews and all included studies. Finally, we conducted a cited-reference search for each included study in Scopus, using Web of Science when a record was not indexed in Scopus.

Non-English records

For records in languages other than English identified through any source, eligibility assessment and data extraction were conducted using Google Translate and, where possible, verified with fluent speakers.

eTable 1. Full list of each individual recommendation from the guidelines used in any given study, mapped against the recommendations in the WHO/IASP 2023 guidelines

Study	Guideline recommendation, as shown in the study[a,b]	WHO/IASP (2023) recommendations	Duplicate WHO/IASP recommendation within study[c]
Kim 2005	Following effect (e.g., impact on bereavement, subsequent effect after attempt)	Do educate the public with the facts about suicide and suicide prevention based on accurate information.	Yes
Kim 2005	Urging appropriate solutions	Do educate the public with the facts about suicide and suicide prevention based on accurate information.	Yes
Kim 2005	Information from reliable, verified source	Do educate the public with the facts about suicide and suicide prevention based on accurate information.	Yes
Kim 2005	[REVERSE CODED] Headlines: Inclusion of the word "suicide"	Don't use sensational language in headlines.	Yes
Kim 2005	[REVERSE CODED] Headlines: Sensationalisation	Don't use sensational language in headlines.	Yes
Kim 2005	[REVERSE CODED] Contents: Description of suicide methods	Don't describe the method used.	No
Kim 2005	[REVERSE CODED] Contents: Description of suicide place	Don't name or provide details about the site/location.	Yes
Kim 2005	[REVERSE CODED] Contents: Description of scene	Don't name or provide details about the site/location.	Yes
Kim 2005	[REVERSE CODED] Contents: Wills "i.e., suicide notes or wills in the content of news articles"	Don't report the details of suicide notes.	No
Kim 2005	[REVERSE CODED] Attributing reasons for suicide to simply single or only a few reasons	Don't oversimplify the reason for a suicide or reduce it to a single factor.	No
Kim 2005	[REVERSE CODED] Romanticisation	Don't use language/content which sensationalises, romanticises, or normalises suicide, or that presents it as a viable solution to problems.	Yes
Kim 2005	[REVERSE CODED] Suicide as a solution	Don't use language/content which sensationalises, romanticises, or normalises suicide, or that presents it as a viable solution to problems.	Yes
Ju 2024	Containing information about negative consequences	Do educate the public with the facts about suicide and suicide prevention based on accurate information.	Yes
Ju 2024	Containing educational contents	Do educate the public with the facts about suicide and suicide prevention based on accurate information.	Yes
Ju 2024	Containing coping information	Do report stories of how to cope with life stressors and/or suicidal thoughts and the importance of help-seeking.	No
Ju 2024	Headline: Not containing the term "suicide"	Don't use sensational language in headlines.	Yes
Ju 2024	Headline: Not containing information on cause method	Don't use sensational language in headlines.	Yes
Ju 2024	Headline: Not containing information on location	Don't use sensational language in headlines.	Yes
Ju 2024	Headline: Not containing information on cause	Don't use sensational language in headlines.	Yes
Ju 2024	Headline: Not containing positive contextualization	Don't use sensational language in headlines.	Yes
Ju 2024	Text body: Not containing information on cause method	Don't describe the method used.	No
Ju 2024	Text body: Not containing information on location	Don't name or provide details about the site/location.	No
Ju 2024	Text body: Not containing information on cause (Note. as per table 1, "cause" refers to "Reason(s) for suicide provided with a connecting word such as 'after,' 'due to,' 'as,' 'because,' 'under' etc.")	Don't oversimplify the reason for a suicide or reduce it to a single factor.	No

Ju 2024	Not containing photos	Don’t use photographs, video footage, audio recordings, or digital or social media links.	No
Ju 2024	Text body: Not containing the term 'suicide'	Don’t use language/content which sensationalises, romanticises, or normalises suicide, or that presents it as a viable solution to problems.	Yes
Ju 2024	Not containing positive contextualization	Don’t use language/content which sensationalises, romanticises, or normalises suicide, or that presents it as a viable solution to problems.	Yes
Sinyor 2024	Community resources	Do provide accurate information about where and how to seek help for suicidal thoughts and suicidal crises.	No
Sinyor 2024	[REVERSE CODED] Identifies deceased as a celebrity	Do apply particular caution when reporting celebrity suicides.	No
Sinyor 2024	Alternatives to suicide	Do educate the public with the facts about suicide and suicide prevention based on accurate information.	Yes
Sinyor 2024	Warning signs of suicidal behaviour	Do educate the public with the facts about suicide and suicide prevention based on accurate information.	Yes
Sinyor 2024	How to approach someone	Do educate the public with the facts about suicide and suicide prevention based on accurate information.	Yes
Sinyor 2024	Positive outcome of a suicide-related crisis	Do report stories of how to cope with life stressors and/or suicidal thoughts and the importance of help-seeking.	Yes
Sinyor 2024	Message of hope	Do report stories of how to cope with life stressors and/or suicidal thoughts and the importance of help-seeking.	Yes
Sinyor 2024	[REVERSE CODED] Interview with the bereaved (Note. Classified as "Putatively harmful" in the study)	Do apply caution when interviewing bereaved family members or friends or persons with lived experience of suicide.	No
Sinyor 2024	[REVERSE CODED] Word “suicide” in the headline	Don’t use sensational language in headlines.	Yes
Sinyor 2024	[REVERSE CODED] Suicide method (in headline)	Don’t use sensational language in headlines.	Yes
Sinyor 2024	[REVERSE CODED] Suicide method (text)	Don’t describe the method used.	Yes
Sinyor 2024	[REVERSE CODED] Method described in detail	Don’t describe the method used.	Yes
Sinyor 2024	[REVERSE CODED] Reasons for suicide (simplistic)	Don’t oversimplify the reason for a suicide or reduce it to a single factor.	No
Sinyor 2024	[REVERSE CODED] Photo (deceased)	Don’t use photographs, video footage, audio recordings, or digital or social media links.	Yes
Sinyor 2024	[REVERSE CODED] Photo (of someone looking sad)	Don’t use photographs, video footage, audio recordings, or digital or social media links.	Yes
Sinyor 2024	[REVERSE CODED] Favourable characteristic (deceased) (Note. Considered "Putatively harmful" in the study; see "Favorable and unfavorable characteristics refer to statements in an article expressing positive or negative value judgments, respectively, about a person’s character, personality or behavior")	Don’t use language/content which sensationalises, romanticises, or normalises suicide, or that presents it as a viable solution to problems.	Yes
Sinyor 2024	[REVERSE CODED] Statement that suicide is inevitable	Don’t use language/content which sensationalises, romanticises, or normalises suicide, or that presents it as a viable solution to problems.	Yes
Sinyor 2024	[REVERSE CODED] Glorified or romanticized suicide	Don’t use language/content which sensationalises, romanticises, or normalises suicide, or that presents it as a viable solution to problems.	Yes
Sinyor 2024	[REVERSE CODED] Sensationalistic reporting	Don’t use language/content which sensationalises, romanticises, or normalises suicide, or that presents it as a viable solution to problems.	Yes

Acosta 2020	WTD3: Support resources (Note. Full item in the appendix of the study: "Provide information about support resources and crisis helplines")	Do provide accurate information about where and how to seek help for suicidal thoughts and suicidal crises.	No
Acosta 2020	WTD2: Alternatives to suicide (Note. Full item in the appendix of the study: "Highlight alternatives to suicide either through general information or through stories of people illustrating how to cope with adverse circumstances or suicidal thoughts and how to get help")	Do educate the public with the facts about suicide and suicide prevention based on accurate information.	Yes
Acosta 2020	WTD4: Risk factors and warning signs. (Note. Full item in the appendix of the study: "Provide information about risk factors and warning signs")	Do educate the public with the facts about suicide and suicide prevention based on accurate information.	Yes
Acosta 2020	WTD5. Depression often associated. (Note. Full item in the appendix of the study: "Convey the message that depression is often associated with suicidal behavior and that depression is a treatable condition")	Do educate the public with the facts about suicide and suicide prevention based on accurate information.	Yes
Acosta 2020	WTD7: Educate the public (Note. Full item in the appendix of the study: "Educate the public about the facts of suicide and suicide prevention without spreading myths")	Do educate the public with the facts about suicide and suicide prevention based on accurate information.	Yes
Acosta 2020	WTA2: Headlines (Note. Full item in the appendix of the study: "Avoid headlines containing the word suicide or specifying the method or location")	Don't use sensational language in headlines.	No
Acosta 2020	WTA6: Detailed account of method (Note. Full item in the appendix of the study: "Avoid providing detailed information or an explicit description of the method used of a completed or attempted suicide")	Don't describe the method used.	No
Acosta 2020	WTA7: Detailed account of location (Note. Full item in the appendix of the study: "Avoid providing detailed information about the location of a completed or attempted suicide")	Don't name or provide details about the site/location.	No
Acosta 2020	WTA4: Suicide notes (Note. Full item in the appendix of the study: "Do not publish suicide notes in any of their forms (paper, final text messages, social media posts, or emails")	Don't report the details of suicide notes.	No
Acosta 2020	WTA11: Simplistic reasons (Note. Full item in the appendix of the study: "Do not provide simplistic reasons for why the suicide occurred")	Don't oversimplify the reason for a suicide or reduce it to a single factor.	No
Acosta 2020	WTA3: Pictures or video (Note. Full item in the appendix of the study: "Be cautious with the use of pictures or video footage. Avoid pictures, video of the deceased, method used or of the scene, or of shocking contents (i.e. person on a ledge or similar or elements used) and of links to social networks that contain them")	Don't use photographs, video footage, audio recordings, or digital or social media links.	No
Acosta 2020	WTA1: Sensationalism (Note. Full item in the appendix of the study: "Do not sensationalize suicide"	Don't use language/content which sensationalises, romanticises, or normalises suicide, or that presents it as a viable solution to problems.	Yes
Acosta 2020	WTA8: Glorification (Note. Full item in the appendix of the study: "Do not glorify the person who has committed suicide")	Don't use language/content which sensationalises, romanticises, or normalises suicide, or that presents it as a viable solution to problems.	Yes
Acosta 2020	WTA9: Normalization (Note. Full item in the appendix of the study: "Do not use language that normalizes suicide")	Don't use language/content which sensationalises, romanticises, or normalises suicide, or that presents it as a viable solution to problems.	Yes
Acosta 2020	WTA10: Suicide as a solution (Note. Full item in the appendix of the study: "Do not depict suicide as a solution to problems or as a means of coping with personal problems")	Don't use language/content which sensationalises, romanticises, or normalises suicide, or that presents it as a viable solution to problems.	Yes
Acosta 2020	WTA13: Understandable response (Note. Full item in the appendix of the study: "Avoid reporting suicidal behavior as an Understandable response to social or cultural changes or degradation")	Don't use language/content which sensationalises, romanticises, or normalises suicide, or that presents it as a viable solution to problems.	Yes

Acosta 2020	WTA14: Blame (Note. Full item in the appendix of the study: "Do not apportion blame")	Don’t use language/content which sensationalises, romanticises, or normalises suicide, or that presents it as a viable solution to problems.	Yes
Acosta 2020	WTD1: Completed suicide (Note. Full item in the appendix of the study: "Refer to suicide as a completed suicide not as a successful suicide")	Don’t use language/content which sensationalises, romanticises, or normalises suicide, or that presents it as a viable solution to problems.	Yes
Acosta 2020	WTA5: Visibility of news article (Note. Full item in the appendix of the study: "Do not place suicide news articles prominently. Newspaper stories about suicide should be located on the inside pages")	Don’t position suicide-related content as the top story and don’t unduly repeat such stories.	Yes
Acosta 2020	WTA15: Repetition (Note. Full item in the appendix of the study: "Do not unduly repeat suicide news articles")	Don’t position suicide-related content as the top story and don’t unduly repeat such stories.	Yes
Jamieson 2003	Depression mentioned	Do educate the public with the facts about suicide and suicide prevention based on accurate information.	No
Jamieson 2003	[REVERSE CODED] Suicide implied in headline	Don’t use sensational language in headlines.	No
Jamieson 2003	[REVERSE CODED] Cause suggested	Don’t oversimplify the reason for a suicide or reduce it to a single factor.	No
Jamieson 2003	[REVERSE CODED] First 9 pages	Don’t position suicide-related content as the top story and don’t unduly repeat such stories.	No
Fu 2008	Containing positive message(s)	Do report stories of how to cope with life stressors and/or suicidal thoughts and the importance of help-seeking.	No
Fu 2008	[REVERSE CODED] Mentioning suicide method in the headline	Don’t use sensational language in headlines.	Yes
Fu 2008	[REVERSE CODED] Mentioning problem encountered in the headline	Don’t use sensational language in headlines.	Yes
Fu 2008	[REVERSE CODED] Accompanied with photograph(s)	Don’t use photographs, video footage, audio recordings, or digital or social media links.	Yes
Fu 2008	[REVERSE CODED] Accompanied with graphical presentations	Don’t use photographs, video footage, audio recordings, or digital or social media links.	Yes
Fu 2008	[REVERSE CODED] Front-page placement	Don’t position suicide-related content as the top story and don’t unduly repeat such stories.	No
Pirkis 2009	Does the item provide information on help services? (YES)	Do provide accurate information about where and how to seek help for suicidal thoughts and suicidal crises.	No
Pirkis 2009	Is there reference to the fact that the person who died by suicide was a celebrity (NO)	Do apply particular caution when reporting celebrity suicides.	No
Pirkis 2009	Is suicide portrayed as "merely a social phenomenon" as opposed to being related to mental disorder (NO)	Do educate the public with the facts about suicide and suicide prevention based on accurate information.	No
Pirkis 2009	Are the bereaved interviewed? (NO)	Do apply caution when interviewing bereaved family members or friends or persons with lived experience of suicide.	No
Pirkis 2009	Is the word "suicide" used in the headline (NO)	Don’t use sensational language in headlines.	No
Pirkis 2009	Is there a detailed discussion of the method used (NO)	Don’t describe the method used.	No
Pirkis 2009	Is a photograph, a diagram or footage depicting the suicide scene, precise location or method used with the item (NO)	Don’t use photographs, video footage, audio recordings, or digital or social media links.	No
Pirkis 2009	Does the item have any examples of inappropriate language? (NO)	Don’t use language/content which sensationalises, romanticises, or normalises suicide, or that presents it as a viable solution to problems.	No
Pirkis 2009	Is the item inappropriately located (NO)	Don’t position suicide-related content as the top story and don’t unduly repeat such stories.	No

Ramadas 2011	Attempts to make the readers realize that the act was avoidable	Do educate the public with the facts about suicide and suicide prevention based on accurate information.	Yes
Ramadas 2011	Acknowledge the presence of physical and mental illness in the deceased	Do educate the public with the facts about suicide and suicide prevention based on accurate information.	Yes
Ramadas 2011	Consider the possible impact on survivors and families in terms of psychological suffering and stigma	Do educate the public with the facts about suicide and suicide prevention based on accurate information.	Yes
Ramadas 2011	Utilize the report as an opportunity for public education, to highlight possible and established suicide counters	Do educate the public with the facts about suicide and suicide prevention based on accurate information.	Yes
Ramadas 2011	Avoid details of method and how the method was procured	Don’t describe the method used.	Yes
Ramadas 2011	Avoid reporting the identity of the deceased, details of the method, and the scene	Don’t describe the method used.	Yes
Ramadas 2011	Avoid reporting the identity of the deceased, details of the method, and the scene	Don’t name or provide details about the site/location.	No
Ramadas 2011	Avoid overstatement	Don’t oversimplify the reason for a suicide or reduce it to a single factor.	No
Ramadas 2011	Avoid photographs of the deceased, the methods, and the scene	Don’t use photographs, video footage, audio recordings, or digital or social media links.	No
Ramadas 2011	Avoid sensational coverage	Don’t use language/content which sensationalises, romanticises, or normalises suicide, or that presents it as a viable solution to problems.	Yes
Ramadas 2011	Avoid correlating suicide with a cause that is common and applicable to any reader	Don’t use language/content which sensationalises, romanticises, or normalises suicide, or that presents it as a viable solution to problems.	Yes
Ramadas 2011	Avoid reporting suicide as the only method of coping in the situation of personal crisis	Don’t use language/content which sensationalises, romanticises, or normalises suicide, or that presents it as a viable solution to problems.	Yes
Ramadas 2011	Avoid reporting as an understandable response to social and cultural changes or degradation	Don’t use language/content which sensationalises, romanticises, or normalises suicide, or that presents it as a viable solution to problems.	Yes
Ramadas 2011	Do not glorify victims as martyrs or the method as an acceptable one	Don’t use language/content which sensationalises, romanticises, or normalises suicide, or that presents it as a viable solution to problems.	Yes
Ramadas 2011	Avoid front page reporting, reporting in boxes, and enlarged headlines	Don’t position suicide-related content as the top story and don’t unduly repeat such stories.	No
Roškar 2017	Stating suicide-prevention resources (e. g., emergency line phone numbers)	Do provide accurate information about where and how to seek help for suicidal thoughts and suicidal crises.	Yes
Roškar 2017	General information about suicide-prevention resources	Do provide accurate information about where and how to seek help for suicidal thoughts and suicidal crises.	Yes
Roškar 2017	Careful writing about VIP suicides	Do apply particular caution when reporting celebrity suicides.	No
Roškar 2017	Informing the public about the reasons for suicide	Do educate the public with the facts about suicide and suicide prevention based on accurate information.	No
Roškar 2017	Real-life stories of those who overcame their hardships	Do report stories of how to cope with life stressors and/or suicidal thoughts and the importance of help-seeking.	No
Roškar 2017	[REVERSE CODED] Headlines	Don’t use sensational language in headlines.	No
Roškar 2017	[REVERSE CODED] Detailed descriptions of a suicide method	Don’t describe the method used.	No
Roškar 2017	[REVERSE CODED] Detailed descriptions of a suicide location	Don’t name or provide details about the site/location.	No

Roškar 2017	[REVERSE CODED] Inappropriate photographic material	Don't use photographs, video footage, audio recordings, or digital or social media links.	No
Roškar 2017	Non-sensationalistic writing style	Don't use language/content which sensationalises, romanticises, or normalises suicide, or that presents it as a viable solution to problems.	No
Garrido-Fabián 2018	Reference to mental problems. (Note. Full item text is under section 2.3: "1) The sensationalistic covering of suicides should be avoided in a diligent manner, particularly when it involves a celebrity. This covering should be minimized as much as possible. Likewise, any mental problem that the celebrity could have should be recognized. Efforts to avoid exaggerations should be made. Pictures of the victim, of the method used, and the scene of the suicide should be avoided. The cover headlines are never the ideal place to inform about a suicide")	Do apply particular caution when reporting celebrity suicides.	No
Garrido-Fabián 2018	The impact on families is mentioned. (Note. Full item text is under section 2.3: "5) Reports should consider the impact on family and other survivors in terms of stigma and psychological suffering")	Do educate the public with the facts about suicide and suicide prevention based on accurate information.	No
Garrido-Fabián 2018	Avoid descriptions about the method used and notes. (Note. Full item text is under section 2.3: "2) Detailed descriptions about the method used and how the victim had access to it should be avoided. Research has demonstrated that media coverage has a greater impact on the suicide method used than on the frequency of suicides. Certain settings – bridges, cliffs, high buildings, railways, etc. – are traditionally associated with suicide, and the extra advertisement increases the risk that more people will use them")	Don't describe the method used.	No
Garrido-Fabián 2018	Avoid descriptions about the method used and notes. (Note. Full item text is under section 2.3: "2) Detailed descriptions about the method used and how the victim had access to it should be avoided. Research has demonstrated that media coverage has a greater impact on the suicide method used than on the frequency of suicides. Certain settings – bridges, cliffs, high buildings, railways, etc. – are traditionally associated with suicide, and the extra advertisement increases the risk that more people will use them")	Don't report the details of suicide notes.	No
Garrido-Fabián 2018	Not presented as unexplainable or simplistic. (Note. Full item text is under section 2.3: "3) The suicide should not be informed as something unexplainable or simplistic. Suicide is never the result of a single factor or fact. It is usually caused by a complex interaction of many factors such as mental and physical health, substance abuse, family and interpersonal conflicts, and stressful events. It is useful to recognize that a variety of factors contribute to suicide")	Don't oversimplify the reason for a suicide or reduce it to a single factor.	No
Garrido-Fabián 2018	Not described as method to face problems. (Note. Full item text is under section 2.3: "4) Suicide shouldn't be described as a method to face personal problems such as bankruptcy, inability to pass a test, or sexual abuse")	Don't use language/content which sensationalises, romanticises, or normalises suicide, or that presents it as a viable solution to problems.	Yes
Garrido-Fabián 2018	Do not glorify victims as martyrs. (Note. Full item text is under section 2.3: "6) To glorify suicide victims as martyrs and objects of public flattering may suggest to vulnerable people that society honors suicide behavior. Instead, there should be an emphasis on regretting the death of the person")	Don't use language/content which sensationalises, romanticises, or normalises suicide, or that presents it as a viable solution to problems.	Yes
Sinyor 2021	Community resources	Do provide accurate information about where and how to seek help for suicidal thoughts and suicidal crises.	No

Sinyor 2021	[REVERSE CODED] Identifies deceased as a celebrity	Do apply particular caution when reporting celebrity suicides.	No
Sinyor 2021	Alternatives to suicide	Do educate the public with the facts about suicide and suicide prevention based on accurate information.	Yes
Sinyor 2021	Warning signs of suicidal behavior	Do educate the public with the facts about suicide and suicide prevention based on accurate information.	Yes
Sinyor 2021	How to approach someone	Do educate the public with the facts about suicide and suicide prevention based on accurate information.	Yes
Sinyor 2021	Positive outcome of a suicidal crisis	Do report stories of how to cope with life stressors and/or suicidal thoughts and the importance of help-seeking.	Yes
Sinyor 2021	Message of hope	Do report stories of how to cope with life stressors and/or suicidal thoughts and the importance of help-seeking.	Yes
Sinyor 2021	[REVERSE CODED] Interview with the bereaved. Note. Item classified as "Putatively harmful" in the study.	Do apply caution when interviewing bereaved family members or friends or persons with lived experience of suicide.	No
Sinyor 2021	[REVERSE CODED] Word "suicide" in the headline	Don't use sensational language in headlines.	Yes
Sinyor 2021	[REVERSE CODED] Suicide method (headline)	Don't use sensational language in headlines.	Yes
Sinyor 2021	[REVERSE CODED] Suicide method (text)	Don't describe the method used.	Yes
Sinyor 2021	[REVERSE CODED] Method described in detail	Don't describe the method used.	Yes
Sinyor 2021	[REVERSE CODED] Monocausal explanation for suicide	Don't oversimplify the reason for a suicide or reduce it to a single factor.	No
Sinyor 2021	[REVERSE CODED] Photo (deceased)	Don't use photographs, video footage, audio recordings, or digital or social media links.	Yes
Sinyor 2021	[REVERSE CODED] Photo (of someone looking sad)	Don't use photographs, video footage, audio recordings, or digital or social media links.	Yes
Sinyor 2021	[REVERSE CODED] Favourable characteristic (deceased). Note. Item classified as "Putatively harmful" in the paper; see footnote in Table 1: "Favorable and unfavorable characteristics refer to statements in an article expressing positive or negative value judgments, respectively, about a person's character, personality or behavior"	Don't use language/content which sensationalises, romanticises, or normalises suicide, or that presents it as a viable solution to problems.	Yes
Sinyor 2021	[REVERSE CODED] Statement that suicide is inevitable	Don't use language/content which sensationalises, romanticises, or normalises suicide, or that presents it as a viable solution to problems.	Yes
Sinyor 2021	[REVERSE CODED] Exciting reporting	Don't use language/content which sensationalises, romanticises, or normalises suicide, or that presents it as a viable solution to problems.	Yes
Sinyor 2021	[REVERSE CODED] Glorified/romanticized suicide	Don't use language/content which sensationalises, romanticises, or normalises suicide, or that presents it as a viable solution to problems.	Yes
Sinyor 2021	[REVERSE CODED] Statement of approval of suicide	Don't use language/content which sensationalises, romanticises, or normalises suicide, or that presents it as a viable solution to problems.	Yes
Armstrong 2025	Mentions a suicide prevention programme/support service	Do provide accurate information about where and how to seek help for suicidal thoughts and suicidal crises.	Yes
Armstrong 2025	Provides contact details for a suicide support service	Do provide accurate information about where and how to seek help for suicidal thoughts and suicidal crises.	Yes
Armstrong 2025	Recognises link with poor mental health	Do educate the public with the facts about suicide and suicide prevention based on accurate information.	Yes

Armstrong 2025	Recognises link with substance dependence/use	Do educate the public with the facts about suicide and suicide prevention based on accurate information.	Yes
Armstrong 2025	Dispels the myths that there are no preceding warning signs and/or that there is nothing you can do to prevent suicide	Do educate the public with the facts about suicide and suicide prevention based on accurate information.	Yes
Armstrong 2025	Expert opinion from a mental health professional	Do educate the public with the facts about suicide and suicide prevention based on accurate information.	Yes
Armstrong 2025	[REVERSE CODED] Interview with bereaved persons	Do apply caution when interviewing bereaved family members or friends or persons with lived experience of suicide.	No
Armstrong 2025	[REVERSE CODED] “Suicide” in headline	Don’t use sensational language in headlines.	Yes
Armstrong 2025	[REVERSE CODED] Suicide method in headline	Don’t use sensational language in headlines.	Yes
Armstrong 2025	[REVERSE CODED] Life event(s) in headline	Don’t use sensational language in headlines.	Yes
Armstrong 2025	[REVERSE CODED] Suicide method reported	Don’t describe the method used.	Yes
Armstrong 2025	[REVERSE CODED] Detailed account of method (ie, at least two specific details about how the method was implemented)	Don’t describe the method used.	Yes
Armstrong 2025	[REVERSE CODED] Public site named as location of a suicide death/attempt	Don’t name or provide details about the site/location.	No
Armstrong 2025	[REVERSE CODED] Details from suicide note reported	Don’t report the details of suicide notes.	No
Armstrong 2025	[REVERSE CODED] Monocausal explanation for suicidality	Don’t oversimplify the reason for a suicide or reduce it to a single factor.	Yes
Armstrong 2025	[REVERSE CODED] Negative life event(s) related to suicide reported	Don’t oversimplify the reason for a suicide or reduce it to a single factor.	Yes
Armstrong 2025	[REVERSE CODED] Any accompanying photo	Don’t use photographs, video footage, audio recordings, or digital or social media links.	Yes
Armstrong 2025	[REVERSE CODED] Photo of a suicidal person	Don’t use photographs, video footage, audio recordings, or digital or social media links.	Yes
Armstrong 2025	[REVERSE CODED] Article on front page	Don’t position suicide-related content as the top story and don’t unduly repeat such stories.	Yes
Armstrong 2025	[REVERSE CODED] Article in first three pages	Don’t position suicide-related content as the top story and don’t unduly repeat such stories.	Yes
Eriyanto 2025	The article mentions suicide prevention programs or support services	Do provide accurate information about where and how to seek help for suicidal thoughts and suicidal crises.	Yes
Eriyanto 2025	The article provides contact details for support services	Do provide accurate information about where and how to seek help for suicidal thoughts and suicidal crises.	Yes
Eriyanto 2025	The article acknowledges the link between suicide and mental disorders	Do educate the public with the facts about suicide and suicide prevention based on accurate information.	Yes
Eriyanto 2025	The article acknowledges the link between suicide and substance abuse/addictive substances (e.g., drugs or alcohol)	Do educate the public with the facts about suicide and suicide prevention based on accurate information.	Yes
Eriyanto 2025	The article reports actions taken by the victim prior to suicide (e.g., visiting a doctor, undergoing treatment)	Do educate the public with the facts about suicide and suicide prevention based on accurate information.	Yes

Eriyanto 2025	The article refutes myths suggesting there are no warning signs before suicide or that nothing can be done to prevent it	Do educate the public with the facts about suicide and suicide prevention based on accurate information.	Yes
Eriyanto 2025	Suicide is explained as resulting from multiple contributing factors (multifactorial causes)	Do educate the public with the facts about suicide and suicide prevention based on accurate information.	Yes
Eriyanto 2025	The article cites research findings on suicide and its prevention	Do educate the public with the facts about suicide and suicide prevention based on accurate information.	Yes
Eriyanto 2025	Expert opinions are provided (e.g., from psychology, mental health, or religion)	Do educate the public with the facts about suicide and suicide prevention based on accurate information.	Yes
Eriyanto 2025	The article includes stories or accounts of individuals who successfully overcame suicidal thoughts or attempts	Do report stories of how to cope with life stressors and/or suicidal thoughts and the importance of help-seeking.	Yes
Eriyanto 2025	The article presents information about coping with depression and importance of seeking help	Do report stories of how to cope with life stressors and/or suicidal thoughts and the importance of help-seeking.	Yes
Eriyanto 2025	[REVERSE CODED] Interviews with bereaved relatives	Do apply caution when interviewing bereaved family members or friends or persons with lived experience of suicide.	Yes
Eriyanto 2025	[REVERSE CODED] Coverage of impact on bereaved	Do apply caution when interviewing bereaved family members or friends or persons with lived experience of suicide.	Yes
Eriyanto 2025	[REVERSE CODED] The word “suicide” appears in the headline	Don’t use sensational language in headlines.	Yes
Eriyanto 2025	[REVERSE CODED] Suicide method mentioned in the headline	Don’t use sensational language in headlines.	Yes
Eriyanto 2025	[REVERSE CODED] Victim’s identity mentioned in the headline	Don’t use sensational language in headlines.	Yes
Eriyanto 2025	[REVERSE CODED] Negative life event mentioned in the headline	Don’t use sensational language in headlines.	Yes
Eriyanto 2025	[REVERSE CODED] Mentioning suicide method	Don’t describe the method used.	No
Eriyanto 2025	[REVERSE CODED] Mentioning specific location of death	Don’t name or provide details about the site/location.	No
Eriyanto 2025	[REVERSE CODED] Quoting from victim’s note	Don’t report the details of suicide notes.	No
Eriyanto 2025	[REVERSE CODED] Suicide attributed to a single cause	Don’t oversimplify the reason for a suicide or reduce it to a single factor.	Yes
Eriyanto 2025	[REVERSE CODED] Oversimplified reasons	Don’t oversimplify the reason for a suicide or reduce it to a single factor.	Yes
Eriyanto 2025	[REVERSE CODED] Article accompanied by a photo	Don’t use photographs, video footage, audio recordings, or digital or social media links.	Yes
Eriyanto 2025	[REVERSE CODED] Victim’s personal photo	Don’t use photographs, video footage, audio recordings, or digital or social media links.	Yes
Eriyanto 2025	[REVERSE CODED] Photo of the body	Don’t use photographs, video footage, audio recordings, or digital or social media links.	Yes
Eriyanto 2025	[REVERSE CODED] Photo of suicide location	Don’t use photographs, video footage, audio recordings, or digital or social media links.	Yes
Eriyanto 2025	[REVERSE CODED] Photo of suicide method	Don’t use photographs, video footage, audio recordings, or digital or social media links.	Yes
Eriyanto 2025	[REVERSE CODED] Illustration photo	Don’t use photographs, video footage, audio recordings, or digital or social media links.	Yes
Eriyanto 2025	[REVERSE CODED] Photo of doctors/police/observers	Don’t use photographs, video footage, audio recordings, or digital or social media links.	Yes
Eriyanto 2025	[REVERSE CODED] Posting suicidal indications on social media	Don’t use photographs, video footage, audio recordings, or digital or social media links.	Yes

Eriyanto 2025	[REVERSE CODED] Suicide portrayed as a solution	Don’t use language/content which sensationalises, romanticises, or normalises suicide, or that presents it as a viable solution to problems.	No
Shaw 2025	Contact support service	Do provide accurate information about where and how to seek help for suicidal thoughts and suicidal crises.	No
Shaw 2025	Debunking false myths	Do educate the public with the facts about suicide and suicide prevention based on accurate information.	Yes
Shaw 2025	Alternatives to suicide	Do educate the public with the facts about suicide and suicide prevention based on accurate information.	Yes
Shaw 2025	Warning signs	Do educate the public with the facts about suicide and suicide prevention based on accurate information.	Yes
Shaw 2025	Hopeful stories	Do report stories of how to cope with life stressors and/or suicidal thoughts and the importance of help-seeking.	No
Shaw 2025	[REVERSE CODED] Headline about suicide	Don’t use sensational language in headlines.	Yes
Shaw 2025	[REVERSE CODED] Headline about suicide method	Don’t use sensational language in headlines.	Yes
Shaw 2025	[REVERSE CODED] Suicide method reported	Don’t describe the method used.	No
Shaw 2025	[REVERSE CODED] Citation from suicide note	Don’t report the details of suicide notes.	No
Shaw 2025	[REVERSE CODED] Visual content	Don’t use photographs, video footage, audio recordings, or digital or social media links.	No
Shaw 2025	[REVERSE CODED] Glorification of suicide	Don’t use language/content which sensationalises, romanticises, or normalises suicide, or that presents it as a viable solution to problems.	Yes
Shaw 2025	[REVERSE CODED] Stigmatising language	Don’t use language/content which sensationalises, romanticises, or normalises suicide, or that presents it as a viable solution to problems.	Yes
Shaw 2025	[REVERSE CODED] Suggesting a suicide epidemic	Don’t use language/content which sensationalises, romanticises, or normalises suicide, or that presents it as a viable solution to problems.	Yes
Shaw 2025	[REVERSE CODED] Suggesting inevitability	Don’t use language/content which sensationalises, romanticises, or normalises suicide, or that presents it as a viable solution to problems.	Yes
Shaw 2025	[REVERSE CODED] Spreading false myths	Don’t use language/content which sensationalises, romanticises, or normalises suicide, or that presents it as a viable solution to problems.	Yes

[a]Reverse coded items include “[REVERSE CODED]” prior to the text.
[b]Items that did not fall under any of the WHO/IASP (2023) recommendations were not mapped; examples include: "Emotional empathy to suicide" (Kim 2005); "Unfavourable characteristic (deceased)" (Sinyor 2021; 2024); "Murder-suicide"; "Assisted suicide" (Jamieson 2003); "Be neutral in reporting" (Ramadas 2011); "Displays of empathy towards the grieving" (Roškar 2017).
[c]'Yes' indicates that multiple items within the same study mapped to the same WHO/IASP recommendation (i.e., a duplicate WHO category). In these cases, one item was randomly selected for inclusion, as described in the Methods section of the manuscript.

Studies excluded after full-text screening

A total of 41 papers were screened in full, and 26 studies were subsequently excluded for specific reasons as stated in the PRISMA flow diagram.

Seven studies were excluded because they did not measure the outcomes of interest prior to the guidelines release.[1-8] For example, a Korean study investigating media adherence to suicide reporting guidelines in newspapers,[2] and a U.S. study on suicide coverage in newspapers following the publication of media guidelines,[5] only included post-guidelines outcome data.

Another six studies were excluded because they were unrelated to media guidelines for reporting suicide.[9-14] Examples include government appeals issued without the release of media guidelines[14] and national suicide prevention programs not specifically designed as guidelines.[12]

Three studies met the inclusion criteria but were excluded because we could not determine the pre- and post-guidelines timepoints, and we did not receive the requested data from the authors.[15-17] Specifically, in two studies, the figures in the published papers did not clearly indicate which time points were pre- versus post-guidelines introduction,[15] or the data were presented only in aggregated formats.[17] Another study only reported significant values for the outcomes.[16]

Two studies focused on implementation activities related to media guidelines, rather than introducing any guidelines.[18,19] For example, a U.S. study described the "Know the Signs" campaign, which aimed to increase adherence to existing suicide reporting guidelines through media outreach and journalist education.[18] Similarly, a German study implemented web-based training to promote responsible suicide reporting, focusing on reducing Werther effects and encouraging Papageno effects.[19]

Two otherwise relevant studies were excluded because their data were included in a more recent study.[20,21] Both studies focused on media reporting of Viennese subway suicides; however, in line with our criterion of using the most recent data, we opted to include a later study that analysed the same dataset and included subway suicides as part of the general population suicides (see Niederkrotenthaler and Sonneck, 2007).

One study was excluded because it was unclear which guidelines were used. The outcome was measured more than twice, and the version or year of the guidelines was unclear; thus, pre- and post-guidelines timepoints could not be determined.[22]

One study was excluded due to the wrong study design. It compared media reporting in Spain versus Victoria, Australia, and did not have pre- and post-guidelines outcome data.[23]

Another study was excluded because it focused solely on student suicides, while our interest lay in suicides among the general population.[24]

One study did not include primary or secondary data and was therefore excluded.[25]

Finally, one study was excluded because it investigated the wrong outcome: the intervention addressed awareness and knowledge of suicide reporting among Malaysian media professionals, rather than actual reporting practices.[26]

1. Chiang YC, Chung FY, Lee CY, Shih HL, Lin DC, Lee MB. Suicide Reporting on Front Pages of Major Newspapers in Taiwan Violating Reporting Recommendations Between 2001 and 2012. *Health Communication*. 2016;31(11):1395-1404. doi:10.1080/10410236.2015.1074024
2. Chun J, Kim J, Lee S. Fidelity assessment of the suicide reporting guidelines in Korean newspapers. *BMC Public Health*. 2018;18(1)doi:10.1186/s12889-018-6014-4
3. McTernan N, Spillane A, Cully G, Cusack E, O'Reilly T, Arensman E. Media reporting of suicide and adherence to media guidelines. *International Journal of Social Psychiatry*. 2018;64(6):536-544. doi:10.1177/0020764018784624
4. Ross AM, Morgan AJ, Reavley NJ. Evaluation of the StigmaWatch Program's Impact on Media Portrayals of Suicide and Mental Illness. *Stigma and Health*. 2023;doi:10.1037/sah0000437

5. Tatum PT, Canetto SS, Slater MD. Suicide coverage in U.S. newspapers following the publication of the media guidelines. *Suicide and Life-Threatening Behavior*. 2010;40(5):524-534. doi:10.1521/suli.2010.40.5.524
6. Broncano-Rivera K, Contreras-Pizarro CH, Valle R. Suicide news on Peruvian television channels: evaluation of compliance with WHO's reporting recommendations. *Revista peruana de medicina experimental y salud publica*. 2023;40(3):307EP-316. doi:https://dx.doi.org/10.17843/rpmesp.2023.403.12776
7. Ribeiro E, Granado A. Assessing the quality of suicide reporting in Portugal: 6 case studies of celebrities and non-celebrities over 25 years. *International Review of Psychiatry*. 2024;36(4-5):469EP-479. doi:https://dx.doi.org/10.1080/09540261.2024.2327391
8. AlSheddi M. Between Guidelines and Headlines - Analysis of Newspapers Reporting of Suicide in Saudi Arabia. *Crisis*. Sep 2025;46(5):293-298. doi:10.1027/0227-5910/a001021
9. Araújo R, Lopes F. The portrayal of suicide in the Portuguese media between 2013 and 2017. *Cuadernosinfo*. 2022;(51):159-174. doi:10.7764/cdi.51.27613
10. Fong YL. Reporting on suicide in Malaysia: Problem characterization and solution advocacy by media. *KOME*. 2021;9(2):46-64. doi:10.17646/KOME.75672.66
11. Gould MS, Lake AM, Chowdhury S, et al. "Breaking the silence" suicide Prevention media campaign in Oregon: Evaluation of impact on help-seeking and suicide mortality. *Suicide and Life-Threatening Behavior*. 2024;54(2):361-369. doi:https://doi.org/10.1111/sltb.13047
12. Levi-Belz Y, Starostintzki Malonek R, Hamdan S. Trends in Newspaper Coverage of Suicide in Israel: An 8-Year Longitudinal Study. *Archives of Suicide Research*. 2023;27(4):1191-1206. doi:10.1080/13811118.2022.2111534
13. Mestas M, Arendt F. A Longitudinal Dynamic Perspective on Quality in Journalism: Investigating the Long-Term Macro-Level Media Effect of Suicide Reporting on Suicide Rates Across a Century. *Communication Research*. 2023;doi:10.1177/00936502221150315
14. Nabeshima Y, Onozuka D, Kitazono T, Hagihara A. Analysis of Japanese articles about suicides involving charcoal burning or hydrogen sulfide gas. *International Journal of Environmental Research and Public Health*. 2016;13(10)doi:10.3390/ijerph13101013
15. Jang J, Myung W, Kim S, et al. Effect of suicide prevention law and media guidelines on copycat suicide of general population following celebrity suicides in South Korea, 2005–2017. *Australian and New Zealand Journal of Psychiatry*. 2022;56(5):542-550. doi:10.1177/00048674211025701
16. Michel K, Frey C, Wyss K, Valach L. An exercise in improving suicide reporting in print media. *Crisis*. 2000;21(2):71-79. doi:10.1027//0227-5910.21.2.71
17. Schäfer R, Althaus D, Brosius HB, Hegerl U. Media coverage on suicide in Nuremberg's daily papers - Frequency and form of the reporting before and during media-intervention with guidelines. *Psychiatrische Praxis*. 2006;33(3):132-137. doi:10.1055/s-2005-915474
18. Abbott M, Ramchand R, Chamberlin M, Marcellino W. Detecting Changes in Newspaper Reporting of Suicide after a Statewide Social Marketing Campaign. *Health Communication*. 2018;33(6):674-680. doi:10.1080/10410236.2017.1298198
19. Arendt F, Markiewitz A, Scherr S. News for life: improving the quality of journalistic news reporting to prevent suicides. *Journal of Communication*. 2023;73(1):73-85. doi:10.1093/joc/jqac039
20. Etzersdorfer E, Sonneck G. Preventing suicide by influencing mass-media reporting. The viennese experience 1980–1996. *Archives of Suicide Research*. 1998;4(1):67-74. doi:10.1080/13811119808258290
21. Etzersdorfer E, Sonneck G, Nagel-Kuess S. Newspaper reports and suicide. *The New England Journal of Medicine*. 1992;327(7):502-503. doi:https://dx.doi.org/10.1056/NEJM199208133270720
22. Chu X, Zhang X, Cheng P, Schwebel DC, Hu G. Assessing the use of media reporting recommendations by the world health organization in suicide news published in the most influential media sources in china, 2003–2015. *International Journal of Environmental Research and Public Health*. 2018;15(3)doi:10.3390/ijerph15030451
23. Rodriguez-Caro CJR, Artiles FJA, Méndez MRC, Ramallo-Fariña Y, Garcimartin H. Differences in the adequacy of news about suicide in Spain vs. Victoria, Australia: are interventions aimed to the media effective? *REVISTA ESPANOLA DE SALUD PUBLICA*. 2021;95:E1-E14.
24. Cheng Q, Chen F, Lee EST, Yip PSF. The role of media in preventing student suicides: A Hong Kong experience. *Journal of Affective Disorders*. 2018;227:643-648. doi:10.1016/j.jad.2017.11.007
25. Cheng Q, Yip PSF. Implementing International Media Guidelines in a Local Context: Experiences from Hong Kong. 2017:241-250.
26. Lim JTY, Cheng Q, Ng YP, et al. Effectiveness of a Malaysian Media Intervention Workshop: Safe Reporting on Suicide. *Frontiers in Psychology*. 2021;12doi:10.3389/fpsyg.2021.666027

Studies included in the meta-analysis

This section lists all 15 studies used in our analysis. The complete list of included studies is as follows:

Acosta et al. 2020
Acosta FJ, Rodríguez CJ, Cejas MR, Ramallo-Fariña Y, Fernandez-Garcimartin H. Suicide coverage in the digital press media: adherence to World Health Organization guidelines and effectiveness of different interventions aimed at media professionals. *Health Communication* 2020; 35(13): 1623-32.

Fu & Yip 2008
Fu KW, Yip PSF. Changes in reporting of suicide news after the promotion of the WHO media recommendations. *Suicide and Life-Threatening Behavior* 2008; 38(5): 631-6.

Garrido-Fabián et al. 2018
Garrido-Fabián F, Serrano-López AE, Catalán-Matamoros D. The use of WHO recommendations for the prevention of suicide. The case of the newspaper Abc. *Revista Latina de Comunicacion Social* 2018; 73: 810-27.

Jamieson et al. 2003
Jamieson P, Jamieson KH, Romer D. The responsible reporting of suicide in print journalism. *American Behavioral Scientist* 2003; 46(12): 1643-60.

Ju et al. 2024
Ju Y, Scherr S, Arendt F, You M, Prieler M. Little Strokes Fell Big Oaks: how repeated recommendations for suicide reporting drive the quality of suicide news in South Korea. *Omega* 2024.

Kim 2005
Kim YJ. Reporting on suicide before and after the suicide guidelines presented. *Journal of Journalism & Communication Studies* 2005; 49(6): 140-65.

Niederkrotenthaler & Sonneck 2007
Niederkrotenthaler T, Sonneck G. Assessing the impact of media guidelines for reporting on suicides in Austria: interrupted time series analysis. *Australian and New Zealand Journal of Psychiatry* 2007; 41(5): 419-28.

Pirkis et al. 2009
Pirkis J, Dare A, Blood RW, et al. Changes in media reporting of suicide in Australia between 2000/01 and 2006/07. *Crisis* 2009; 30(1): 25-33.

Ramadas & Kuttichira 2011
Ramadas S, Kuttichira P. The development of a guideline and its impact on the media reporting of suicide. *Indian Journal of Psychiatry* 2011; 53(3): 224-8.

Roškar et al. 2017
Roškar S, Tančič Grum A, Poštuvan V, Podlesek A, De Leo D. The adaptation and implementation of guidelines for responsible media reporting on suicide in Slovenia. *Zdravstveno Varstvo* 2017; 56(1): 31-8.

Sinyor et al. 2024
Sinyor M, Ekstein D, Prabaharan N, et al. Changes in media reporting quality and suicides following national media engagement on responsible reporting of suicide in Canada. *The Canadian Journal of Psychiatry* 2024; 69(5):358-368.

Sinyor et al. 2021
Sinyor M, Kiss A, Williams M, et al. Changes in suicide reporting quality and deaths in Ontario following publication of national media guidelines. *Crisis* 2021; 42(5): 378-85.

Armstrong et al. 2025
Armstrong G, Haregu T, Jayaseelan M, et al. Progress towards prevention of suicide in India by improving print media reporting of suicide news: a repeat content analysis study in Tamil Nadu. *BMJ Open* 2025; 15(5): e092652.

Eriyanto 2025
Eriyanto. Do reporting guidelines improve the quality of suicide coverage? A case study of Indonesia. *Mental Health and Prevention* 2025; 40: 200463.

Shaw et al. 2025
Shaw C, Thomas T, Krishnamoorthy S, et al. Impact of media interventions on the quality and quantity of suicide media reporting in Guyana 2013-2023. *Journal of Affective Disorders* 2025; 381: 692-699.

Primary analysis forest plot – suicide rates outcome

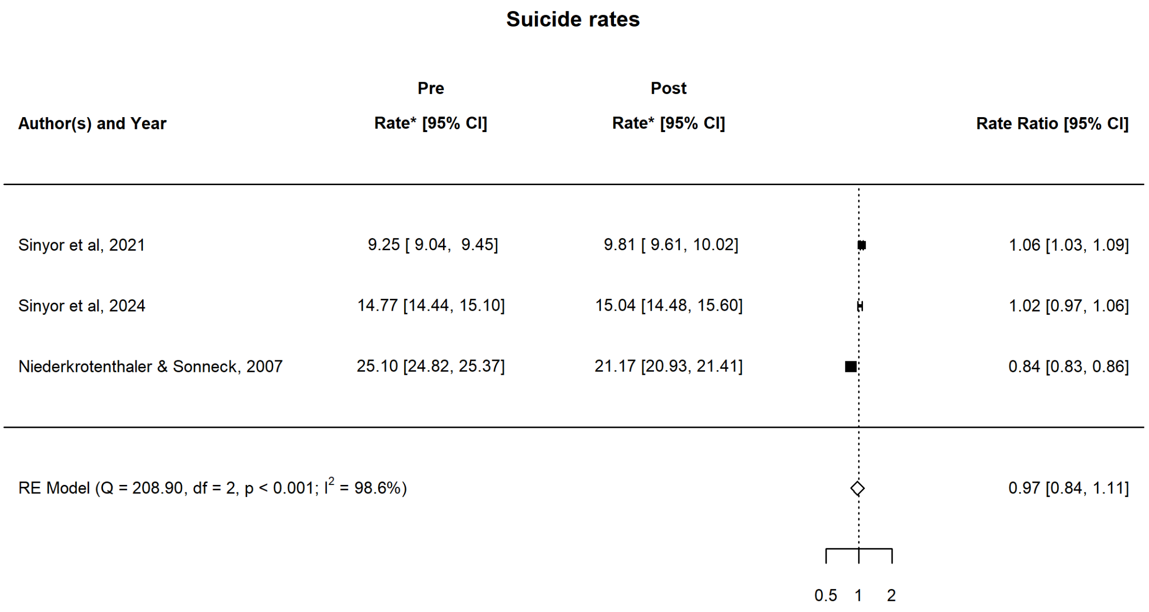

*Suicide rates per 100,000 person-years

Primary analysis forest plots – quality of suicide-related media reporting outcome

Do provide accurate information about where to seek help for suicidal thoughts and suicidal crises

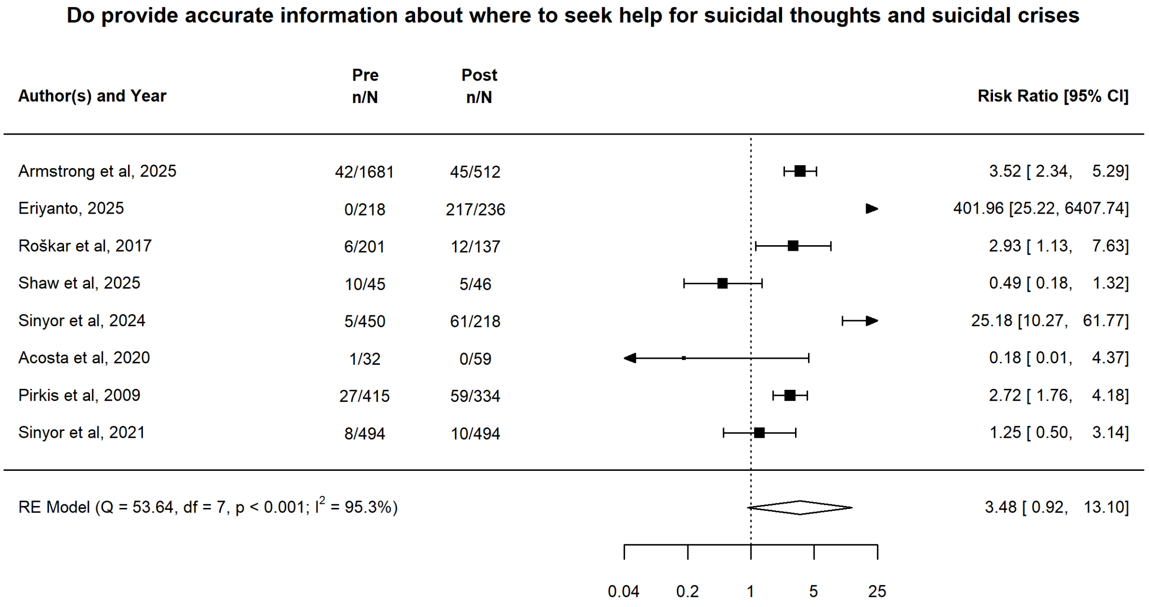

Do educate the public about the facts of suicide and suicide prevention based on accurate information

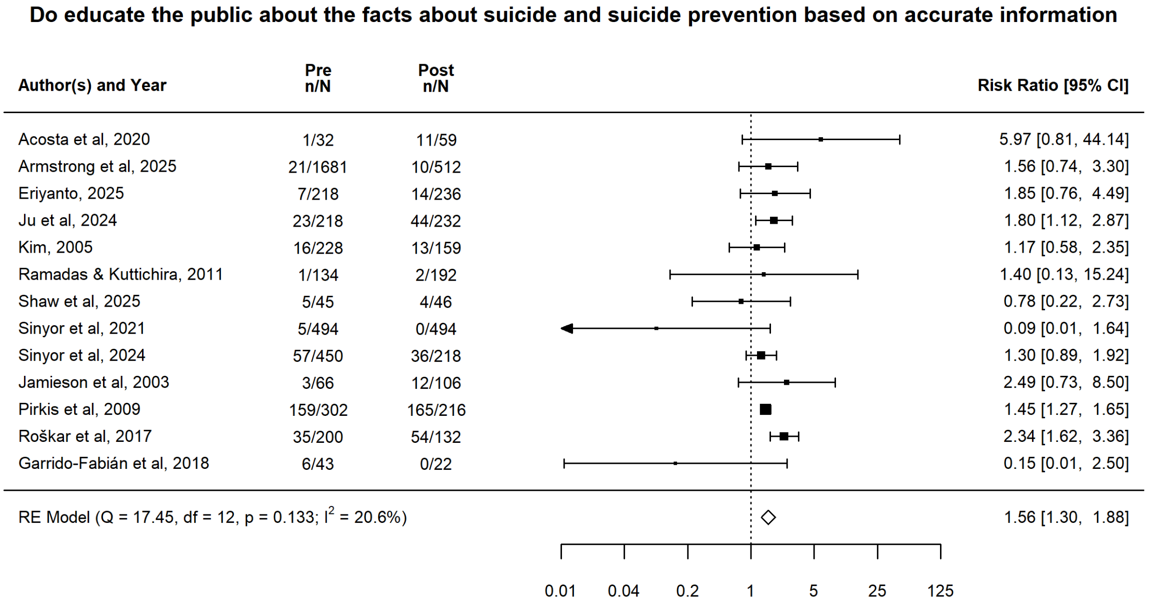

Do report stories of how to cope with life stressors and/or suicidal thoughts and the importance of help-seeking

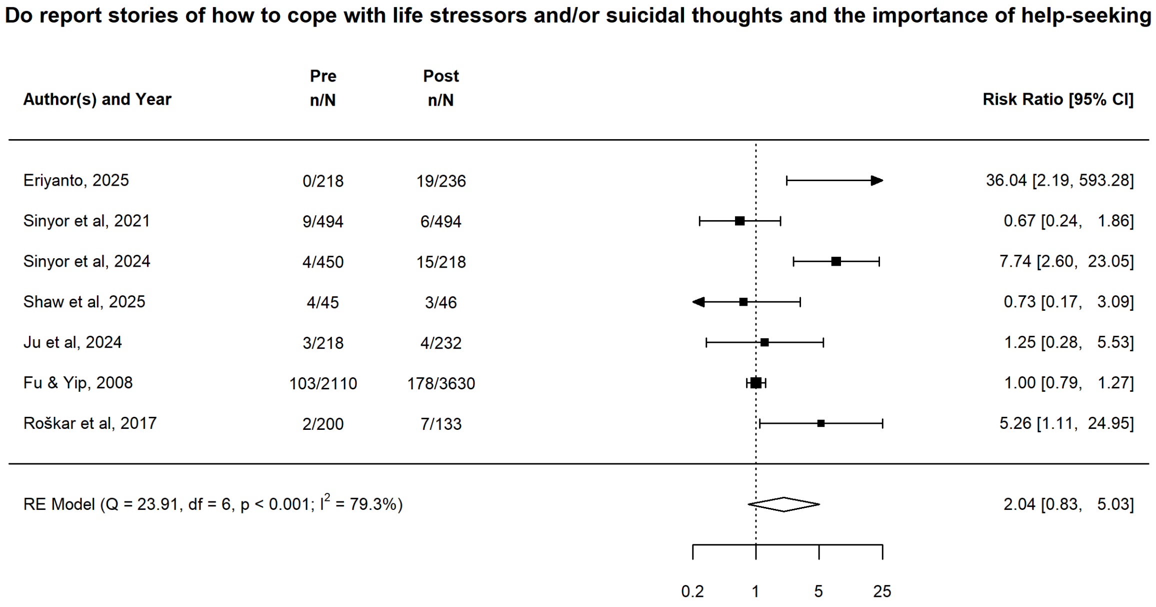

Do apply particular caution when reporting celebrity suicides

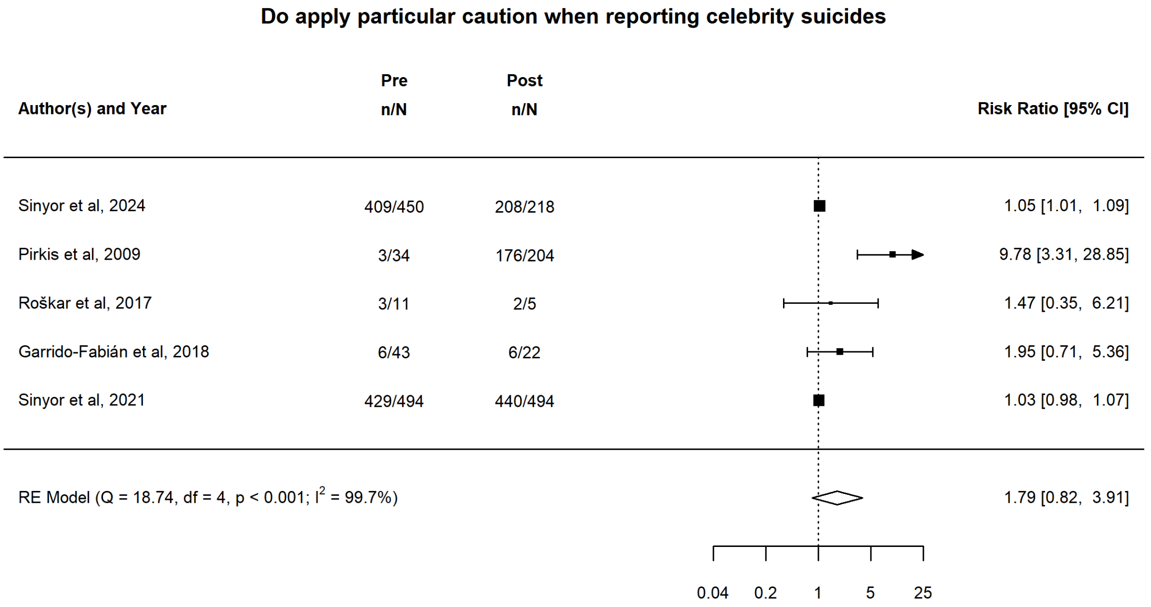

Do apply caution when interviewing bereaved family or friends or persons with lived experience

Do apply caution when interviewing bereaved family or friends or persons with lived experience

Author(s) and Year	Pre n/N	Post n/N	Risk Ratio [95% CI]
Eriyanto, 2025	201/218	224/236	1.03 [0.98, 1.08]
Armstrong et al, 2025	1635/1681	504/512	1.01 [1.00, 1.03]
Sinyor et al, 2024	362/450	193/218	1.10 [1.03, 1.18]
Pirkis et al, 2009	150/183	181/213	1.04 [0.95, 1.13]
Sinyor et al, 2021	392/494	389/494	0.99 [0.93, 1.06]
RE Model (Q = 6.94, df = 4, p = 0.139; I^2 = 41.9%)			1.03 [1.00, 1.06]

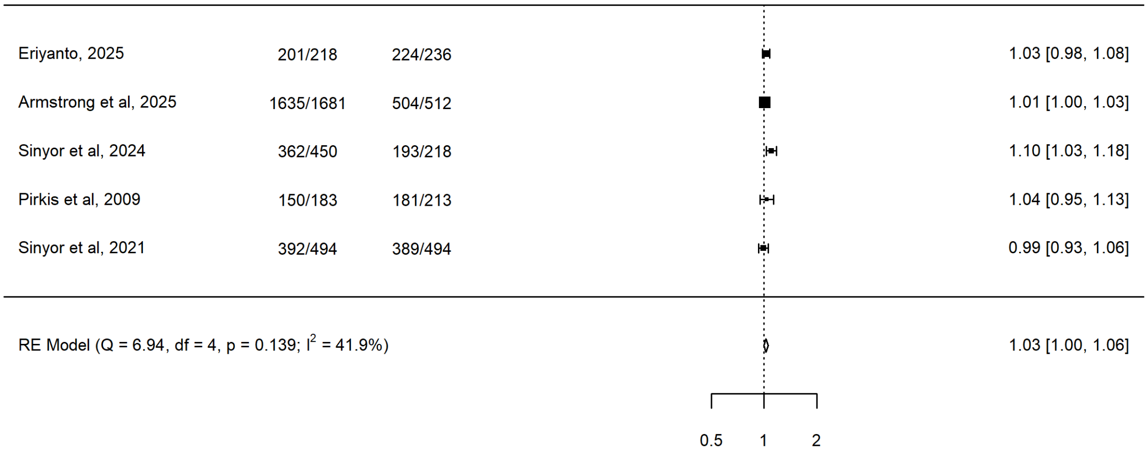

Don’t position suicide-related content as the top story and don’t unduly repeat such stories

Don’t position suicide-related content as the top story and don’t unduly repeat such stories

Author(s) and Year	Pre n/N	Post n/N	Risk Ratio [95% CI]
Acosta et al, 2020	22/32	33/59	0.81 [0.59, 1.13]
Armstrong et al, 2025	1598/1681	503/512	1.03 [1.02, 1.05]
Jamieson et al, 2003	41/66	52/106	0.79 [0.60, 1.03]
Fu & Yip, 2008	2070/2110	3565/3630	1.00 [0.99, 1.01]
Pirkis et al, 2009	345/415	158/205	0.93 [0.85, 1.01]
Ramadas & Kuttichira, 2011	82/134	110/192	0.94 [0.78, 1.12]
RE Model (Q = 21.46, df = 5, p < 0.001; I^2 = 92.8%)			0.98 [0.92, 1.03]

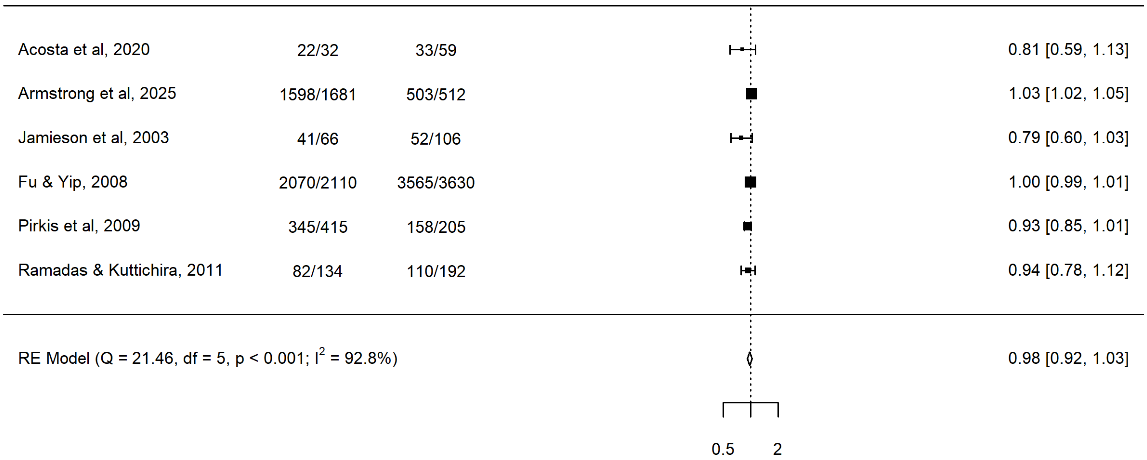

Don’t describe the method used

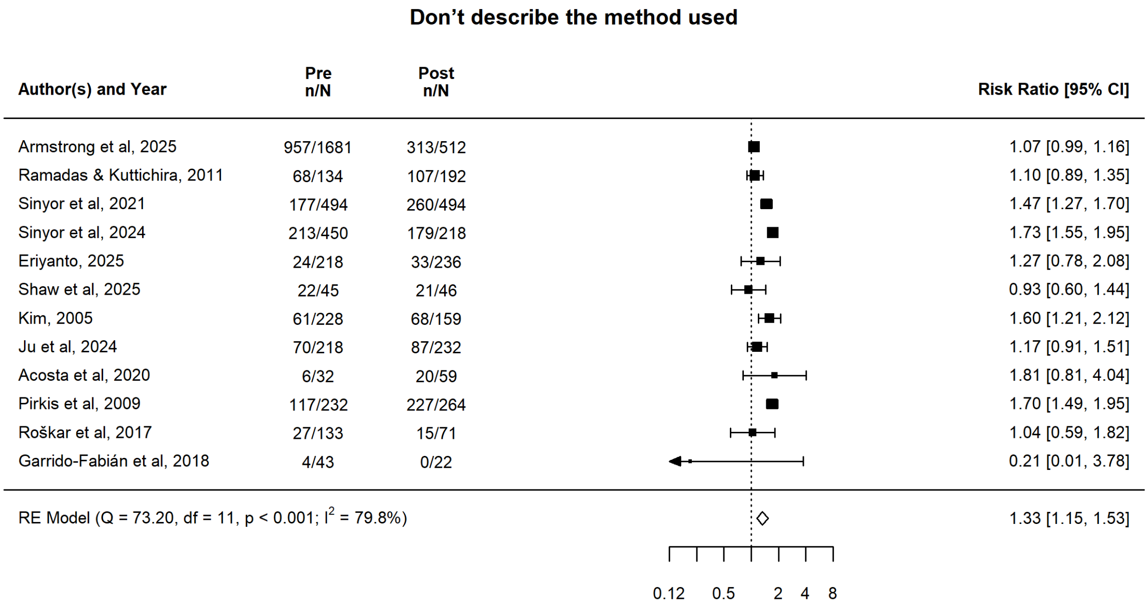

Don’t name or provide details about the site/location

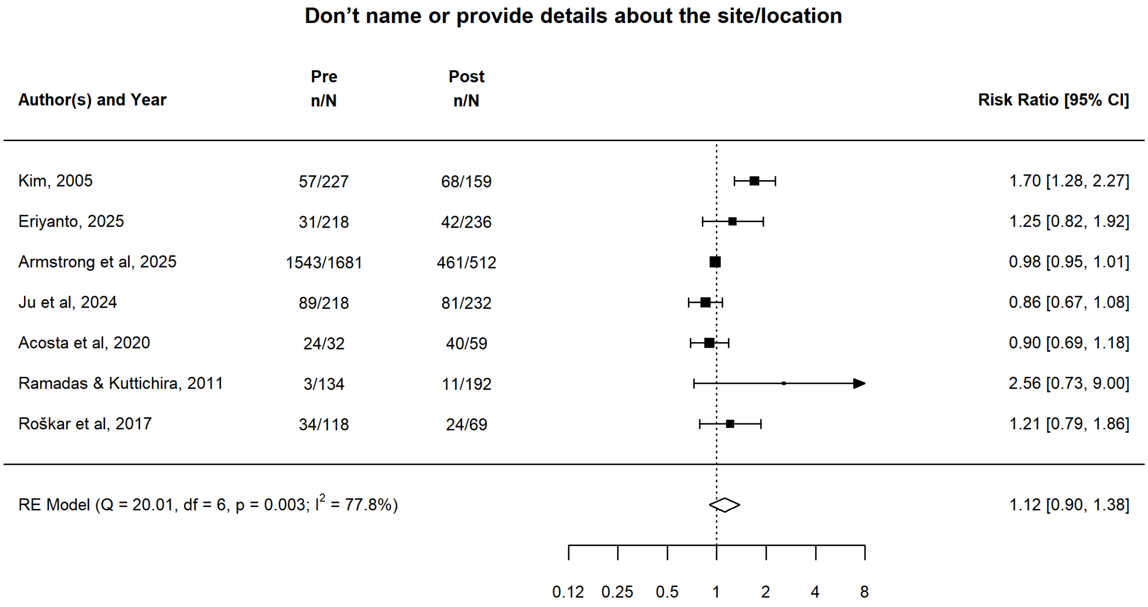

Don’t use language/content which sensationalizes, romanticizes or normalizes suicide, or that presents it as a viable solution to problems

Don’t use language/content which sensationalizes, romanticizes or normalizes suicide, or that presents it as a viable solution to problems

Author(s) and Year	Pre n/N	Post n/N	Risk Ratio [95% CI]
Acosta et al, 2020	32/32	59/59	1.01 [0.96, 1.06]
Garrido-Fabián et al, 2018	26/43	22/22	1.62 [1.27, 2.08]
Ju et al, 2024	30/218	29/232	0.91 [0.56, 1.46]
Kim, 2005	186/228	121/159	0.93 [0.84, 1.04]
Ramadas & Kuttichira, 2011	71/134	96/192	0.94 [0.76, 1.17]
Shaw et al, 2025	36/45	35/46	0.95 [0.76, 1.18]
Sinyor et al, 2021	492/494	493/494	1.00 [1.00, 1.01]
Sinyor et al, 2024	406/450	212/218	1.08 [1.04, 1.12]
Eriyanto, 2025	214/218	227/236	0.98 [0.95, 1.01]
Pirkis et al, 2009	242/415	326/347	1.61 [1.48, 1.76]
Roškar et al, 2017	65/200	77/140	1.69 [1.32, 2.17]
RE Model (Q = 166.79, df = 10, p < 0.001; I^2 = 98.8%)			1.12 [0.98, 1.29]

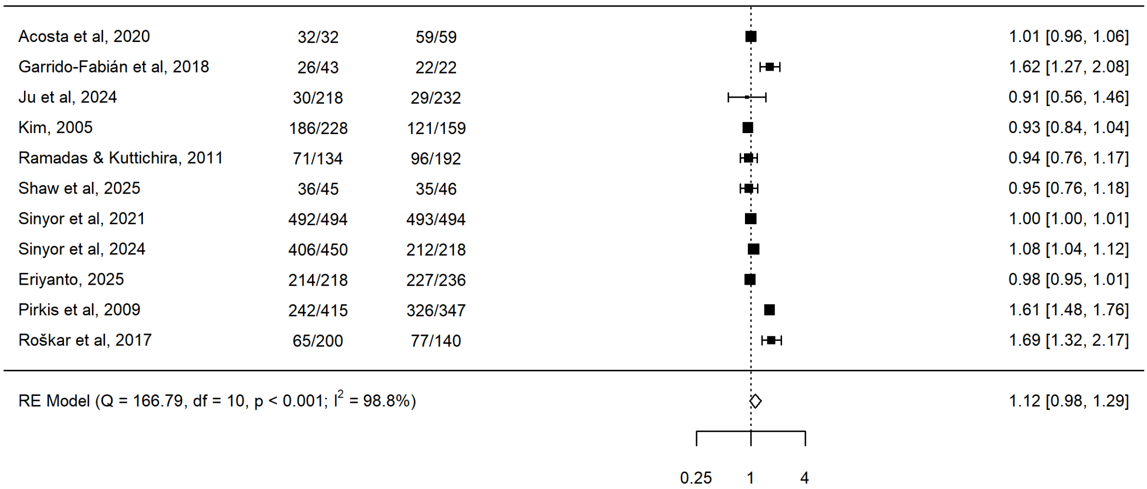

Don’t oversimplify the reason for a suicide or reduce it to a single factor

Don’t oversimplify the reason for a suicide or reduce it to a single factor

Author(s) and Year	Pre n/N	Post n/N	Risk Ratio [95% CI]
Armstrong et al, 2025	315/1681	108/512	1.13 [0.93, 1.37]
Eriyanto, 2025	70/218	83/236	1.10 [0.85, 1.42]
Kim, 2005	105/228	83/159	1.13 [0.92, 1.39]
Ju et al, 2024	70/218	83/232	1.11 [0.86, 1.44]
Sinyor et al, 2024	411/450	173/218	0.87 [0.81, 0.94]
Acosta et al, 2020	18/32	57/59	1.72 [1.26, 2.34]
Jamieson et al, 2003	20/66	49/106	1.53 [1.00, 2.32]
Ramadas & Kuttichira, 2011	77/134	116/192	1.05 [0.87, 1.27]
Garrido-Fabián et al, 2018	17/43	17/22	1.95 [1.27, 3.02]
Sinyor et al, 2021	418/494	460/494	1.10 [1.05, 1.15]
RE Model (Q = 51.70, df = 9, p < 0.001; I^2 = 86.1%)			1.16 [1.02, 1.33]

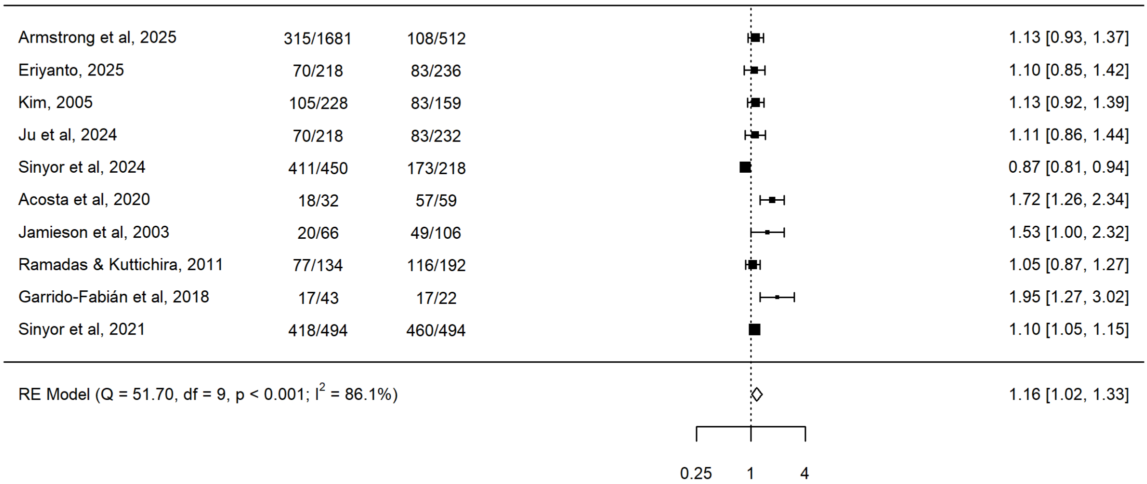

Don’t use sensational language in headlines

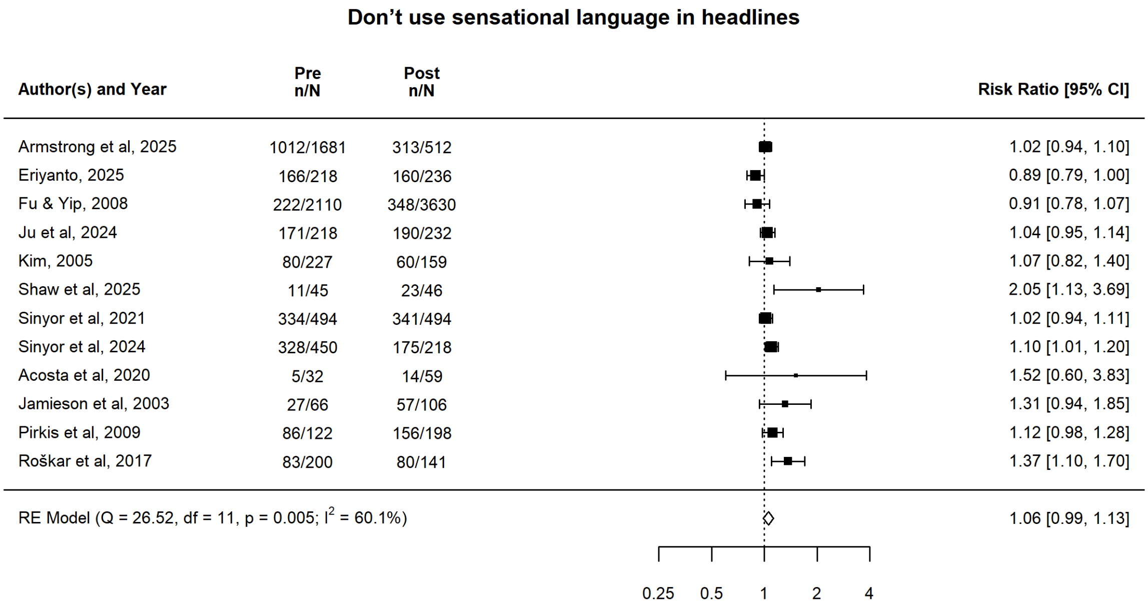

Don’t use photographs, video footage, audio recordings, digital or social media links

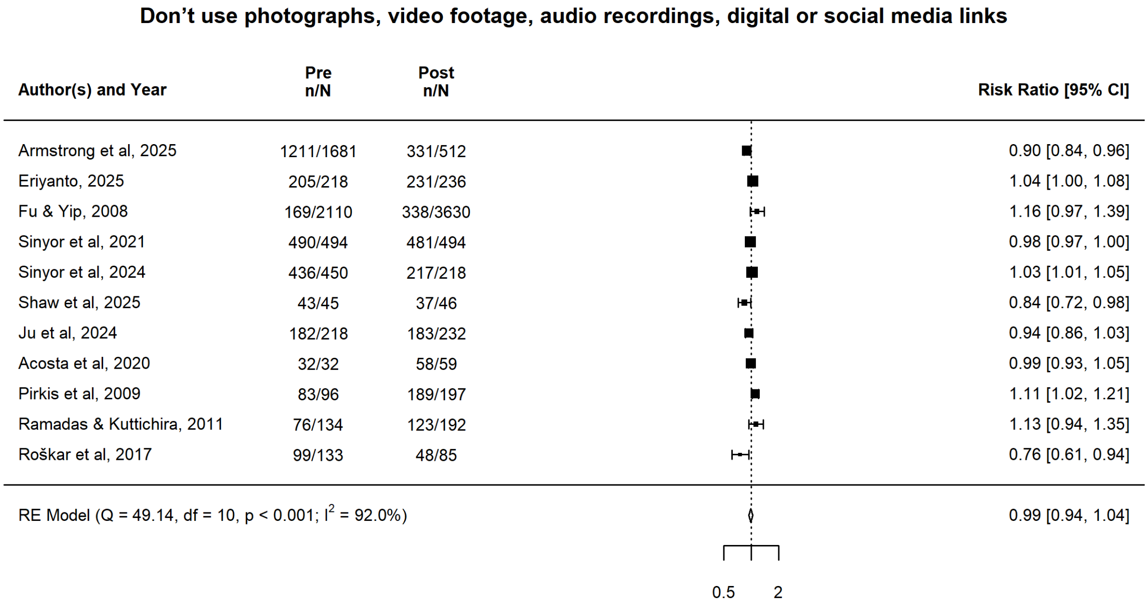

Don’t report the details of a suicide note

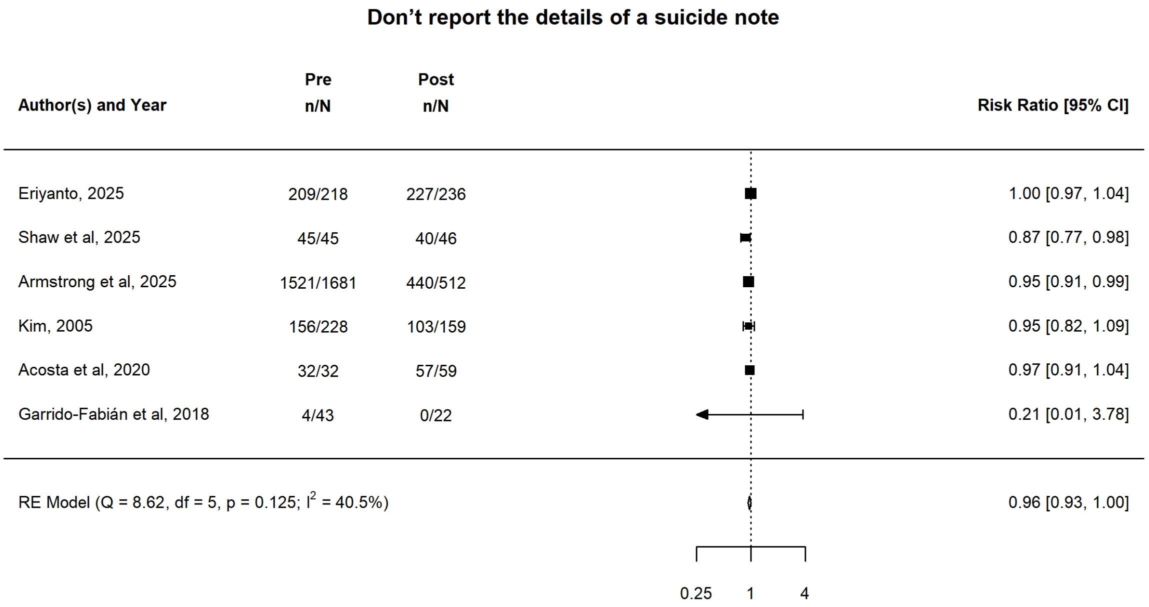

eTable 2. Summary of pooled results for sensitivity analysis

Studies **with a gap** between implementation of guidelines and start of post-intervention assessment period

Outcome	k	Pooled RR/IRR (95%CI)	I^2	Favours Guidelines
**Quality of media reporting**				
• Do provide accurate information about where to seek help for suicidal thoughts and suicidal crises	4	RR=5.07 (95%CI=0.33-78.94)	98.1%	Yes
• Do educate the public about the facts of suicide and suicide prevention based on accurate information	5	RR=1.49 (95%CI=1.32-1.68)	0.0%	Yes
• Do report stories of how to cope with life stressors and/or suicidal thoughts and the importance of help-seeking	NA	NA	NA	NA
• Do apply particular caution when reporting celebrity suicides	NA	NA	NA	NA
• Do apply caution when interviewing bereaved family or friends or persons with lived experience	3	RR=1.01 (95%CI=1.00-1.03)	0.0%	Yes
• Don't position suicide-related content as the top story and don't unduly repeat such stories	3	RR=0.97 (95%CI=0.87-1.08)	74.5%	No
• Don't describe the method used	5	RR=1.32 (95%CI=1.05-1.65)	82.2%	Yes
• Don't name or provide details about the site/location	4	RR=0.98 (95%CI=0.95-1.01)	0.1%	No
• Don't use language/content which sensationalizes, romanticizes or normalizes suicide, or that presents it as a viable solution to problems	4	RR=1.12 (95%CI=0.86-1.46)	98.5%	Yes
• Don't oversimplify the reason for a suicide or reduce it to a single factor	4	RR=1.22 (95%CI=1.01-1.46)	53.3%	Yes
• Don't use sensational language in headlines	5	RR=1.01 (95%CI=0.93-1.10)	54.2%	Yes
• Don't use photographs, video footage, audio recordings, digital or social media links	5	RR=0.99 (95%CI=0.93-1.07)	83.1%	No
• Don't report the details of a suicide note	3	RR=0.98 (95%CI=0.94-1.01)	51.0%	No
**Suicides**	NA	NA	NA	NA

Note. k = number of studies contributing to each pooled estimate. RR = risk ratio; IRR = incidence rate ratio; CI = confidence interval. Effect sizes compare post-guideline with pre-guideline periods and are pooled across studies. For the quality of reporting outcomes, RR > 1 indicates greater adherence post-guideline. For the suicide outcome, IRR < 1 indicates fewer deaths post-guideline. I^2 quantifies between-study heterogeneity. "Favours guidelines" reflects the direction of the point estimate (RR > 1 for reporting quality; IRR < 1 for suicides). NA = not applicable as no studies assessed this recommendation/outcome in this analysis.

eTable 2 (cont): Summary of pooled results for sensitivity analysis

Studies **with no gap** between implementation of guidelines and start of post-intervention assessment period

Outcome	k	Pooled RR/IRR (95%CI)	I²	Favours Guidelines
**Quality of media reporting**				
• Do provide accurate information about where to seek help for suicidal thoughts and suicidal crises	4	RR=2.61 (95%CI=0.50-13.63)	91.9%	Yes
• Do educate the public about the facts of suicide and suicide prevention based on accurate information	8	RR=1.40 (95%CI=0.93-2.11)	42.6%	Yes
• Do report stories of how to cope with life stressors and/or suicidal thoughts and the importance of help-seeking	5	RR=1.72 (95%CI=0.64-4.63)	82.0%	Yes
• Do apply particular caution when reporting celebrity suicides	4	RR=1.04 (95%CI=1.01-1.07)	0.0%	Yes
• Do apply caution when interviewing bereaved family or friends or persons with lived experience	NA	NA	NA	NA
• Don't position suicide-related content as the top story and don't unduly repeat such stories	3	RR=0.96 (95%CI=0.87-1.06)	40.9%	No
• Don't describe the method used	7	RR=1.34 (95%CI=1.10-1.63)	75.3%	Yes
• Don't name or provide details about the site/location	3	RR=1.54 (95%CI=1.15-2.07)	20.4%	Yes
• Don't use language/content which sensationalizes, romanticizes or normalizes suicide, or that presents it as a viable solution to problems	7	RR=1.12 (95%CI=0.94-1.34)	98.1%	Yes
• Don't oversimplify the reason for a suicide or reduce it to a single factor	6	RR=1.15 (95%CI=0.95-1.39)	92.4%	Yes
• Don't use sensational language in headlines	7	RR=1.12 (95%CI=0.99-1.26)	70.9%	Yes
• Don't use photographs, video footage, audio recordings, digital or social media links	6	RR=0.98 (95%CI=0.88-1.09)	97.4%	No
• Don't report the details of a suicide note	3	RR=0.90 (95%CI=0.82-0.99)	0.0%	No
**Suicides**	3	IRR=0.97 (95%CI=0.84-1.11)	98.6%	Yes

Note. k = number of studies contributing to each pooled estimate. RR = risk ratio; IRR = incidence rate ratio; CI = confidence interval. Effect sizes compare post-guideline with pre-guideline periods and are pooled across studies. For the quality of reporting outcomes, RR > 1 indicates greater adherence post-guideline. For the suicide outcome, IRR < 1 indicates fewer deaths post-guideline. I^2 quantifies between-study heterogeneity. "Favours guidelines" reflects the direction of the point estimate (RR > 1 for reporting quality; IRR < 1 for suicides). NA = not applicable as no studies assessed this recommendation/outcome in this analysis.

Sensitivity analysis forest plots – suicide rates outcome

Studies with no gap between guideline release and the start of the post-intervention assessment period

Note that the three studies that assessed suicide as an outcome (Niederkrotenthaler & Sonneck, 2007; Sinyor et al., 2021; Sinyor et al., 2024) had no gap between the implementation of guidelines and the start of the post-intervention assessment period. Therefore, the forest plot here is the same as its primary analysis forest plot.

Suicide rates

Author(s) and Year	Pre Rate* [95% CI]	Post Rate* [95% CI]	Rate Ratio [95% CI]
Sinyor et al, 2021	9.25 [9.04, 9.45]	9.81 [9.61, 10.02]	1.06 [1.03, 1.09]
Sinyor et al, 2024	14.77 [14.44, 15.10]	15.04 [14.48, 15.60]	1.02 [0.97, 1.06]
Niederkrotenthaler & Sonneck, 2007	25.10 [24.82, 25.37]	21.17 [20.93, 21.41]	0.84 [0.83, 0.86]
RE Model (Q = 208.90, df = 2, p < 0.001; I^2 = 98.6%)			0.97 [0.84, 1.11]

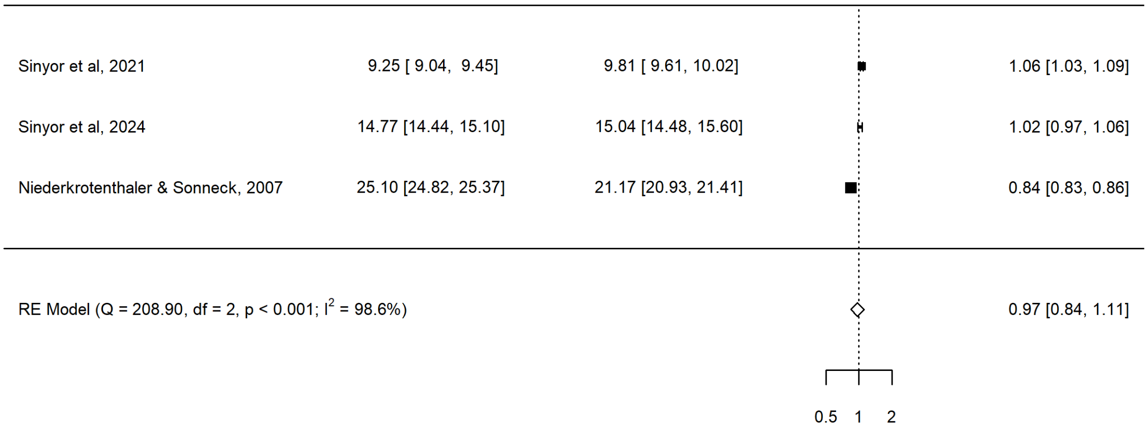

*Suicide rates per 100,000 person-years

Sensitivity analysis forest plots – quality of suicide-related media reporting outcome

Studies <u>with a gap</u> between guideline release and the start of the post-intervention assessment period

Note that the five studies (Armstrong et al., 2025; Eriyanto, 2025; Acosta et al., 2020; Ju et al., 2024; Pirkis et al., 2009) <u>with a gap</u> between the implementation of guidelines and the start of the post-intervention assessment period measured some but not all WHO/IASP (2023) recommendations.

Do provide accurate information about where to seek help for suicidal thoughts and suicidal crises

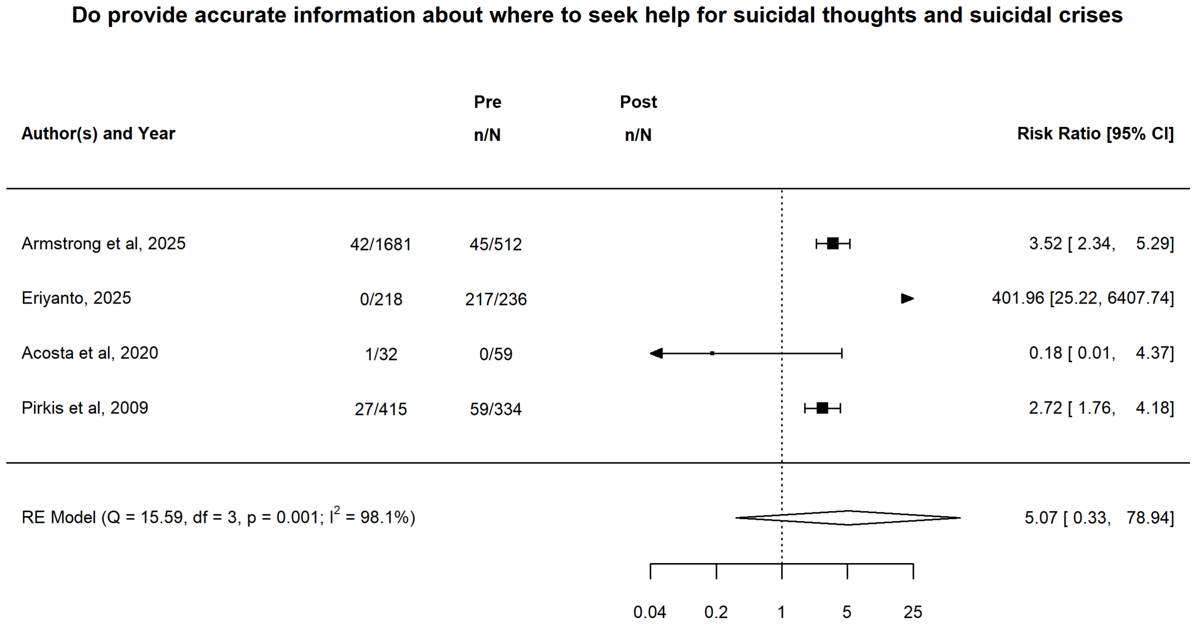

Do educate the public about the facts of suicide and suicide prevention based on accurate information

Do report stories of how to cope with life stressors and/or suicidal thoughts and the importance of help-seeking

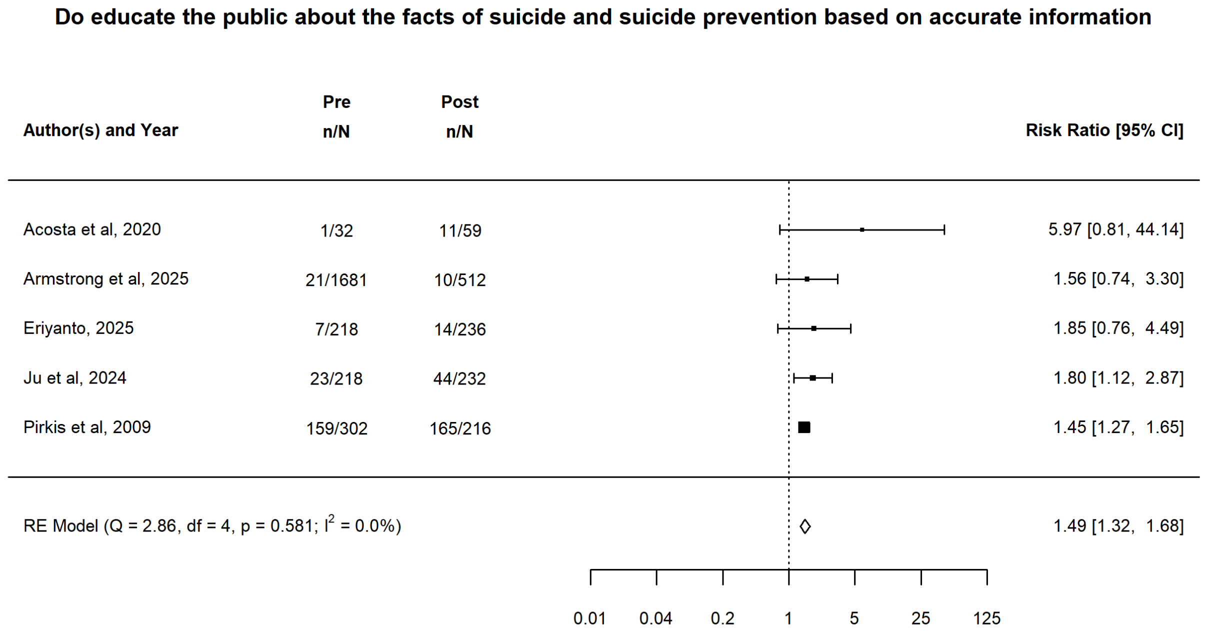

No studies with a gap between guideline release and the start of the post-intervention assessment period assessed this recommendation.

Do apply particular caution when reporting celebrity suicides

No studies with a gap between guideline release and the start of the post-intervention assessment period assessed this recommendation.

Do apply caution when interviewing bereaved family or friends or persons with lived experience

Don't position suicide-related content as the top story and don't unduly repeat such stories

Do apply caution when interviewing bereaved family or friends or persons with lived experience

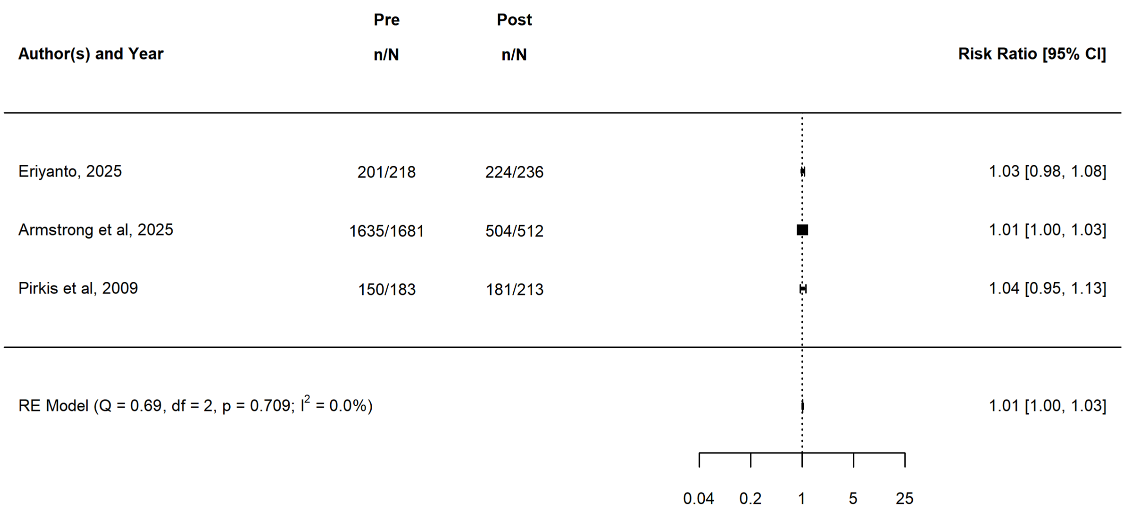

Don't position suicide-related content as the top story and don't unduly repeat such stories

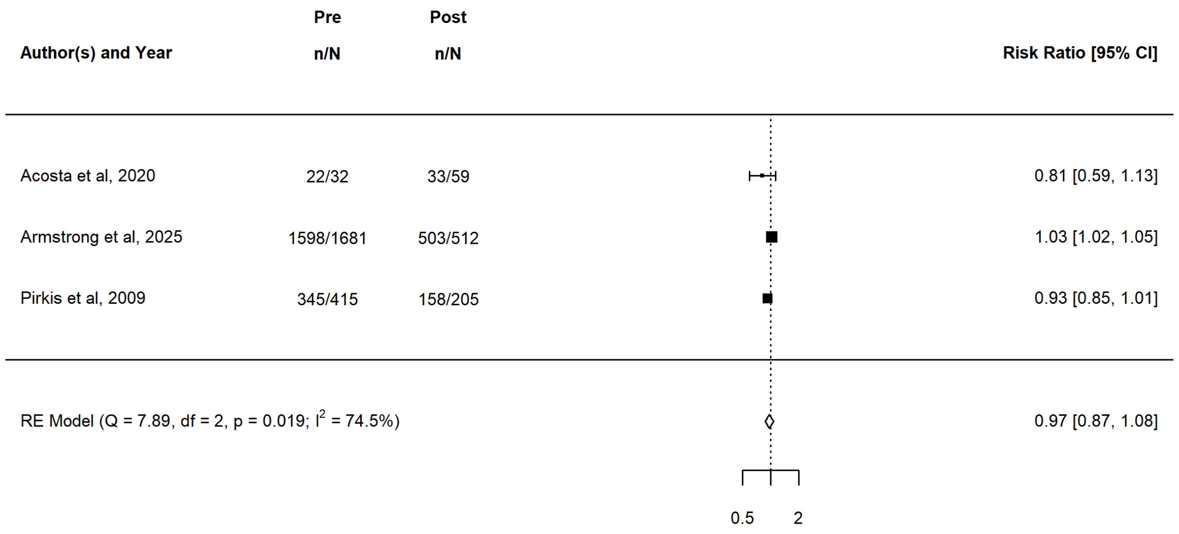

Don't describe the method used

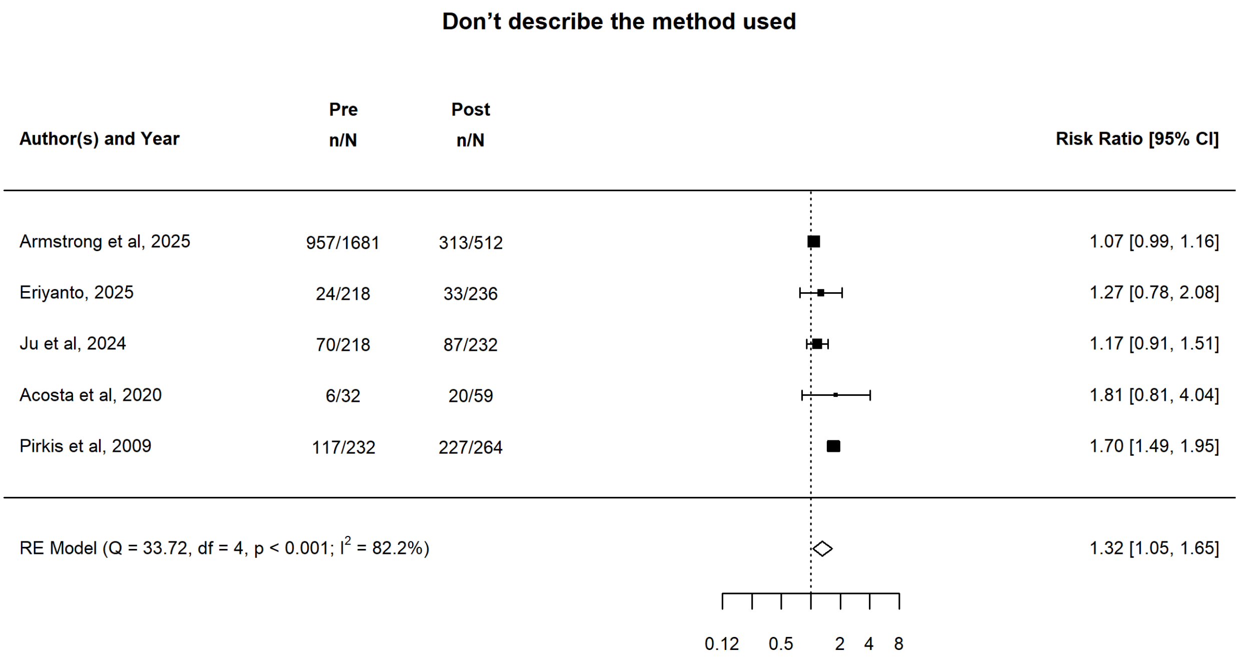

Don't name or provide details about the site/location

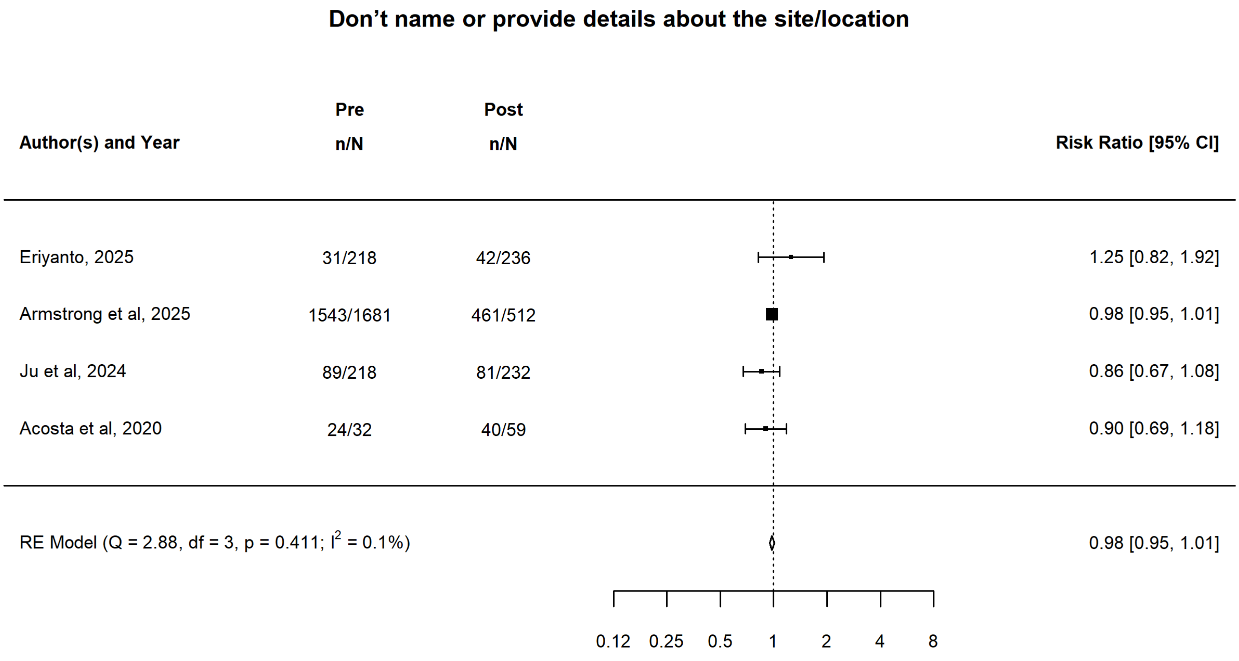

Don’t use language/content which sensationalizes, romanticizes or normalizes suicide, or that presents it as a viable solution to problems

Don’t use language/content which sensationalizes, romanticizes or normalizes suicide, or that presents it as a viable solution to problems

Author(s) and Year	Pre n/N	Post n/N	Risk Ratio [95% CI]
Acosta et al, 2020	32/32	59/59	1.01 [0.96, 1.06]
Ju et al, 2024	30/218	29/232	0.91 [0.56, 1.46]
Eriyanto, 2025	214/218	227/236	0.98 [0.95, 1.01]
Pirkis et al, 2009	242/415	326/347	1.61 [1.48, 1.76]
RE Model (Q = 115.81, df = 3, $p < 0.001$; I^2 = 98.5%)			1.12 [0.86, 1.46]

Axis: 0.25 1 4

Don’t oversimplify the reason for a suicide or reduce it to a single factor

Don’t oversimplify the reason for a suicide or reduce it to a single factor

Author(s) and Year	Pre n/N	Post n/N	Risk Ratio [95% CI]
Armstrong et al, 2025	315/1681	108/512	1.13 [0.93, 1.37]
Eriyanto, 2025	70/218	83/236	1.10 [0.85, 1.42]
Ju et al, 2024	70/218	83/232	1.11 [0.86, 1.44]
Acosta et al, 2020	18/32	57/59	1.72 [1.26, 2.34]
RE Model (Q = 6.36, df = 3, p = 0.095; I^2 = 53.3%)			1.22 [1.01, 1.46]

Axis: 0.25 1 4

Don’t use sensational language in headlines

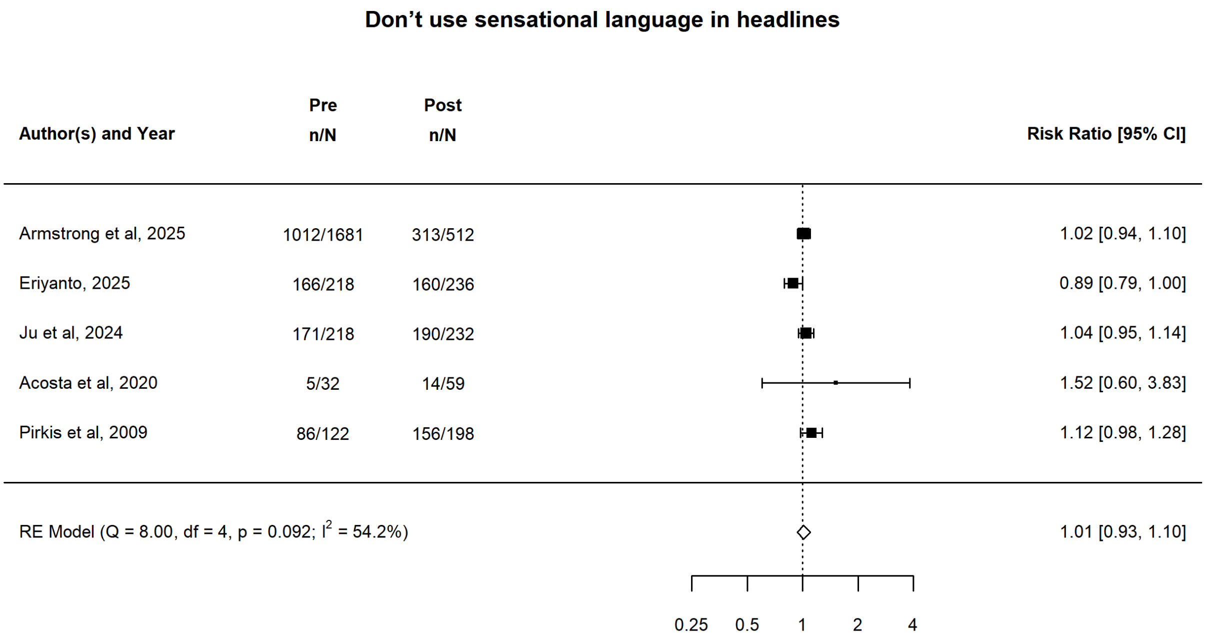

Don’t use photographs, video footage, audio recordings, digital or social media links

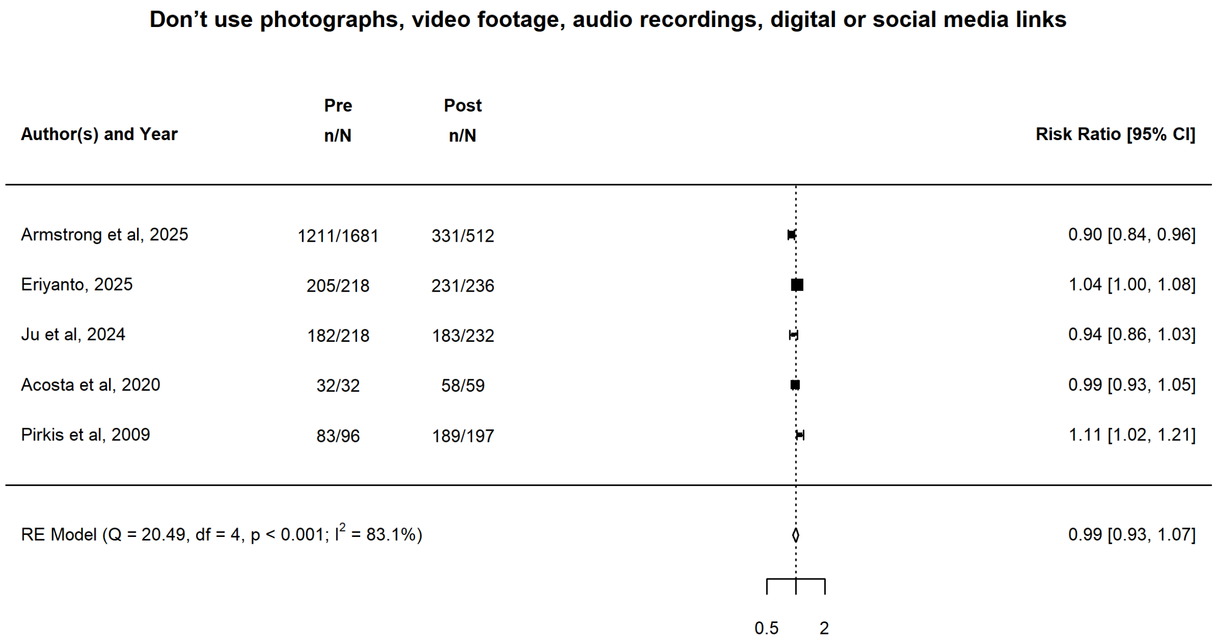

Don’t report the details of a suicide note

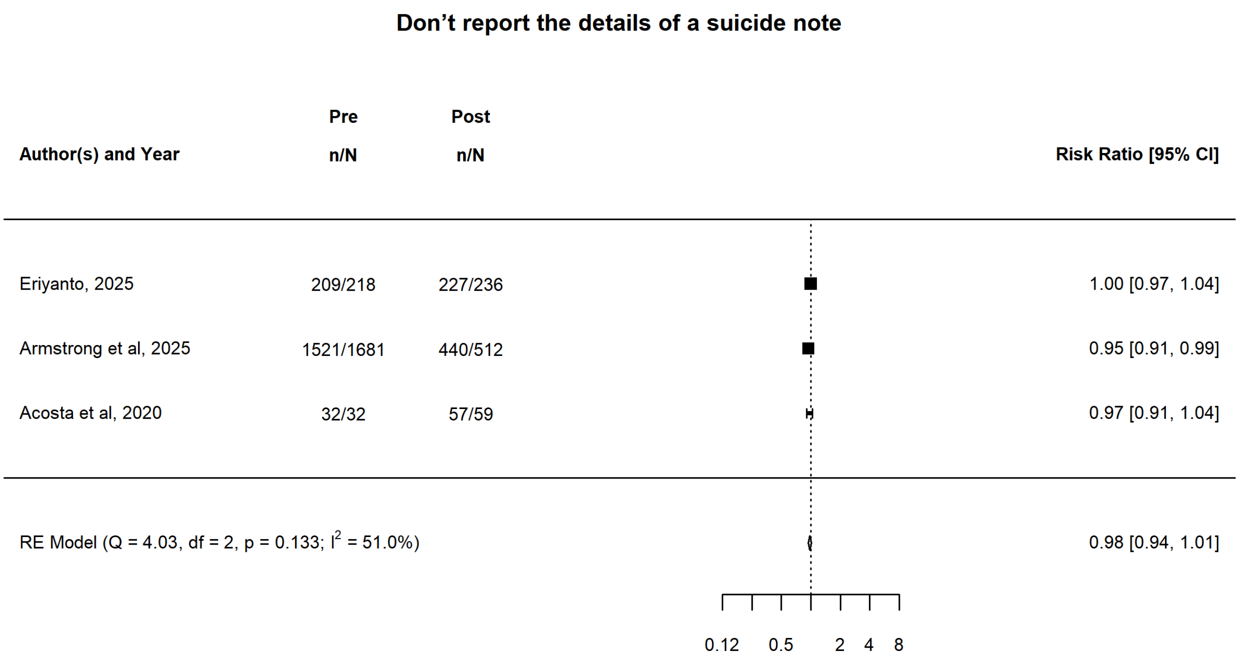

Studies with no gap between guideline release and the start of the post-intervention assessment period

Note that the remaining eight studies (Shaw et al. 2025; Fu & Yip, 2008; Garrido-Fabián et al., 2018; Jamieson et al., 2003; Kim 2005; Ramadas & Kuttichira, 2011; Roškar et al., 2017; Sinyor et al., 2021; Sinyor et al., 2024) with no gap between the implementation of guidelines and the start of the post-intervention assessment period measured some but not all WHO/IASP (2023) recommendations.

Do provide accurate information about where to seek help for suicidal thoughts and suicidal crises

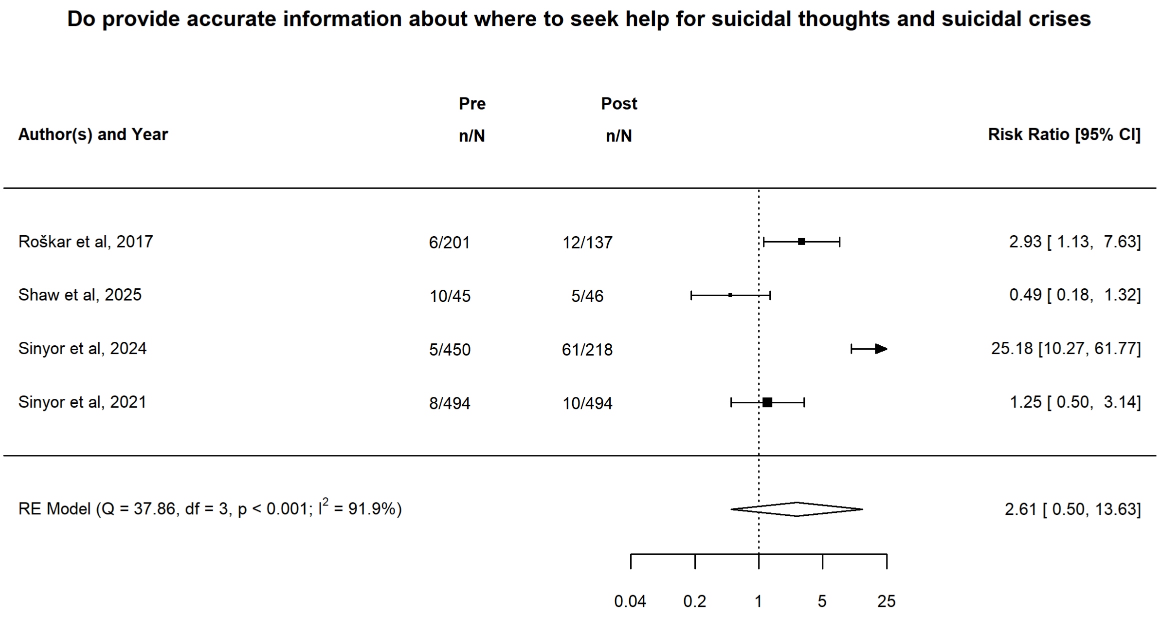

Do educate the public about the facts of suicide and suicide prevention based on accurate information

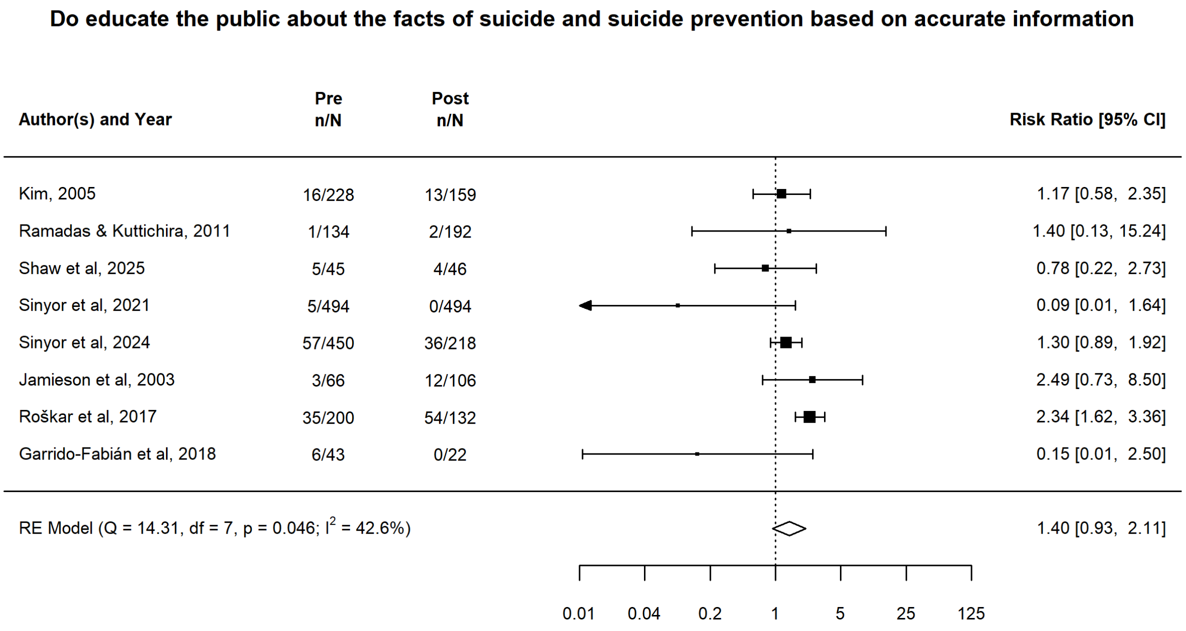

Do report stories of how to cope with life stressors and/or suicidal thoughts and the importance of help-seeking

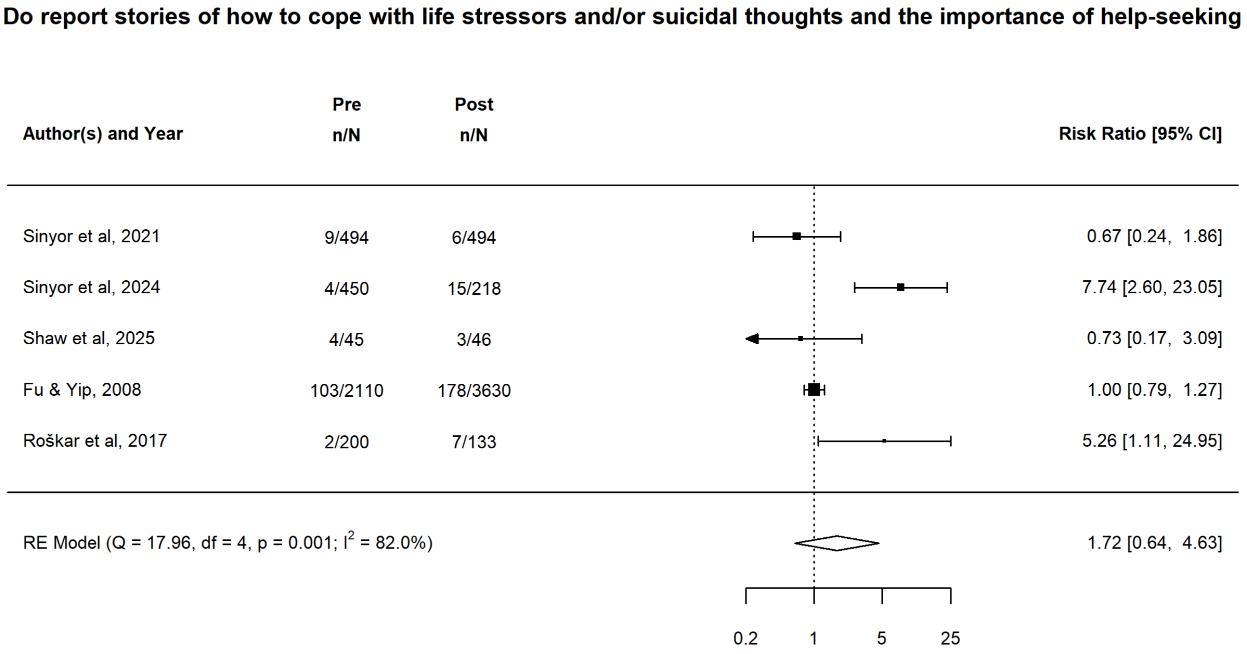

Do apply particular caution when reporting celebrity suicides

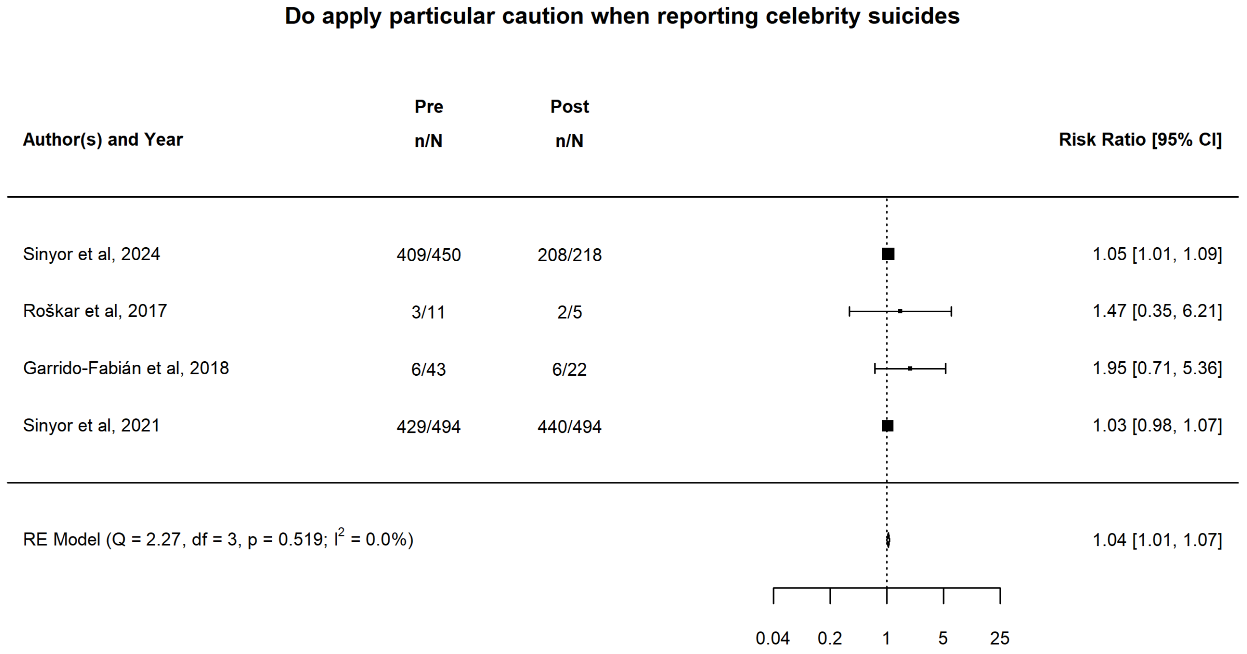

Do apply caution when interviewing bereaved family or friends or persons with lived experience

No studies with no gap between guideline release and the start of the post-intervention assessment period assessed this recommendation.

Don't position suicide-related content as the top story and don't unduly repeat such stories

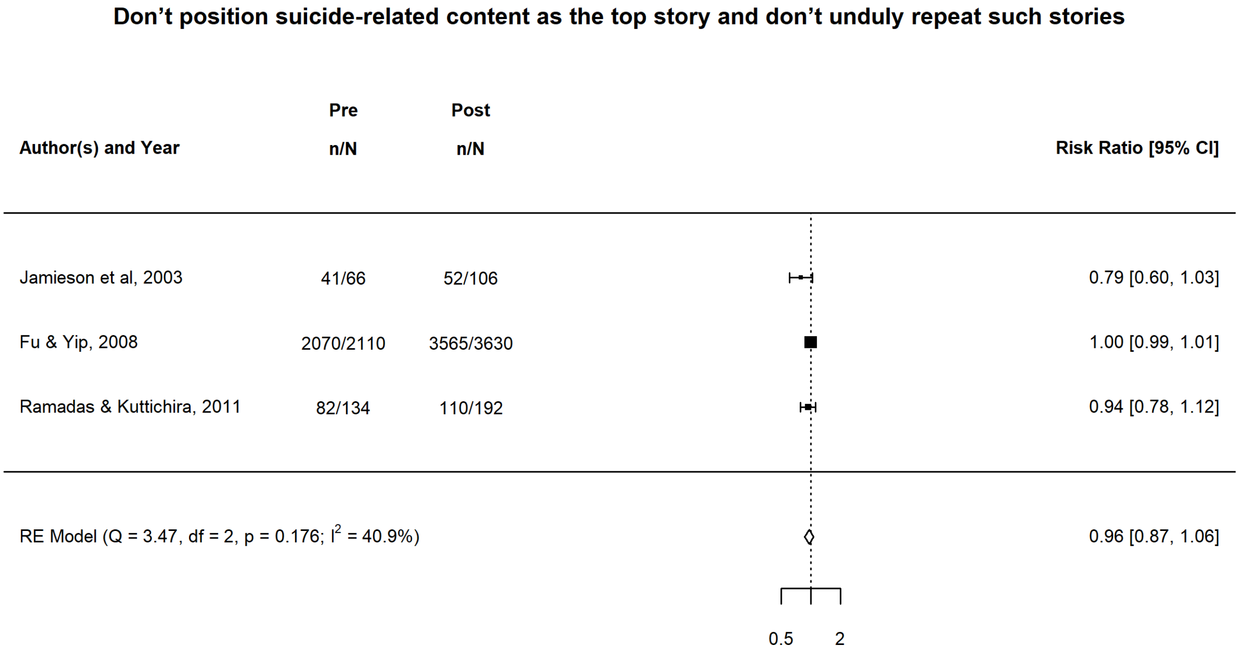

Don't describe the method used

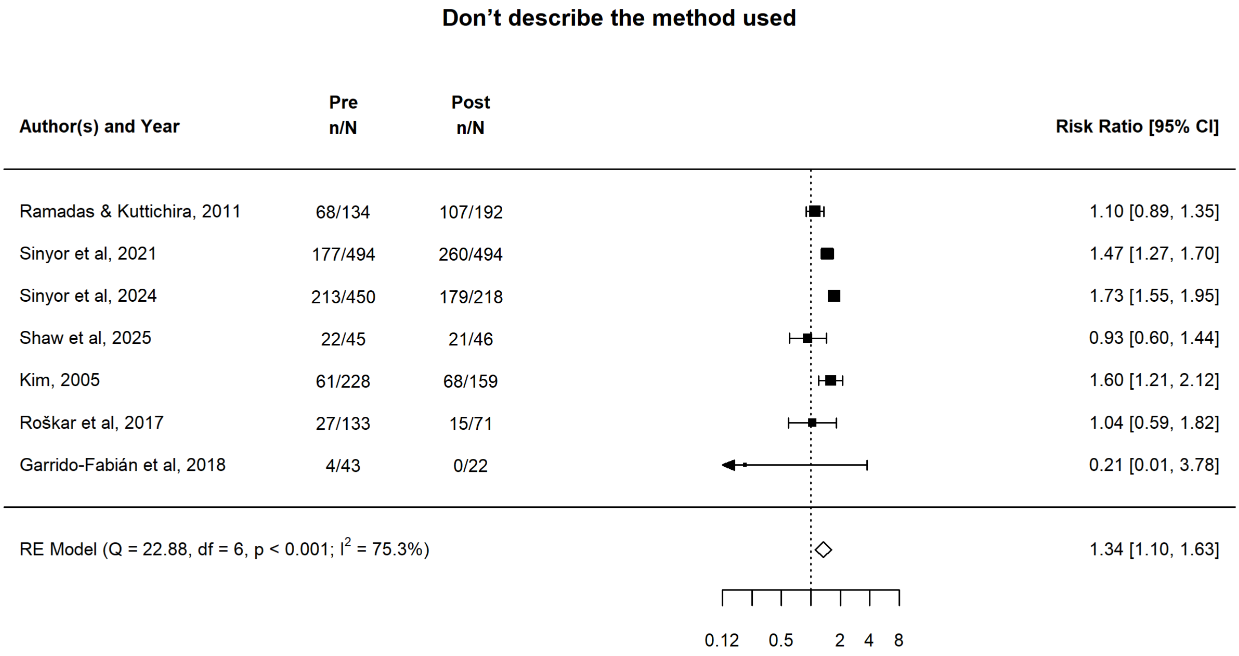

Don’t name or provide details about the site/location

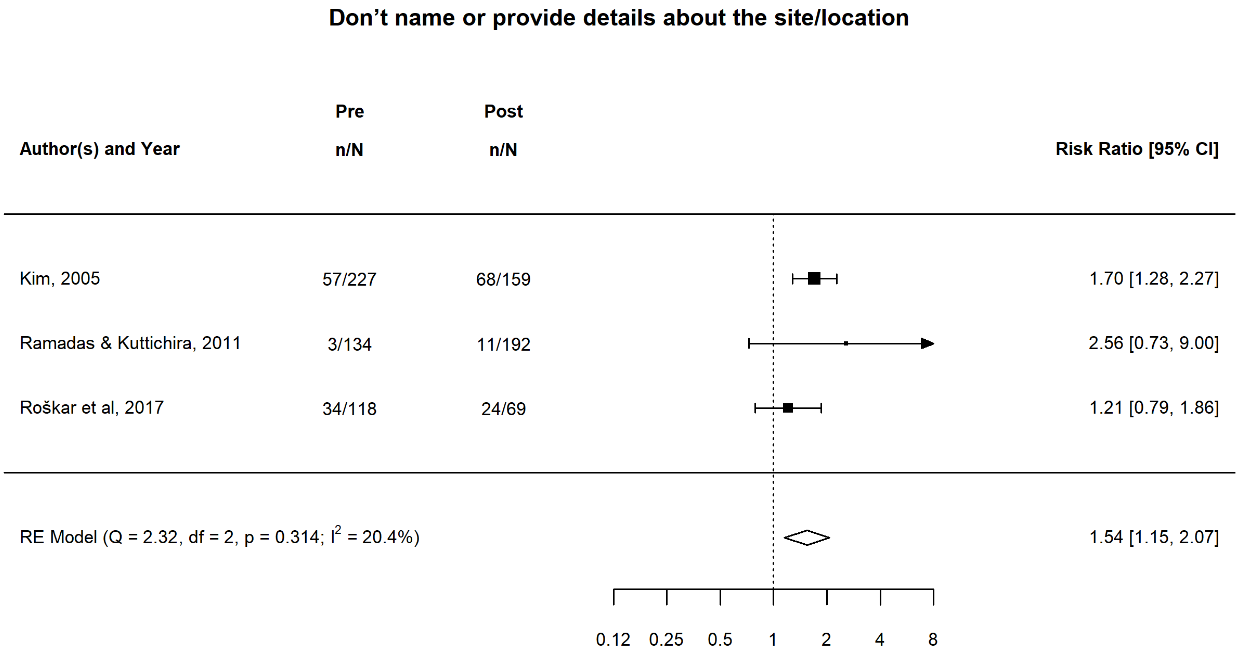

Don’t use language/content which sensationalizes, romanticizes or normalizes suicide, or that presents it as a viable solution to problems

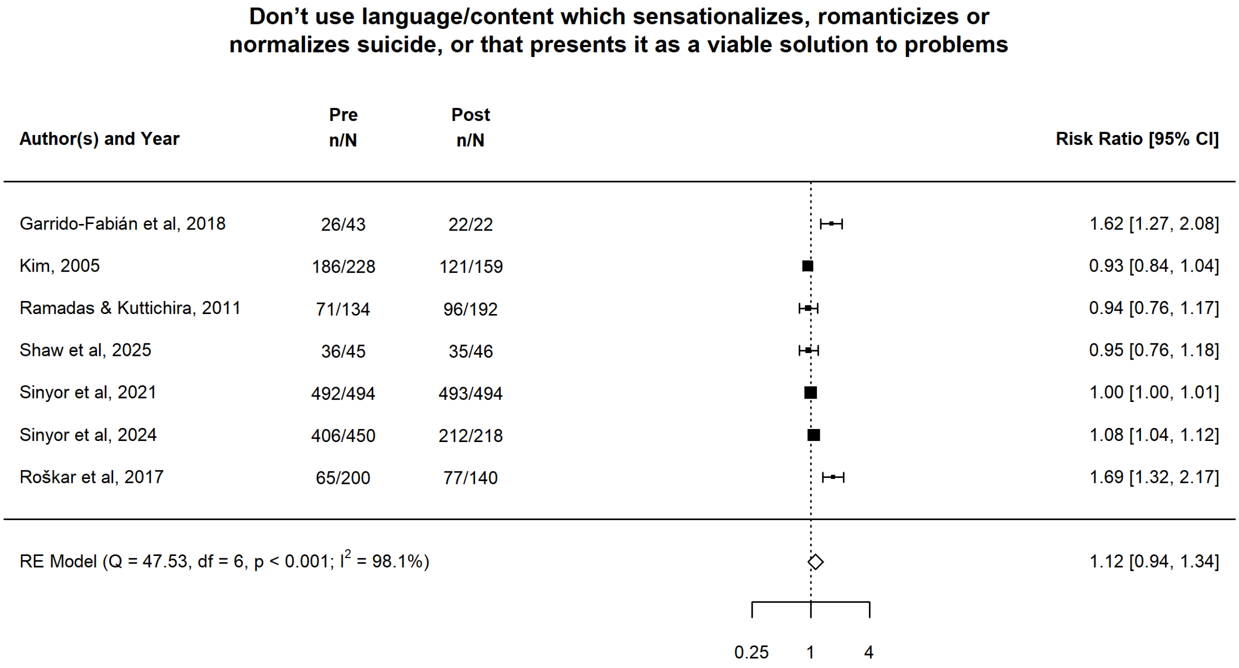

Don’t oversimplify the reason for a suicide or reduce it to a single factor

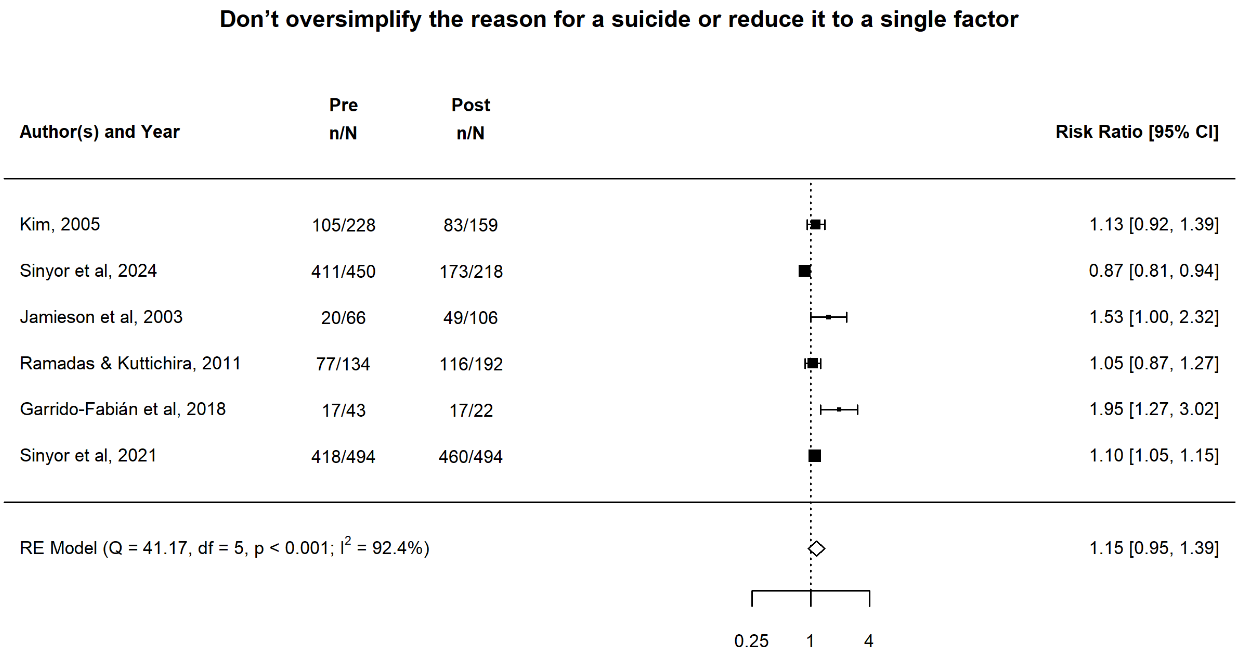

Don’t use sensational language in headlines

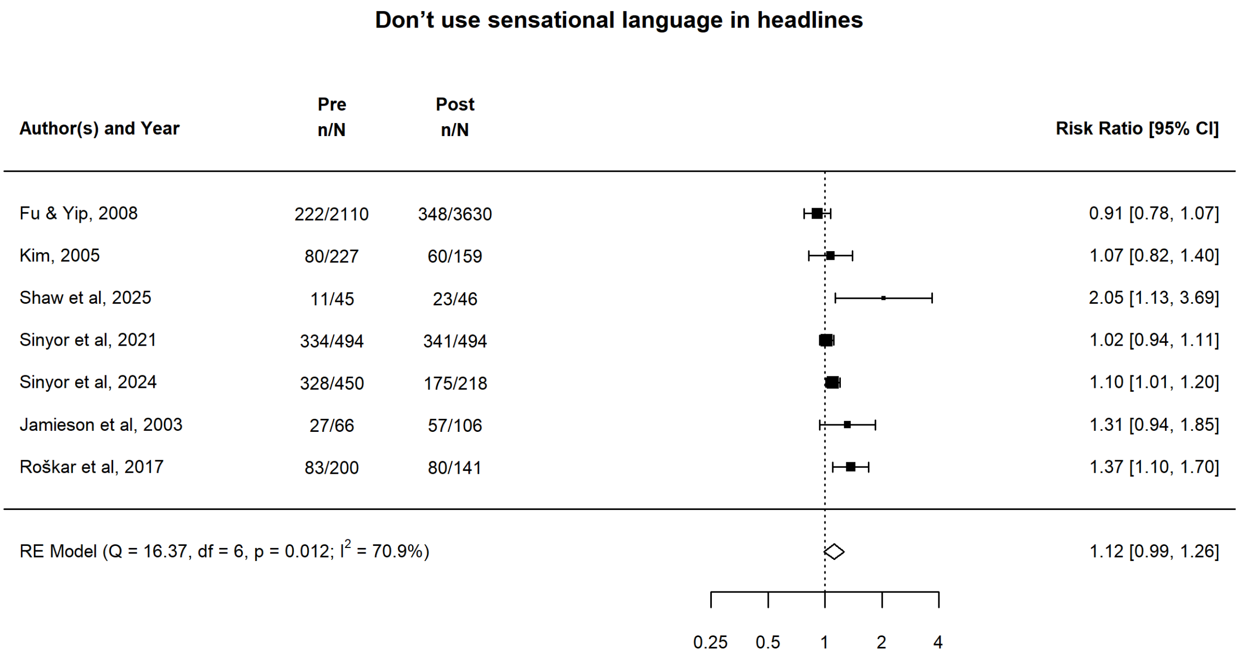

Don’t use photographs, video footage, audio recordings, digital or social media links

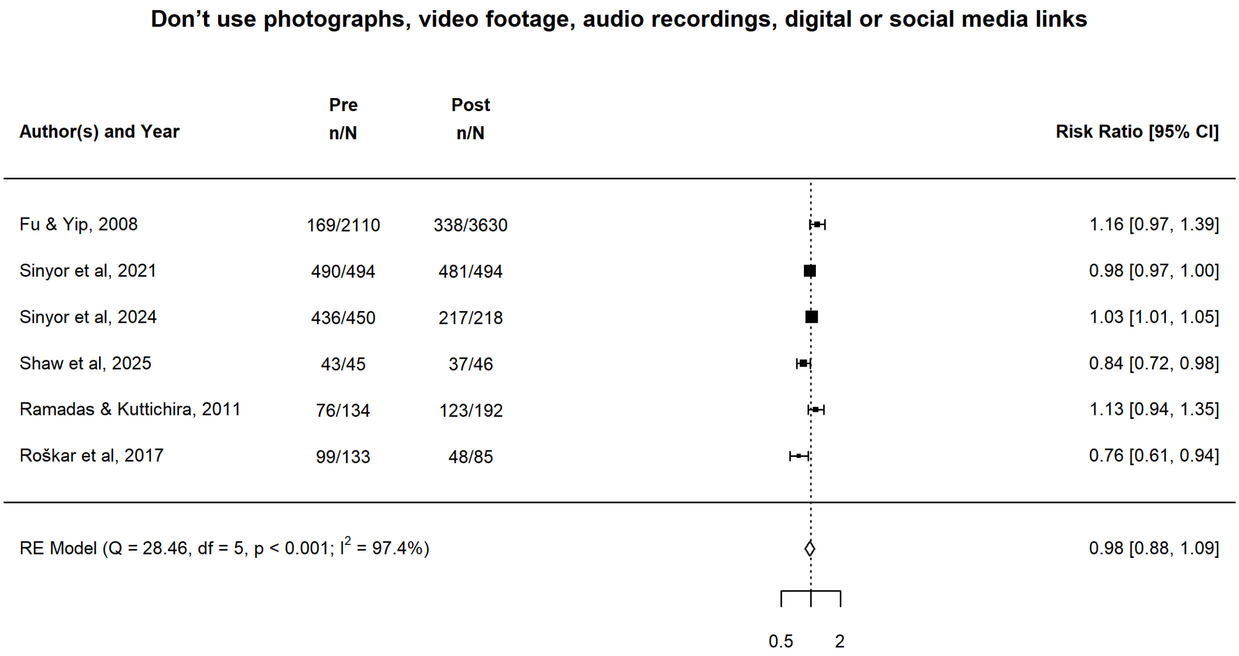

Don’t report the details of a suicide note

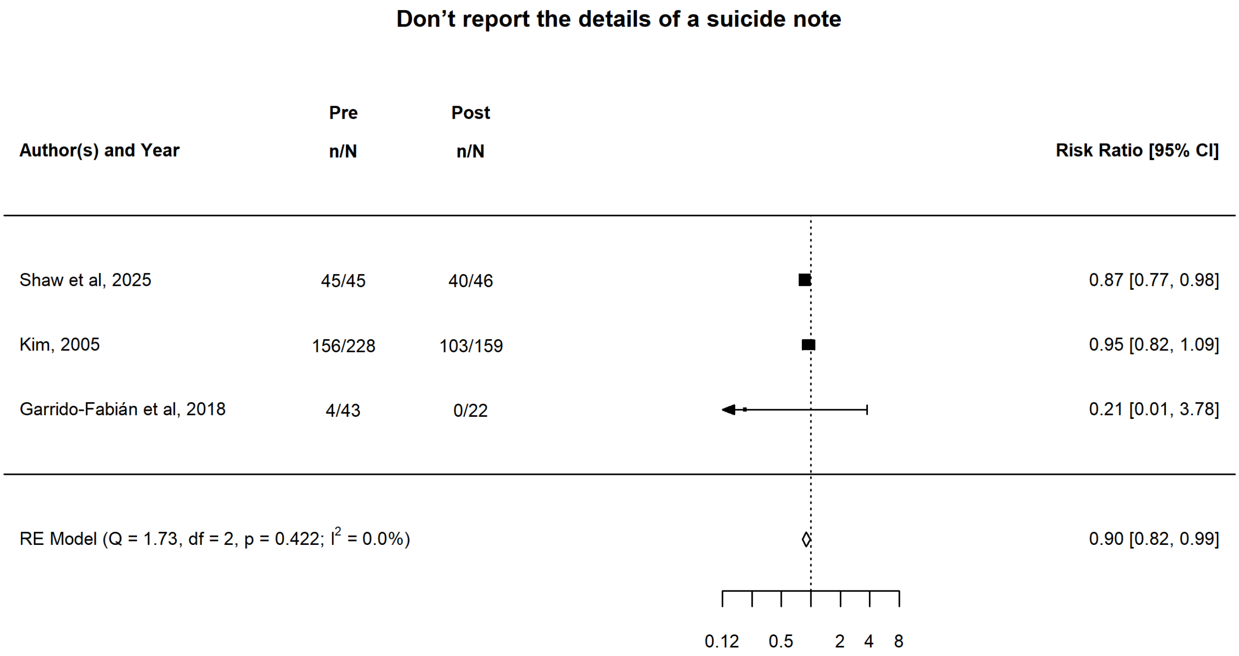

Primary analysis contour-enhanced funnel plots – suicide rates outcome

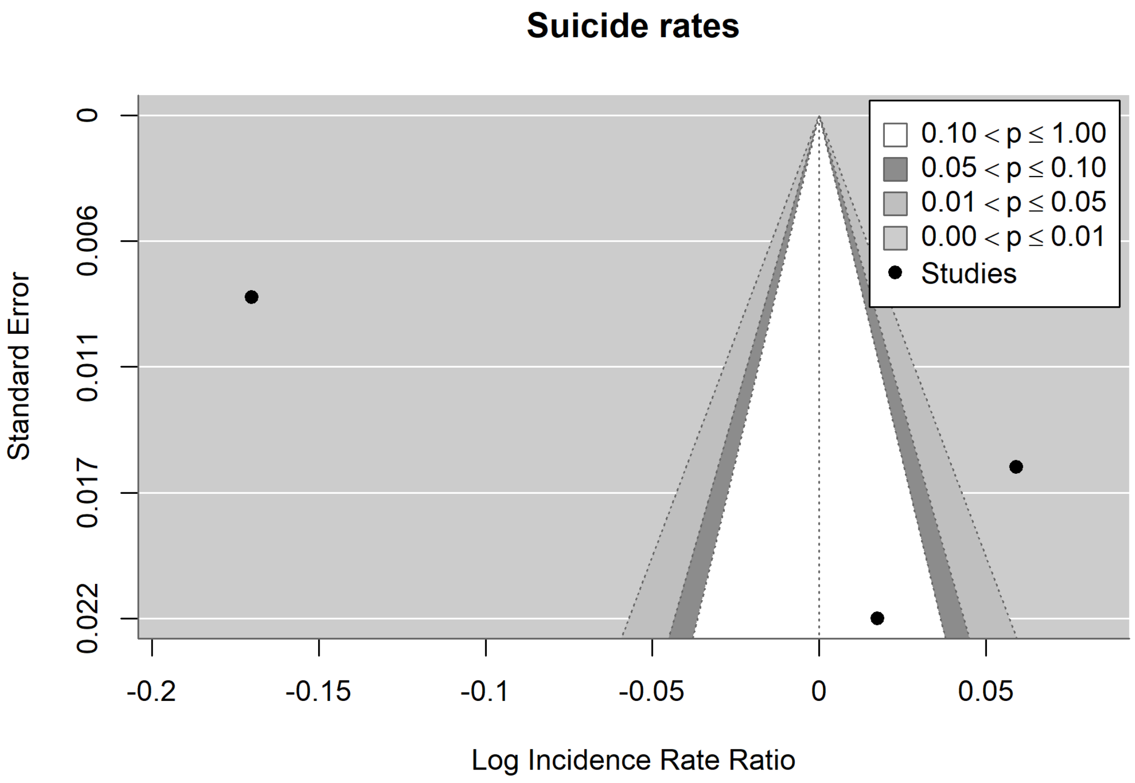

Primary analysis contour-enhanced funnel plots – quality of suicide-related media reporting outcome

Do provide accurate information about where to seek help for suicidal thoughts and suicidal crises

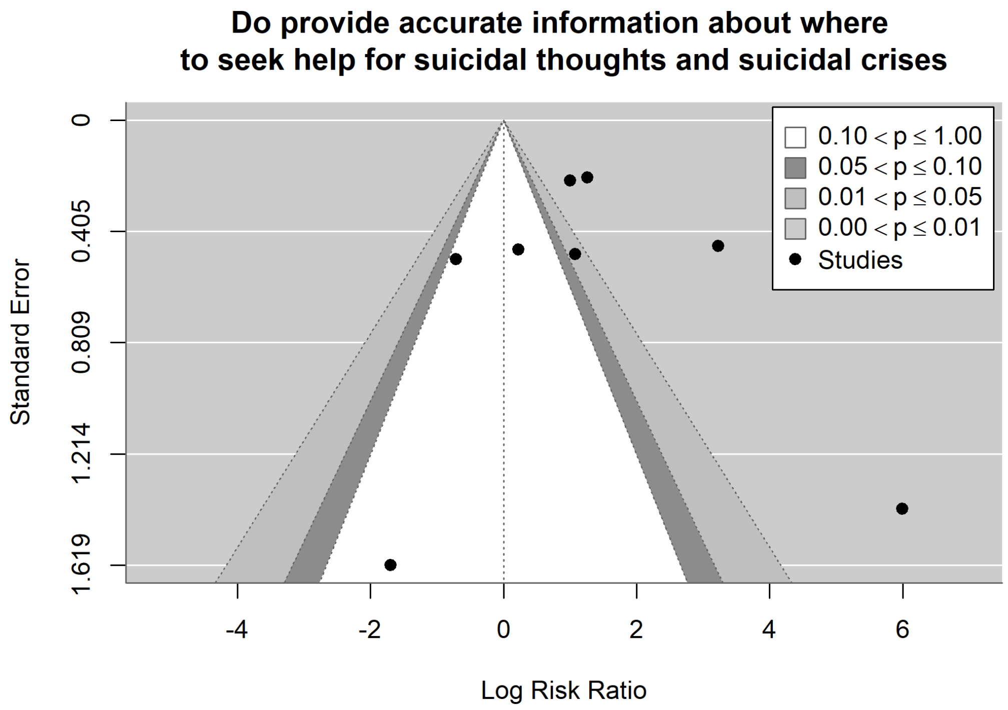

Do educate the public about the facts of suicide and suicide prevention based on accurate information

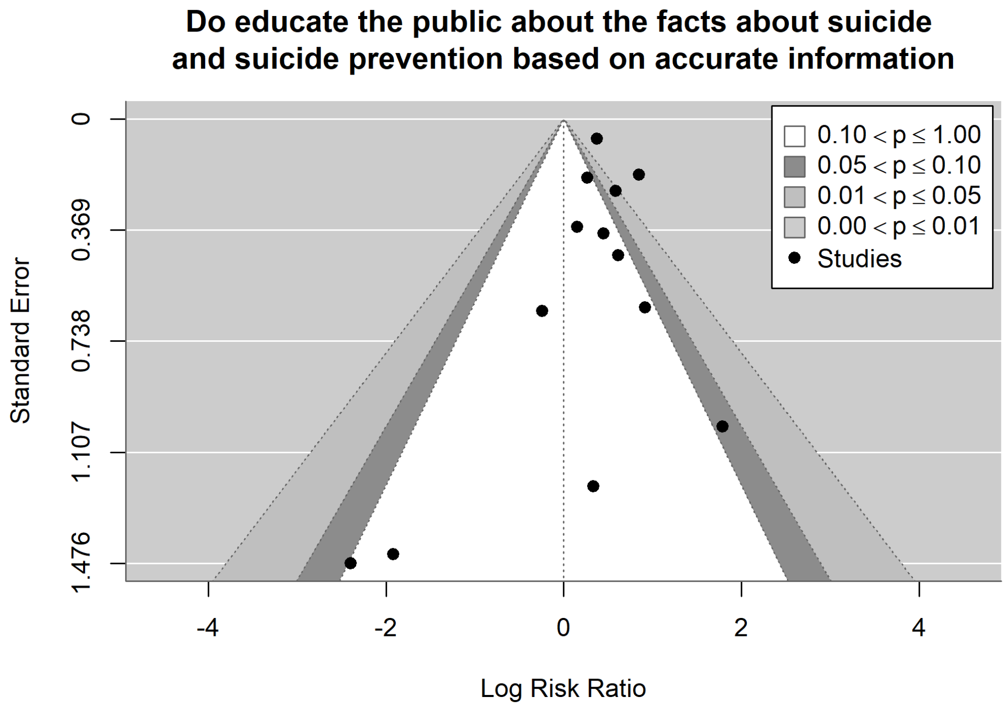

Do report stories of how to cope with life stressors and/or suicidal thoughts and the importance of help-seeking

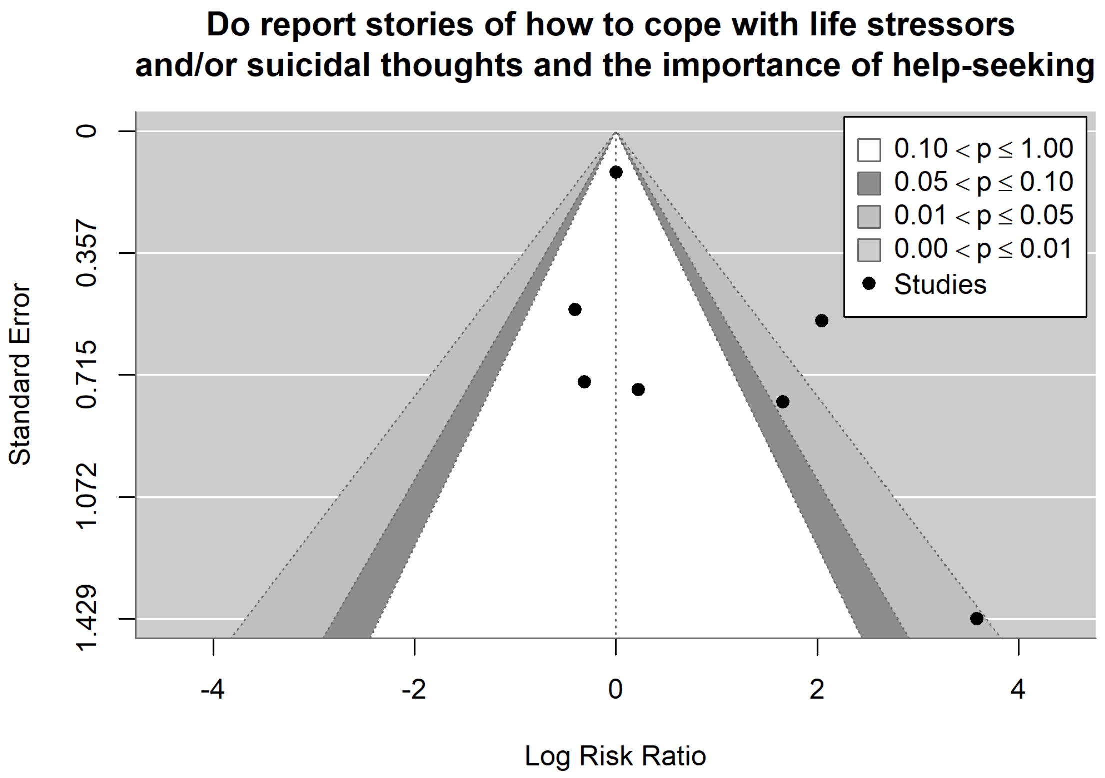

Do apply particular caution when reporting celebrity suicides

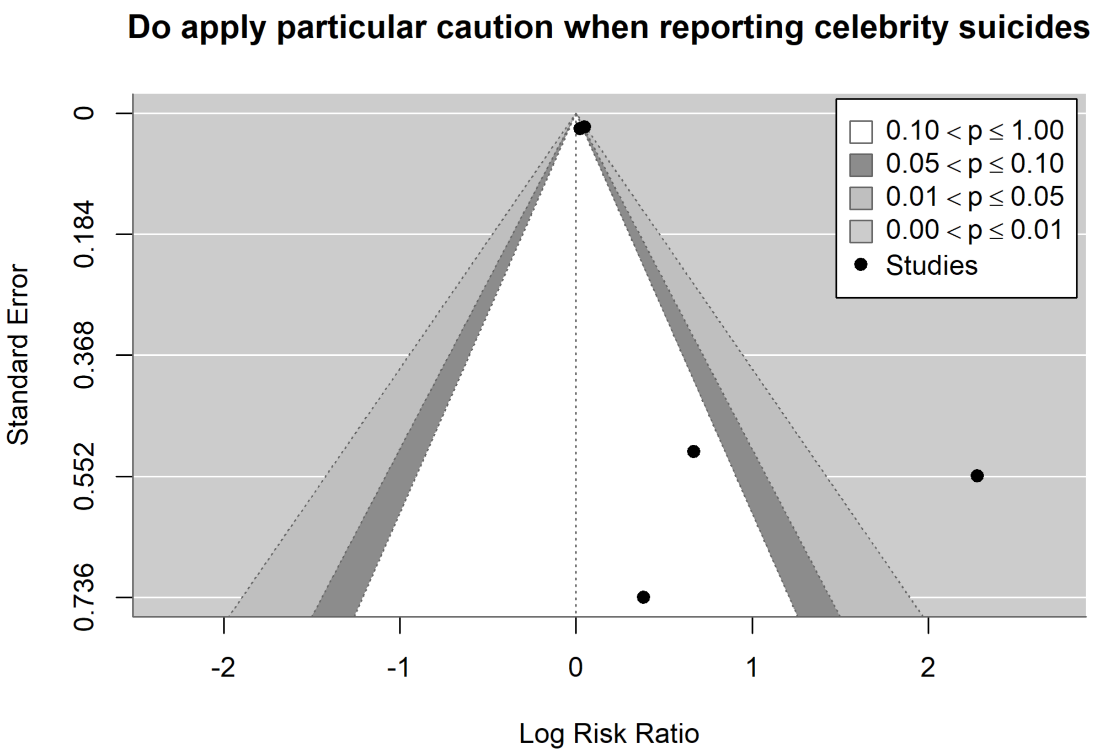

Do apply caution when interviewing bereaved family or friends or persons with lived experience

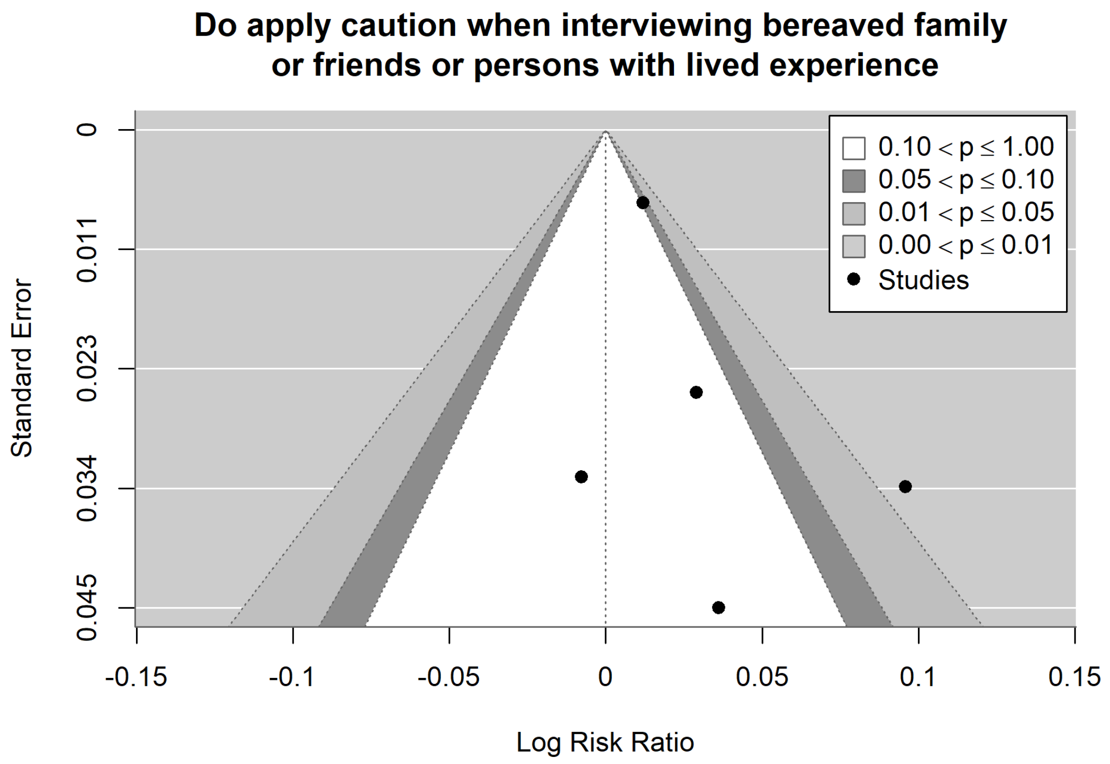

Don’t position suicide-related content as the top story and don’t unduly repeat such stories

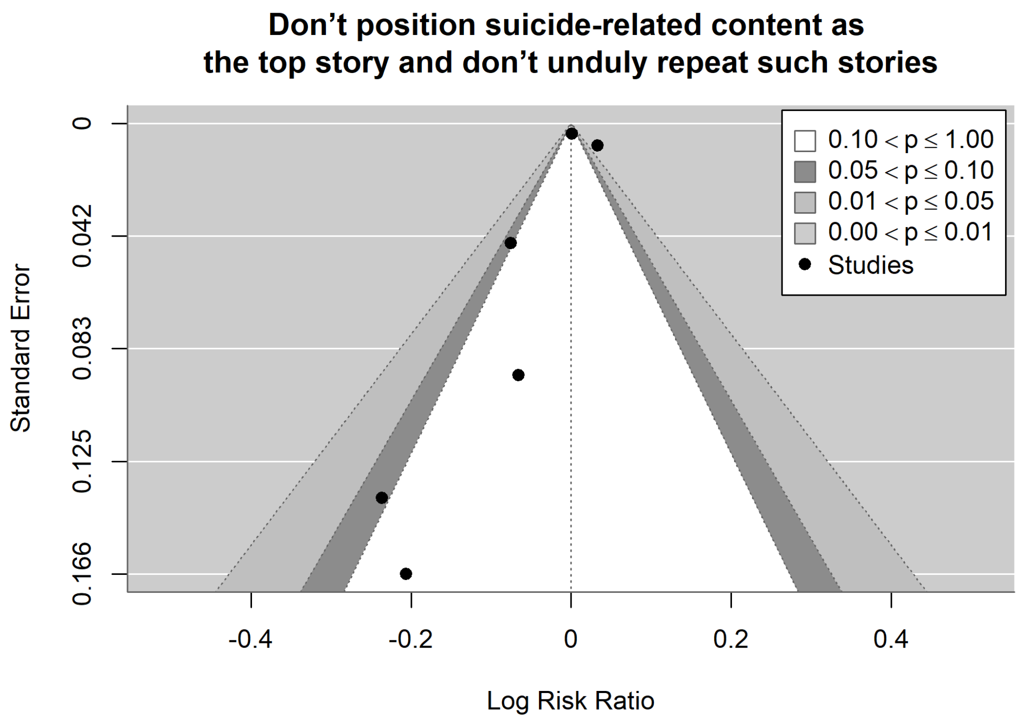

Don’t describe the method used

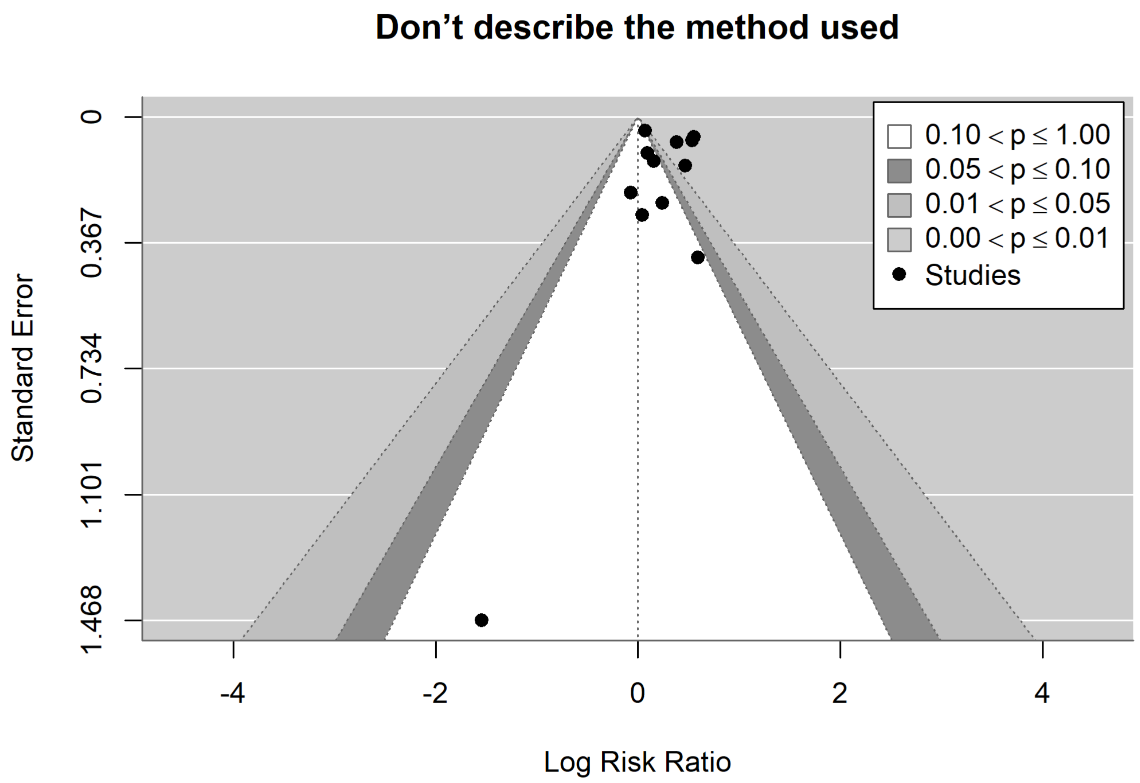

Don’t name or provide details about the site/location

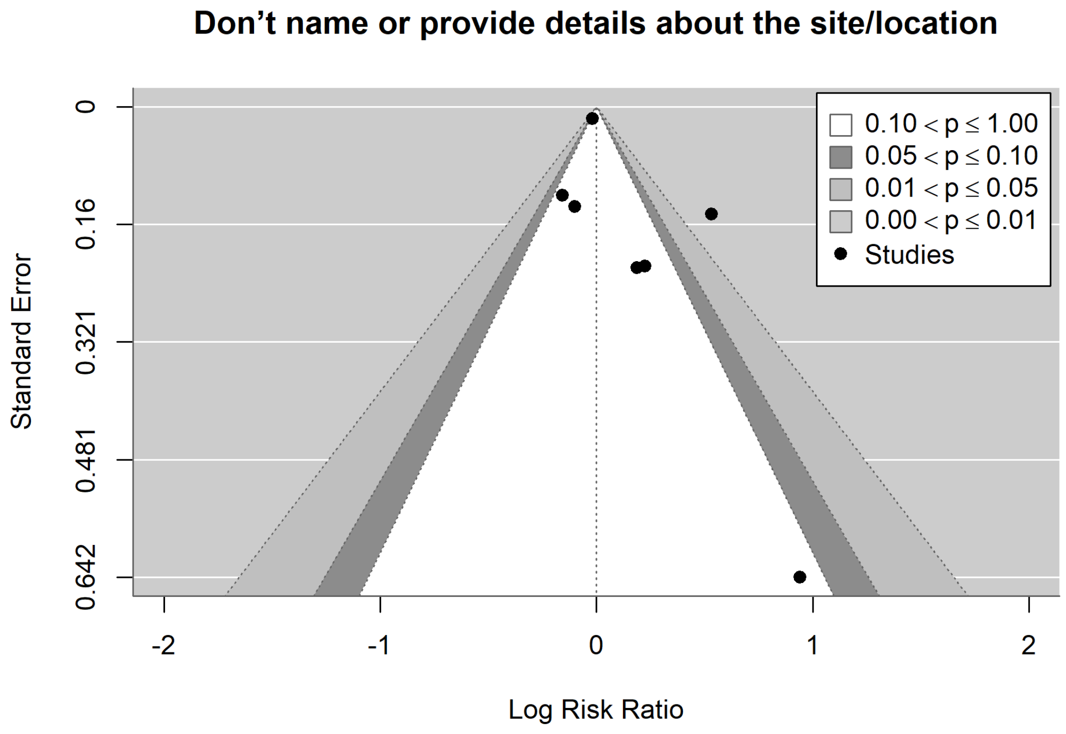

Don’t use language/content which sensationalizes, romanticizes or normalizes suicide, or that presents it as a viable solution to problems

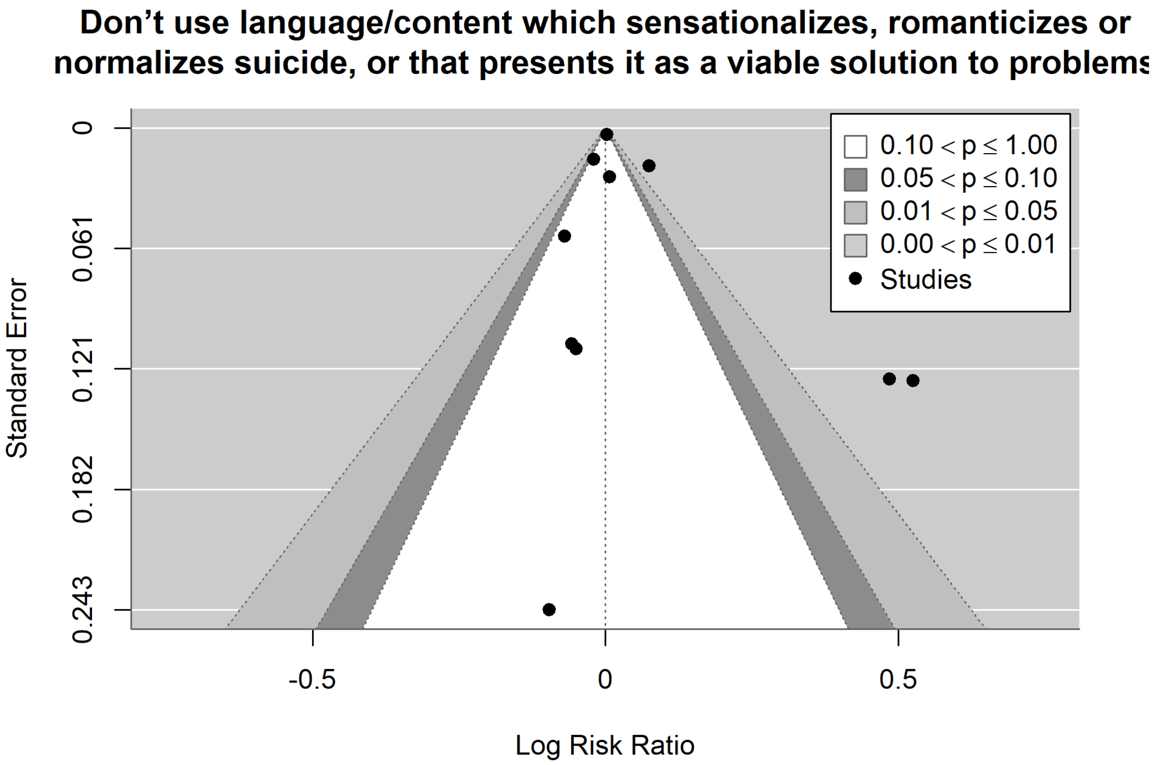

Don’t oversimplify the reason for a suicide or reduce it to a single factor

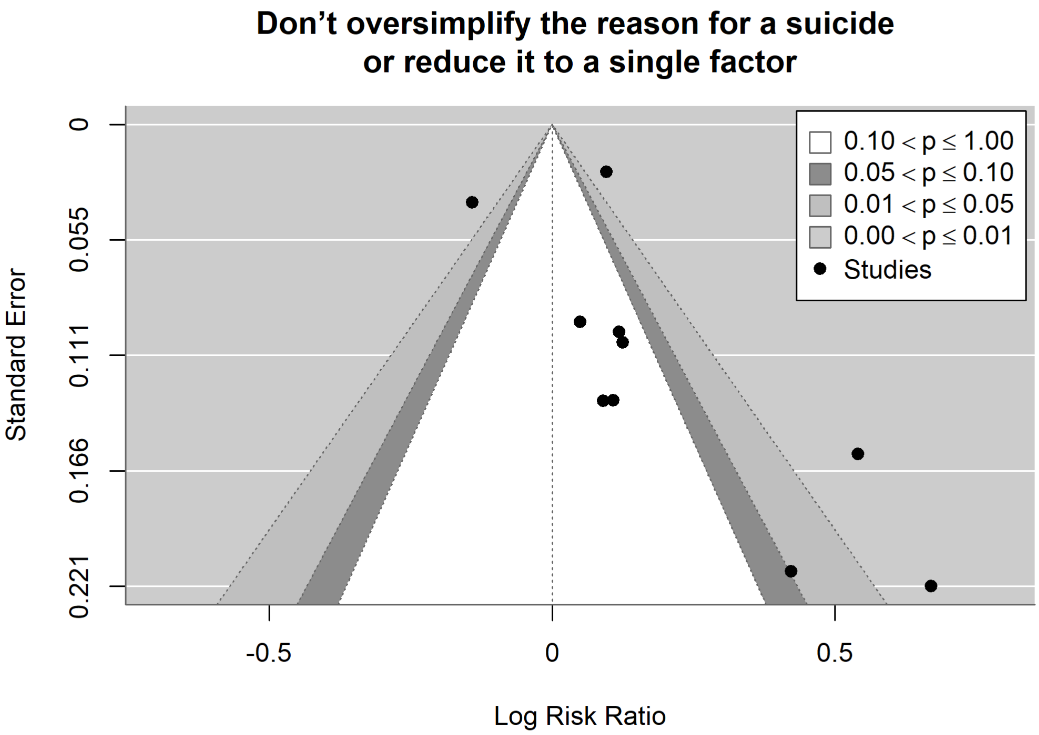

Don’t use sensational language in headlines

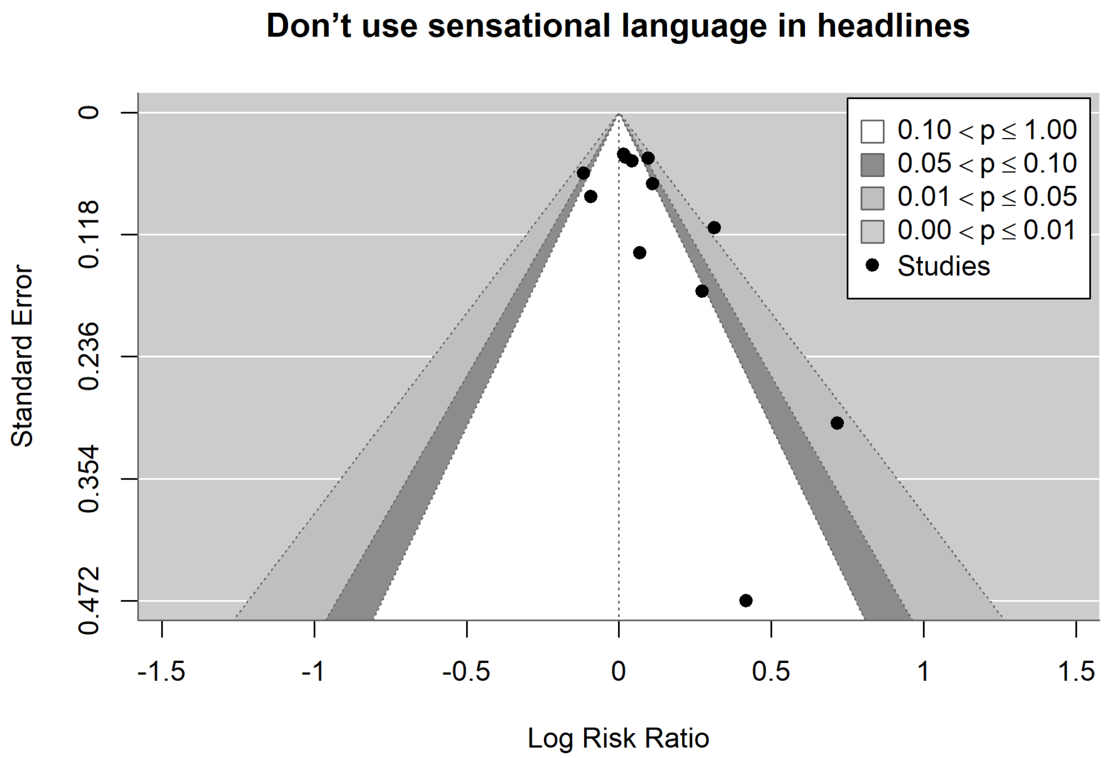

Don’t use photographs, video footage, audio recordings, digital or social media links

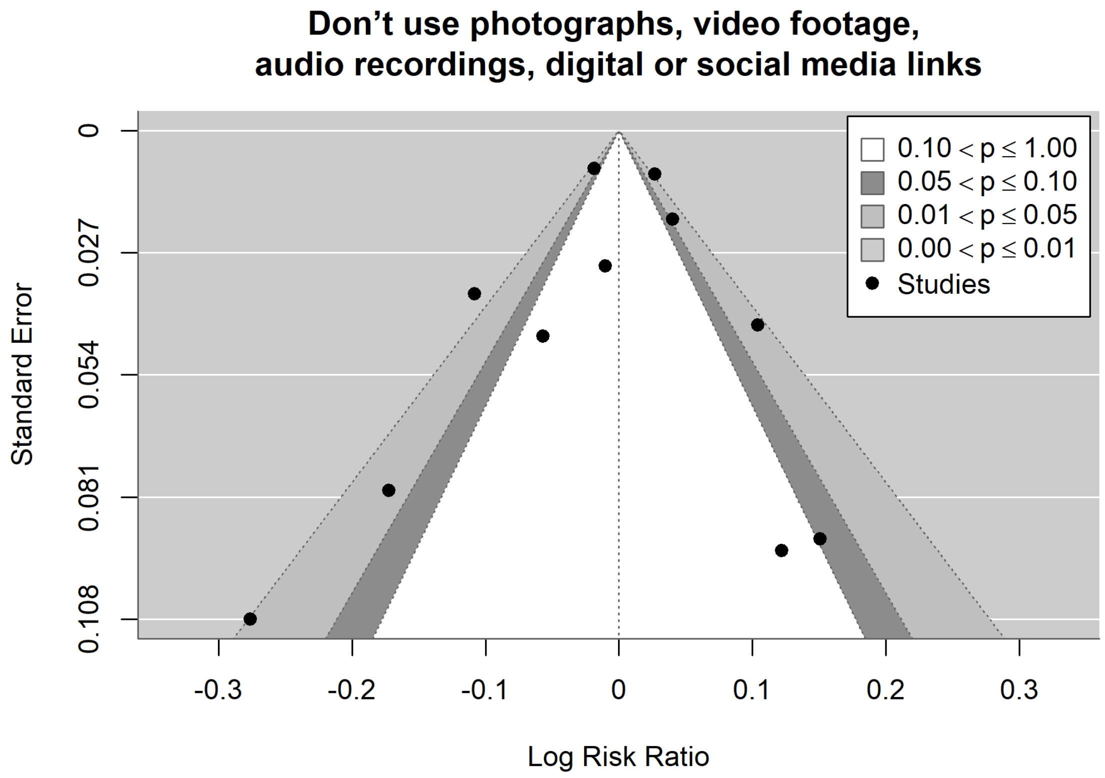

Don’t report the details of a suicide note

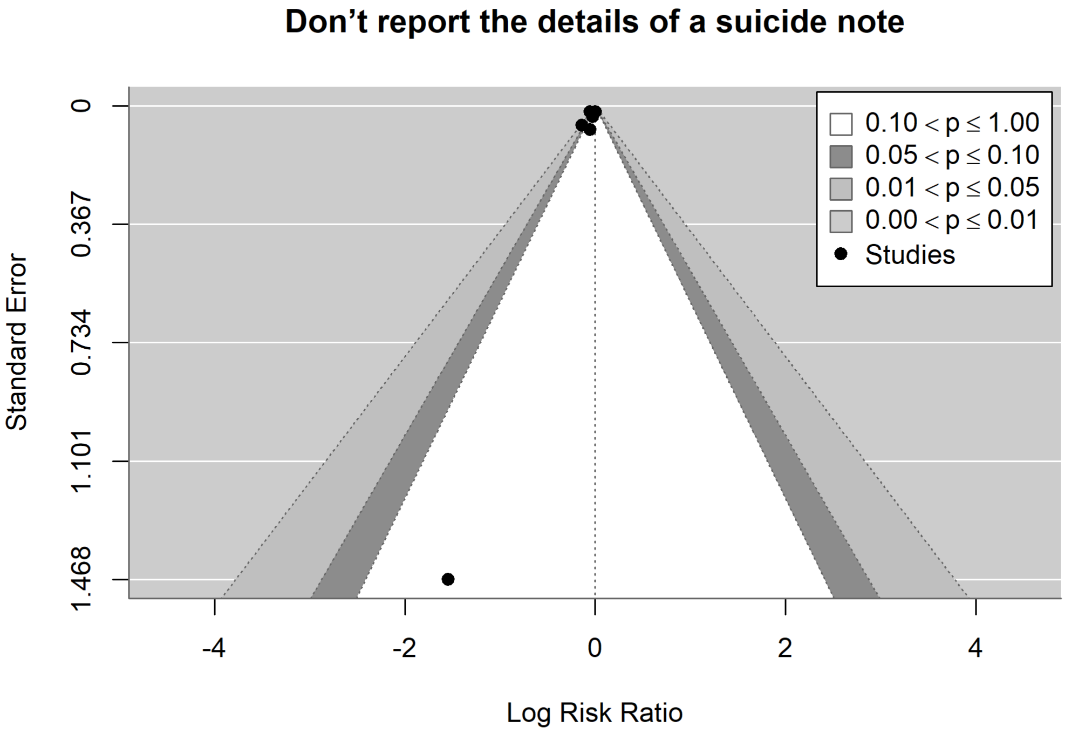

Sensitivity analysis contour-enhanced funnel plots – suicide outcome

Studies with no gap between guideline release and the start of the post-intervention assessment period

Note that the three studies that assessed suicide as an outcome (Niederkrotenthaler & Sonneck, 2007; Sinyor et al., 2021; Sinyor et al., 2024) had no gap between the implementation of guidelines and the start of the post-intervention assessment period. Therefore, the funnel plot here is the same as its primary analysis funnel plot.

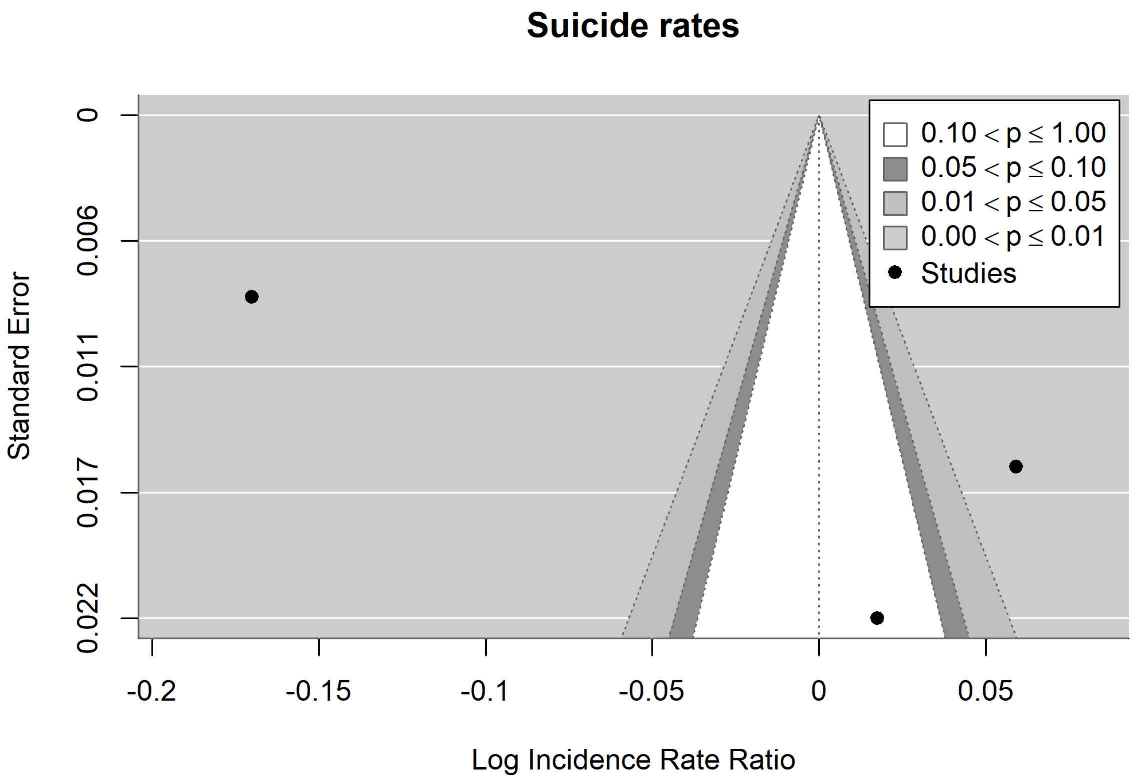

Sensitivity analysis contour-enhanced funnel plots – quality of suicide-related media reporting outcome

Studies with a gap between guideline release and the start of the post-intervention assessment period

Note that the five studies (Armstrong et al., 2025; Eriyanto, 2025; Acosta et al., 2020; Ju et al., 2024; Pirkis et al., 2009) with a gap between the implementation of guidelines and the start of the post-intervention assessment period measured some but not all WHO/IASP (2023) recommendations.

Do provide accurate information about where to seek help for suicidal thoughts and suicidal crises

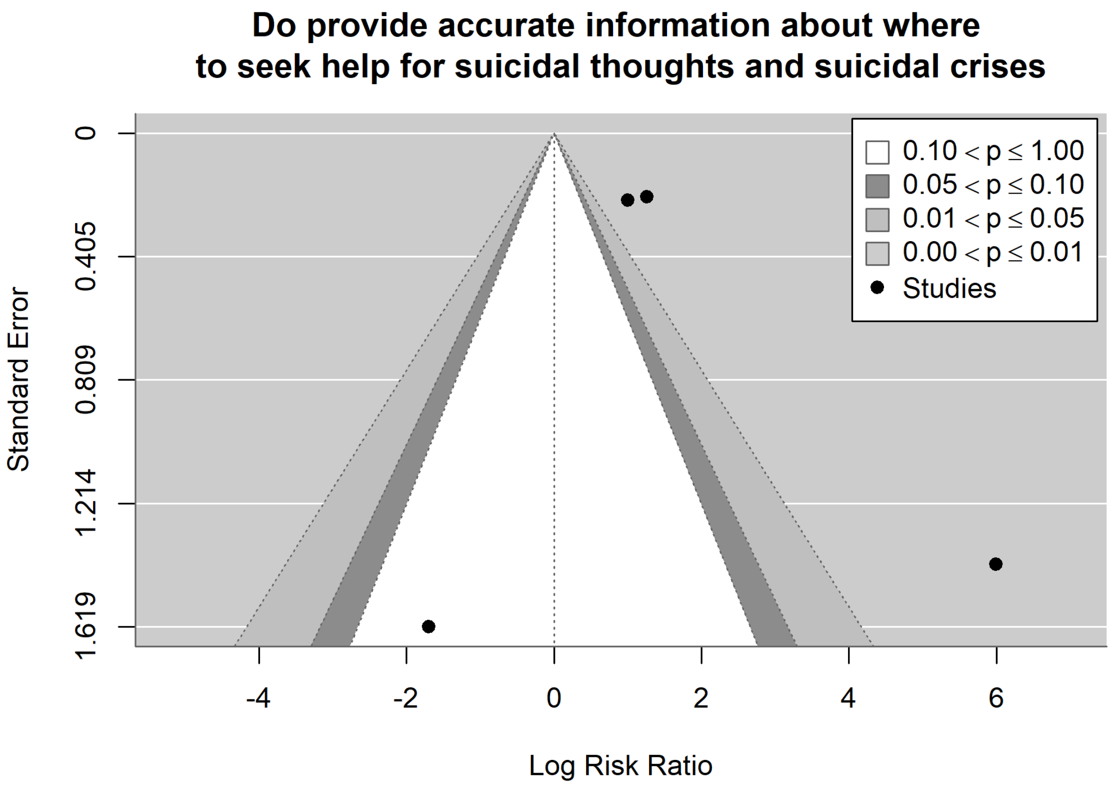

Do educate the public about the facts of suicide and suicide prevention based on accurate information

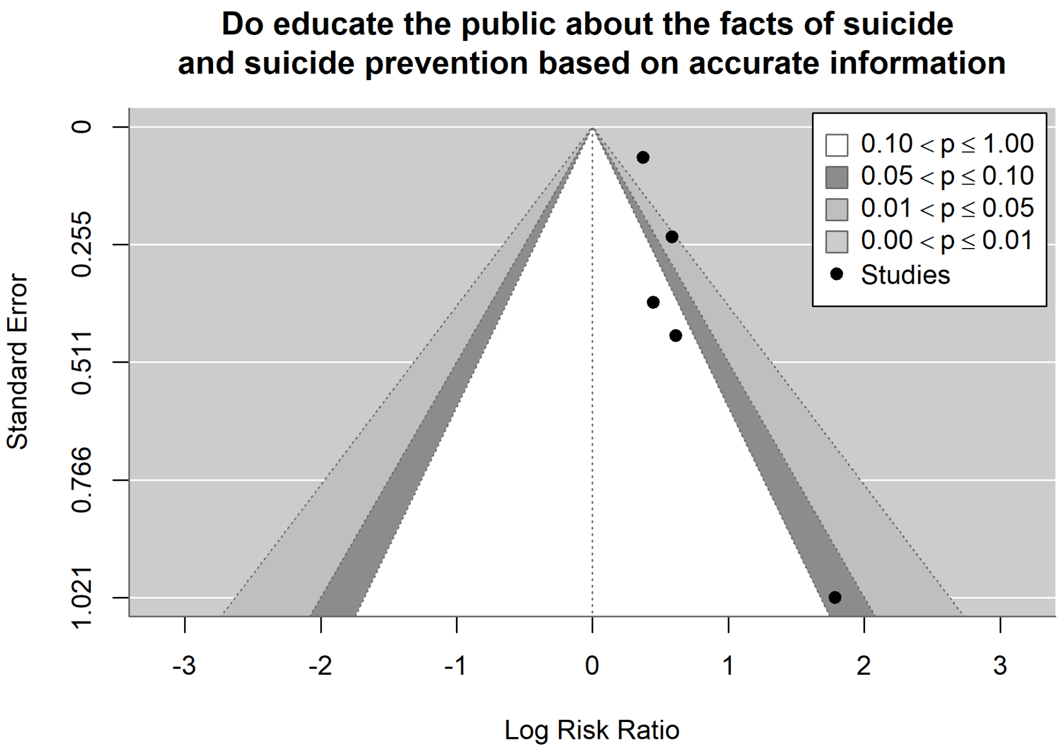

Do report stories of how to cope with life stressors and/or suicidal thoughts and the importance of help-seeking

No studies with a gap between guideline release and the start of the post-intervention assessment period assessed this recommendation.

Do apply particular caution when reporting celebrity suicides

No studies with a gap between guideline release and the start of the post-intervention assessment period assessed this recommendation.

Do apply caution when interviewing bereaved family or friends or persons with lived experience

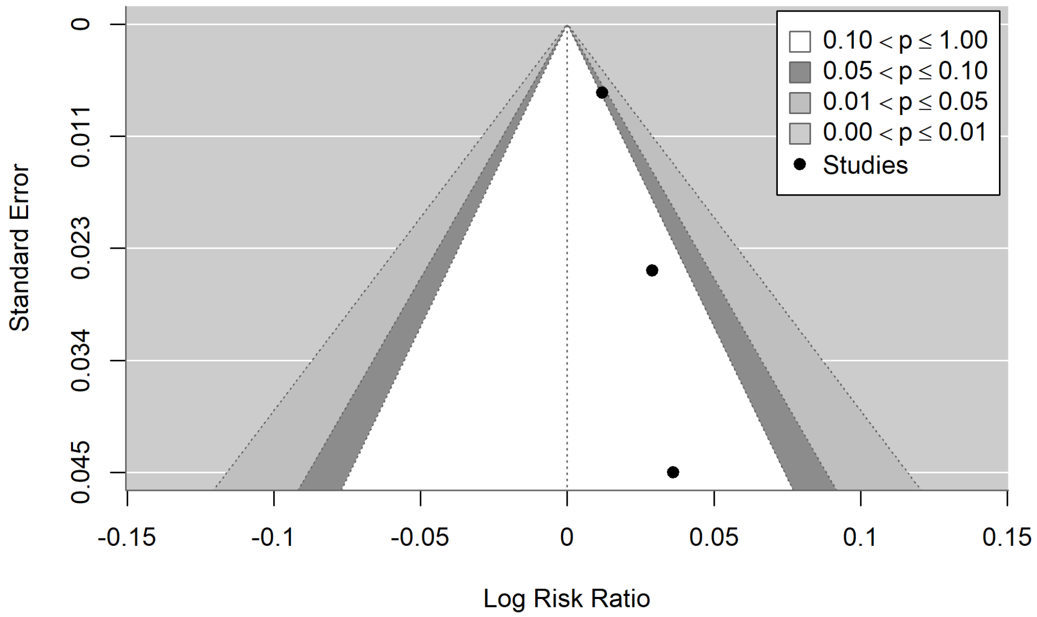

Don’t position suicide-related content as the top story and don’t unduly repeat such stories

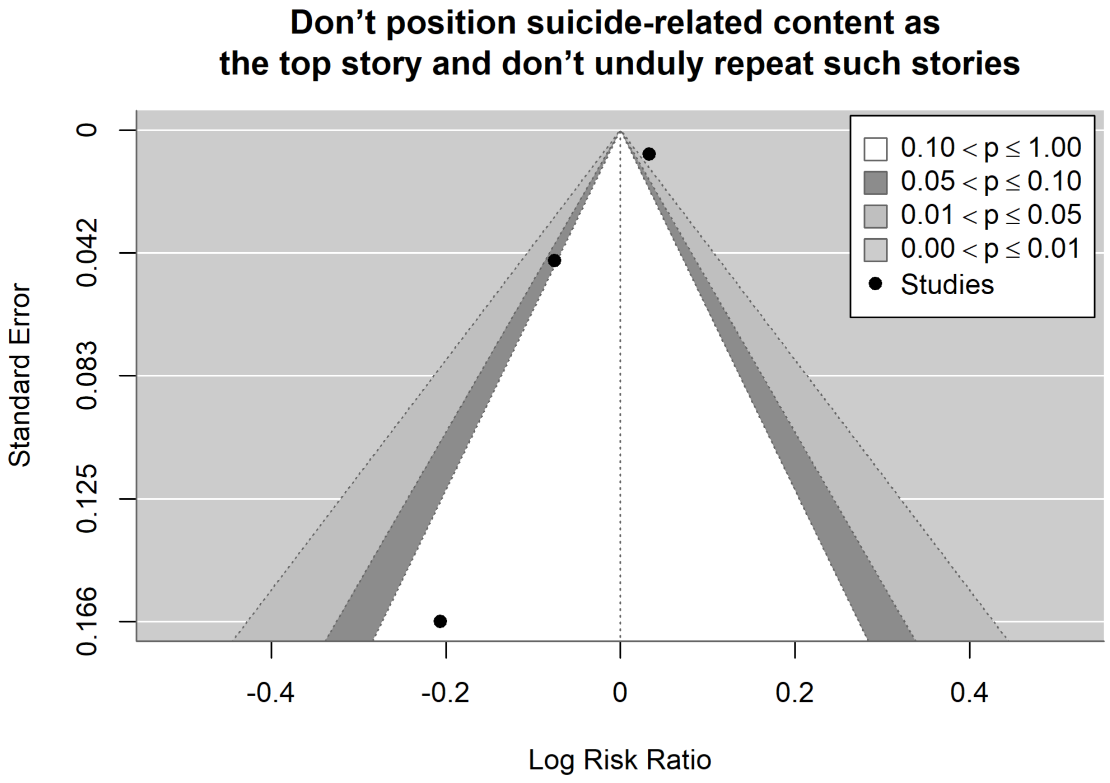

Don’t describe the method used

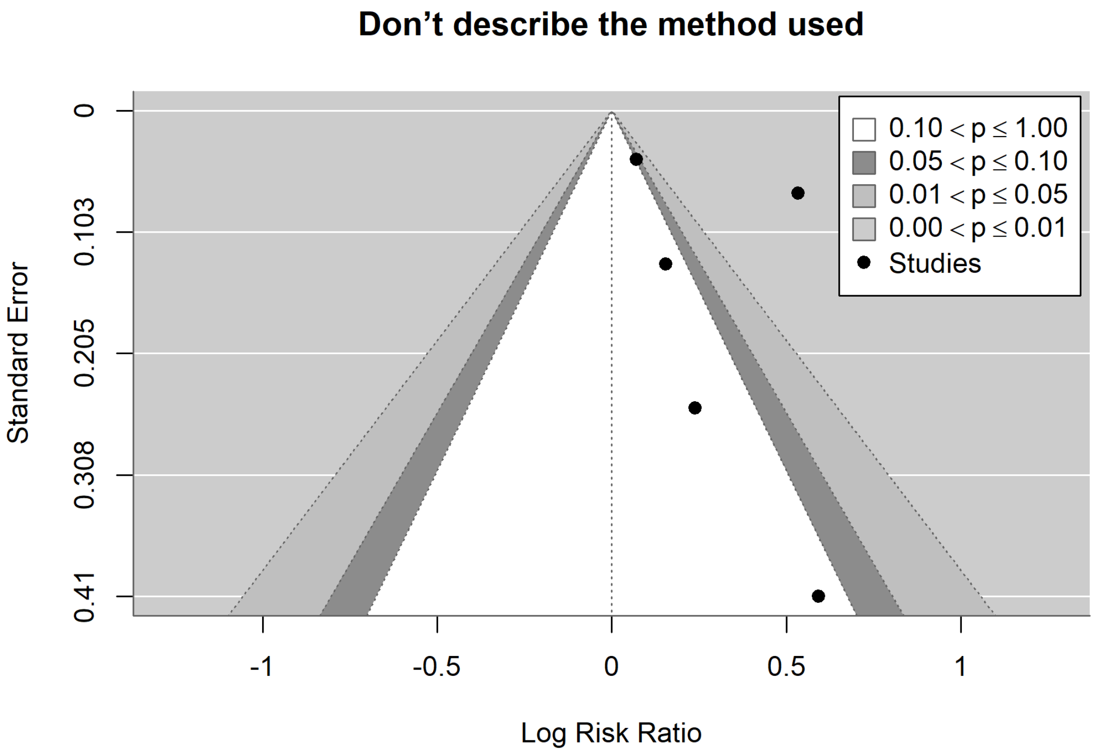

Don't name or provide details about the site/location

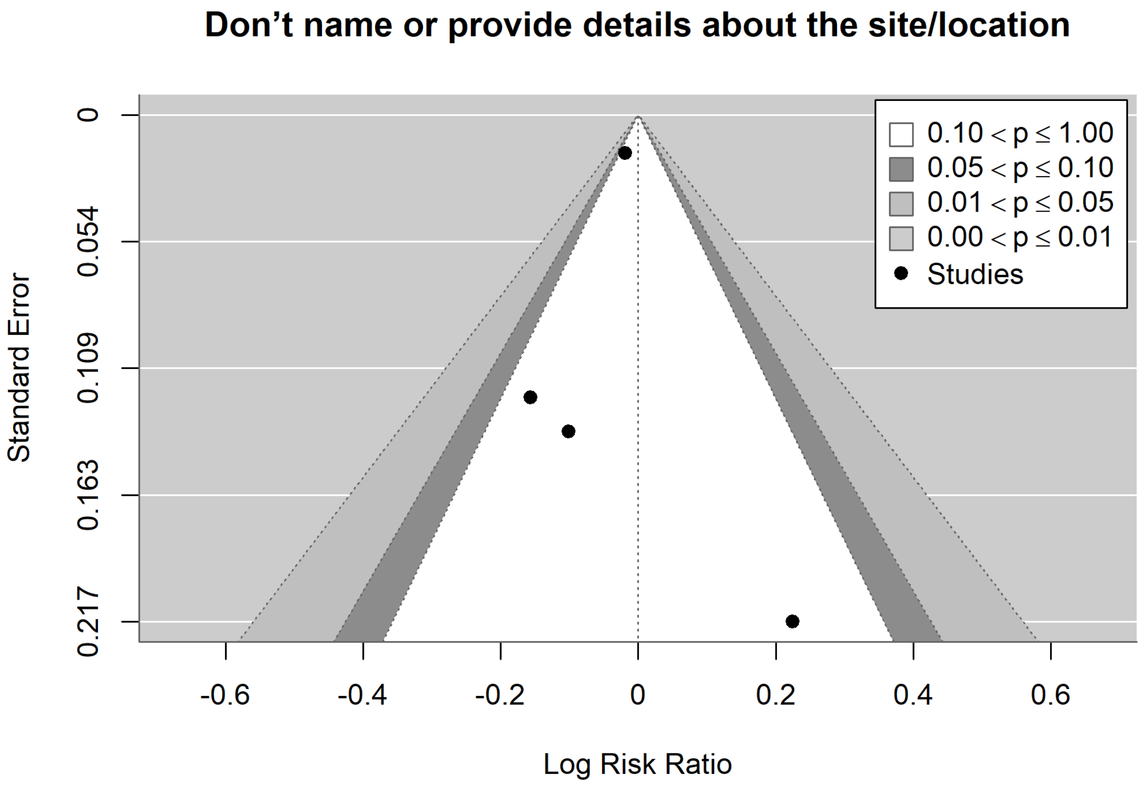

Don't use language/content which sensationalizes, romanticizes or normalizes suicide, or that presents it as a viable solution to problems

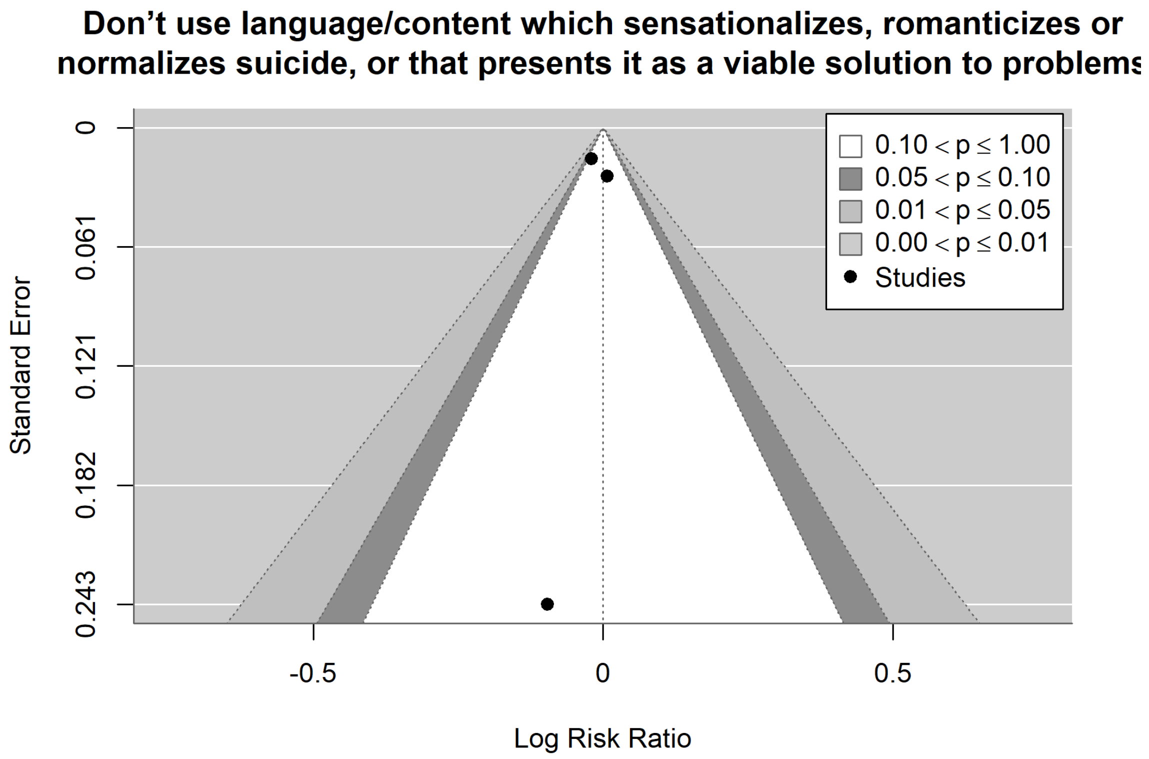

Don't oversimplify the reason for a suicide or reduce it to a single factor

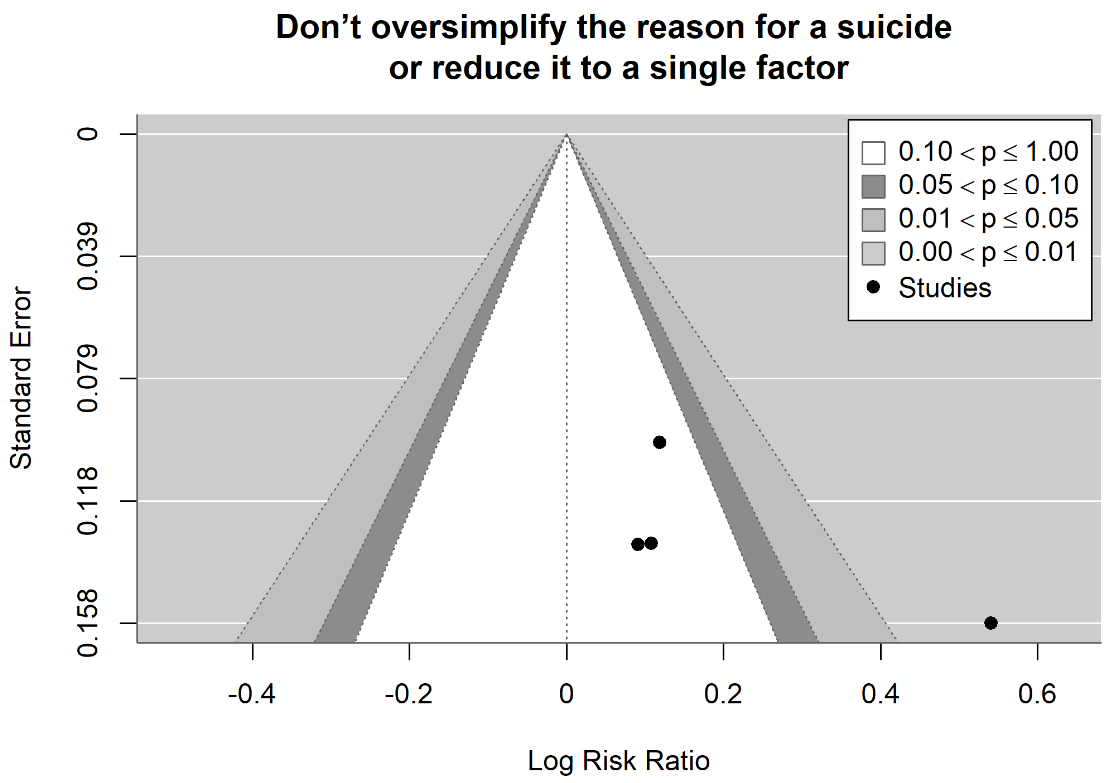

Don't use sensational language in headlines

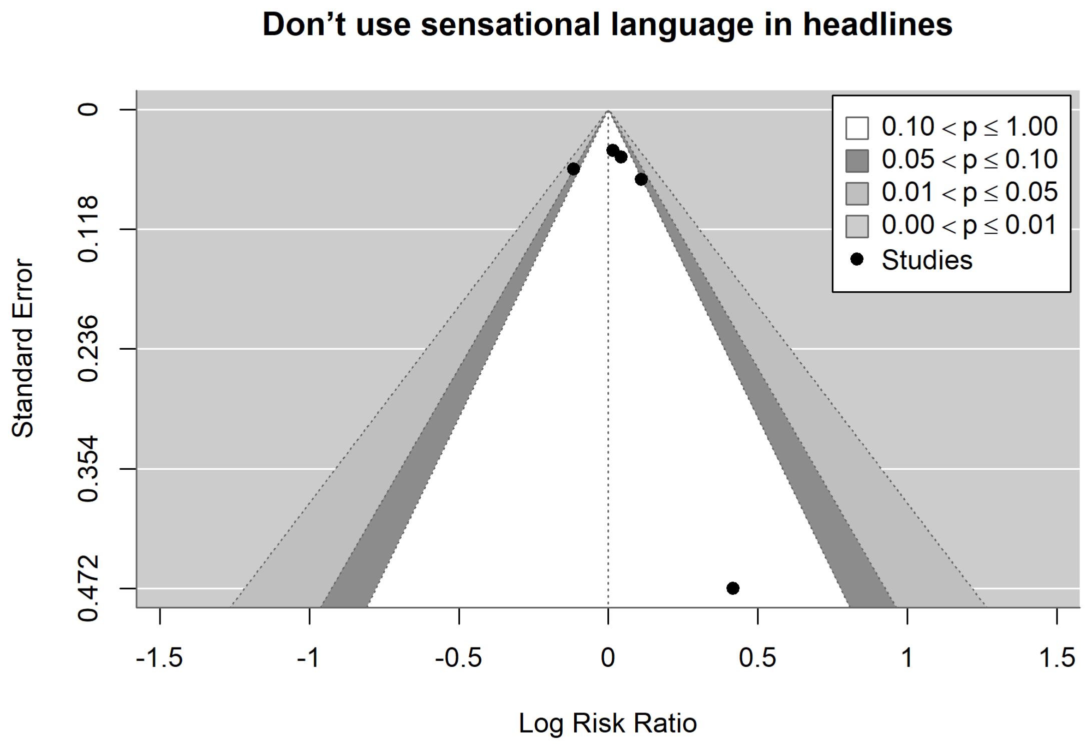

Don't use photographs, video footage, audio recordings, digital or social media links

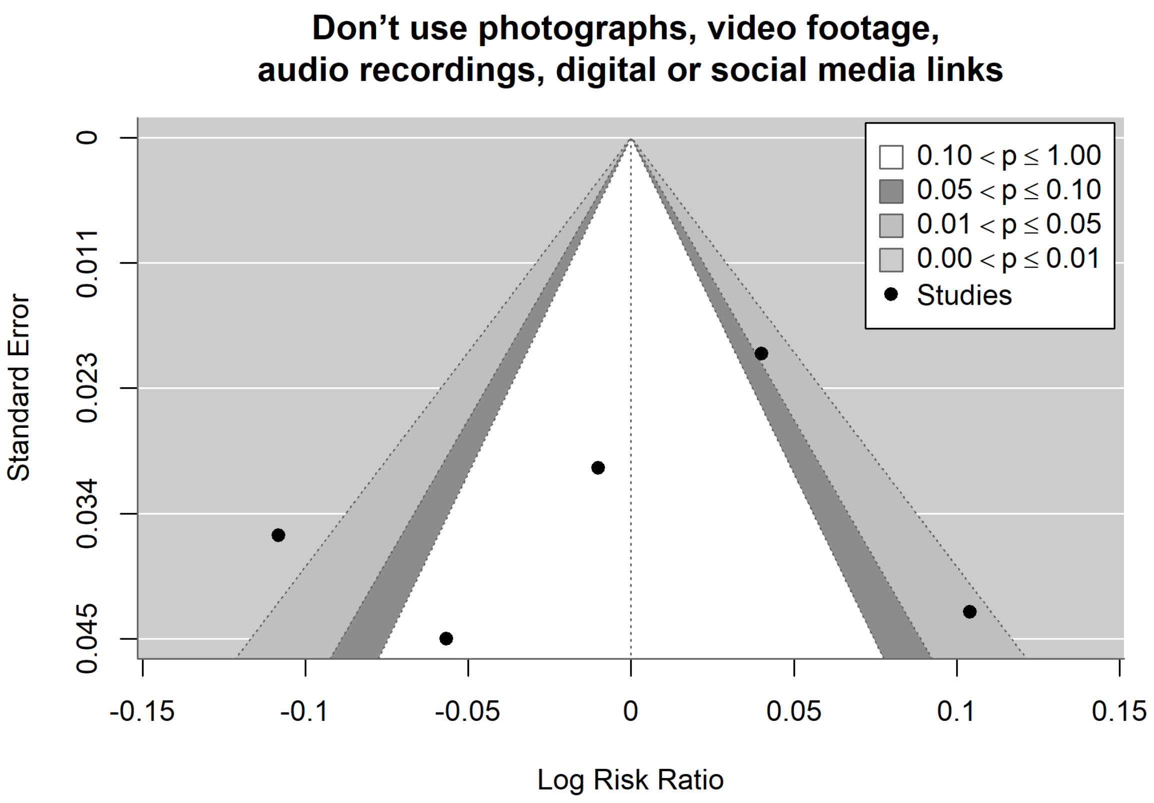

Don't report the details of a suicide note

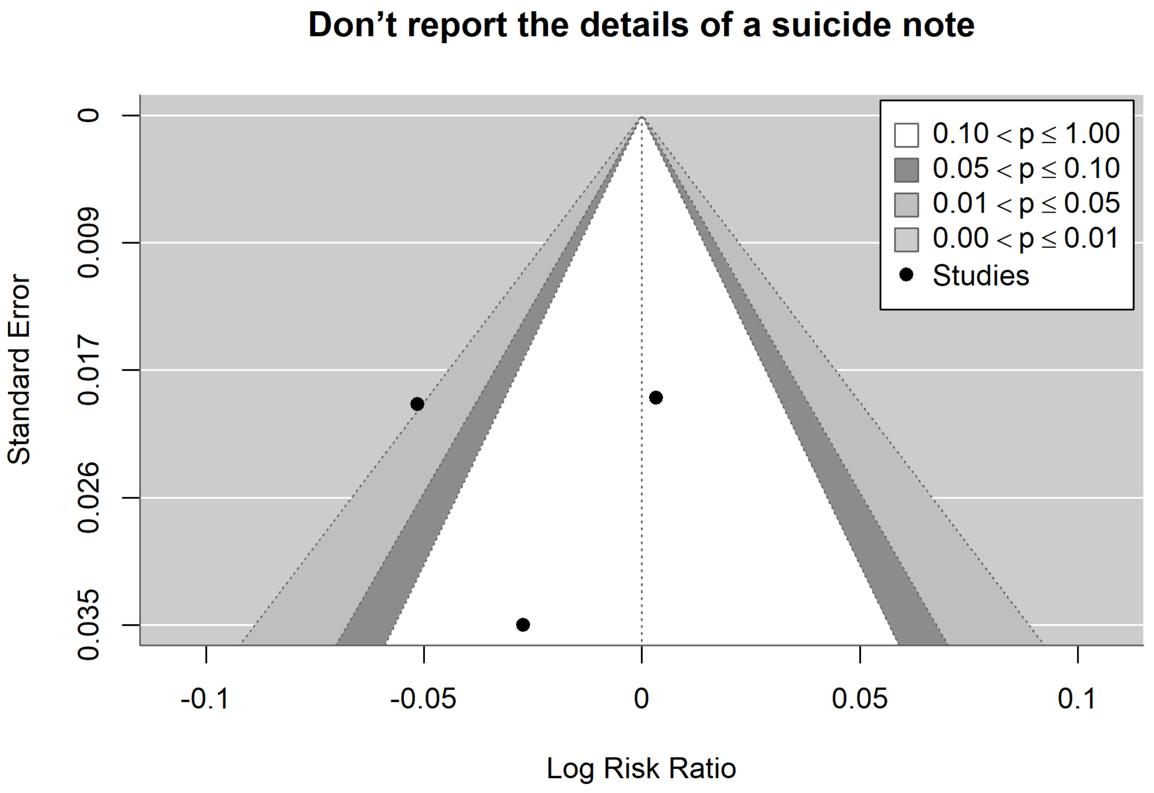

Studies with no gap between guideline release and the start of the post-intervention assessment period

Note that the remaining eight studies (Shaw et al. 2025; Fu & Yip, 2008; Garrido-Fabián et al., 2018; Jamieson et al., 2003; Kim 2005; Ramadas & Kuttichira, 2011; Roškar et al., 2017; Sinyor et al., 2021; Sinyor et al., 2024) with no gap between the implementation of guidelines and the start of the post-intervention assessment period measured some but not all WHO/IASP (2023) recommendations.

Do provide accurate information about where to seek help for suicidal thoughts and suicidal crises

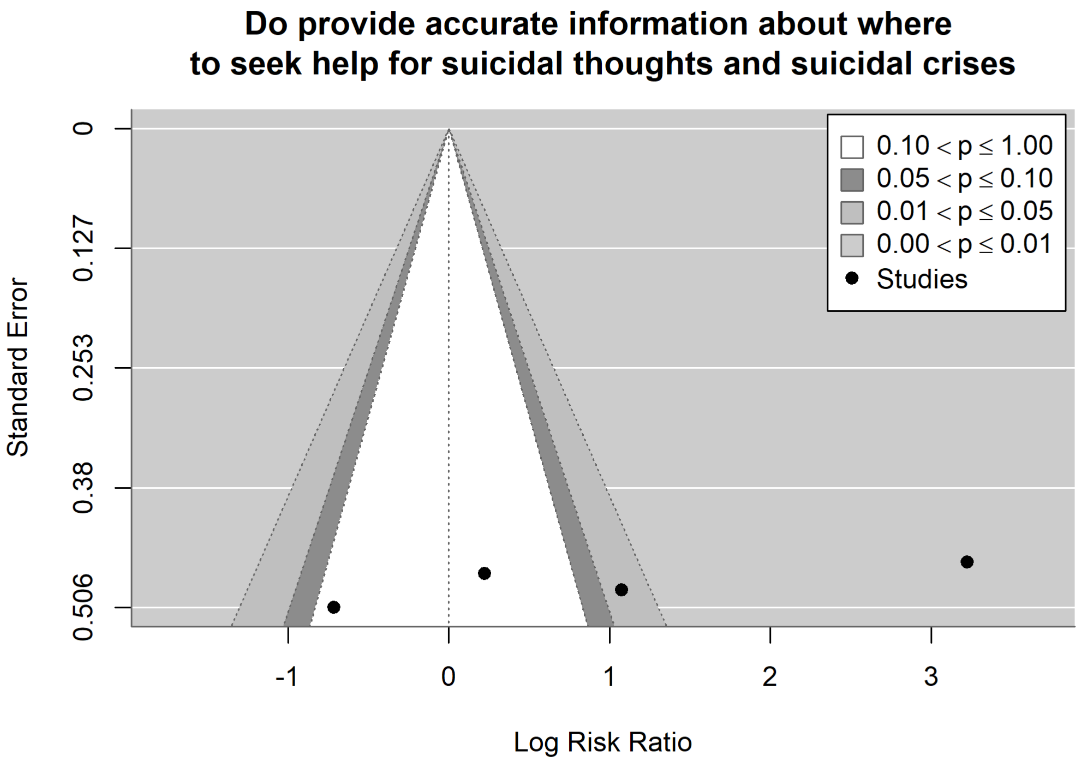

Do educate the public about the facts of suicide and suicide prevention based on accurate information

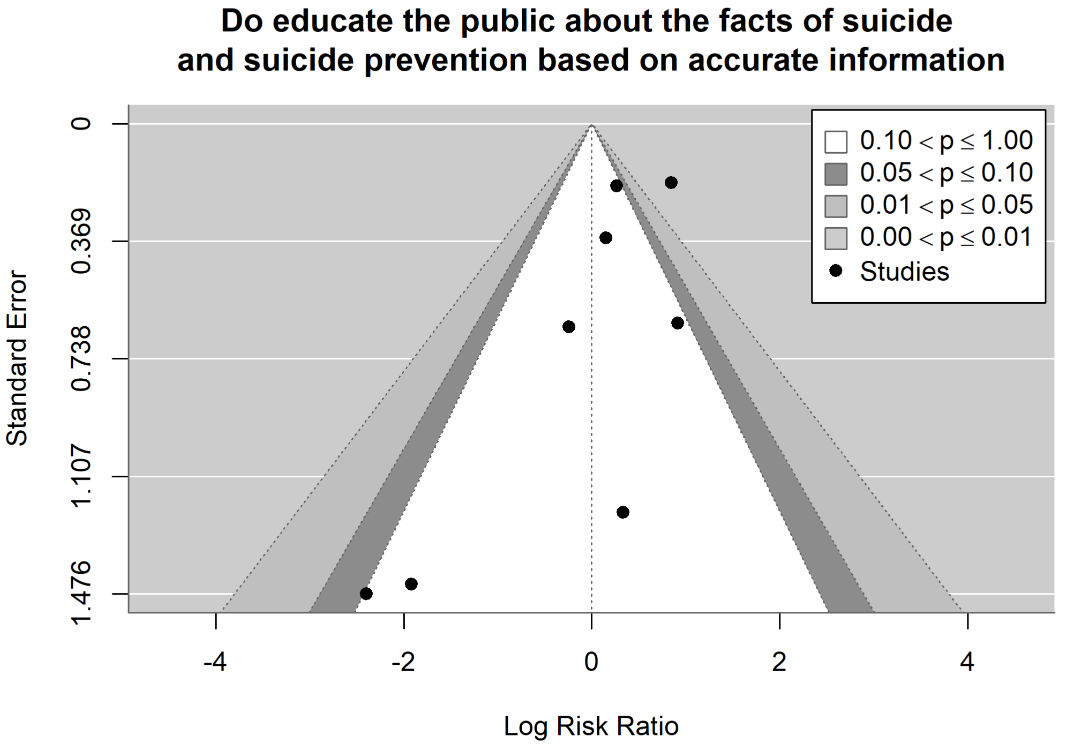

Do report stories of how to cope with life stressors and/or suicidal thoughts and the importance of help-seeking

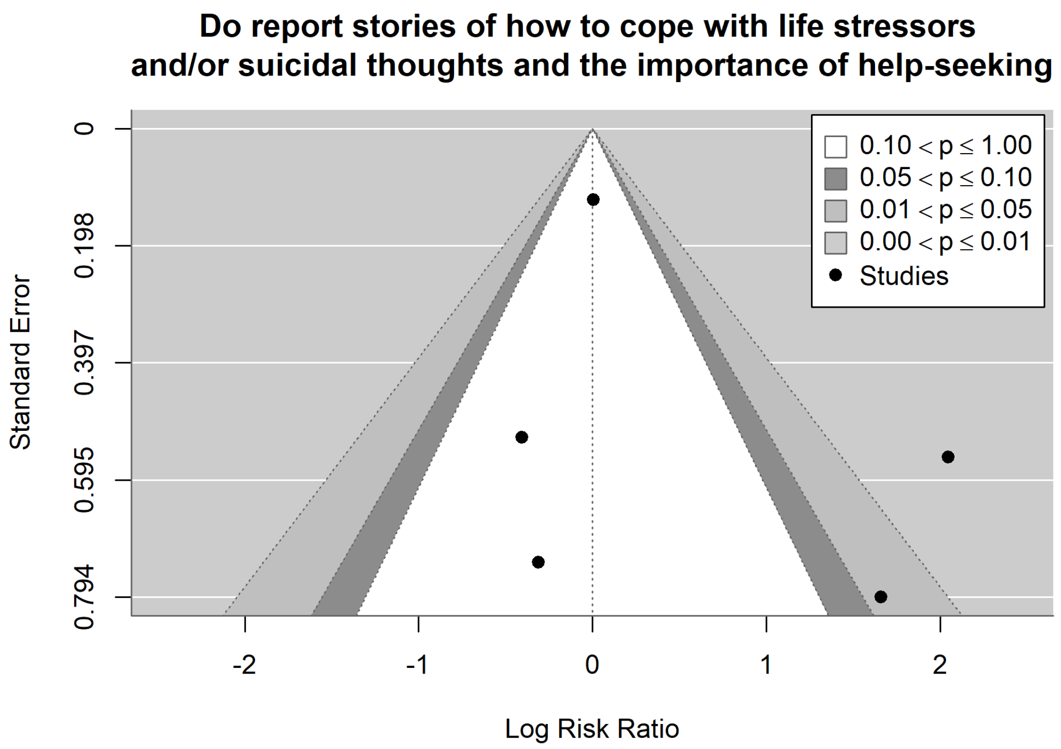

Do apply particular caution when reporting celebrity suicides

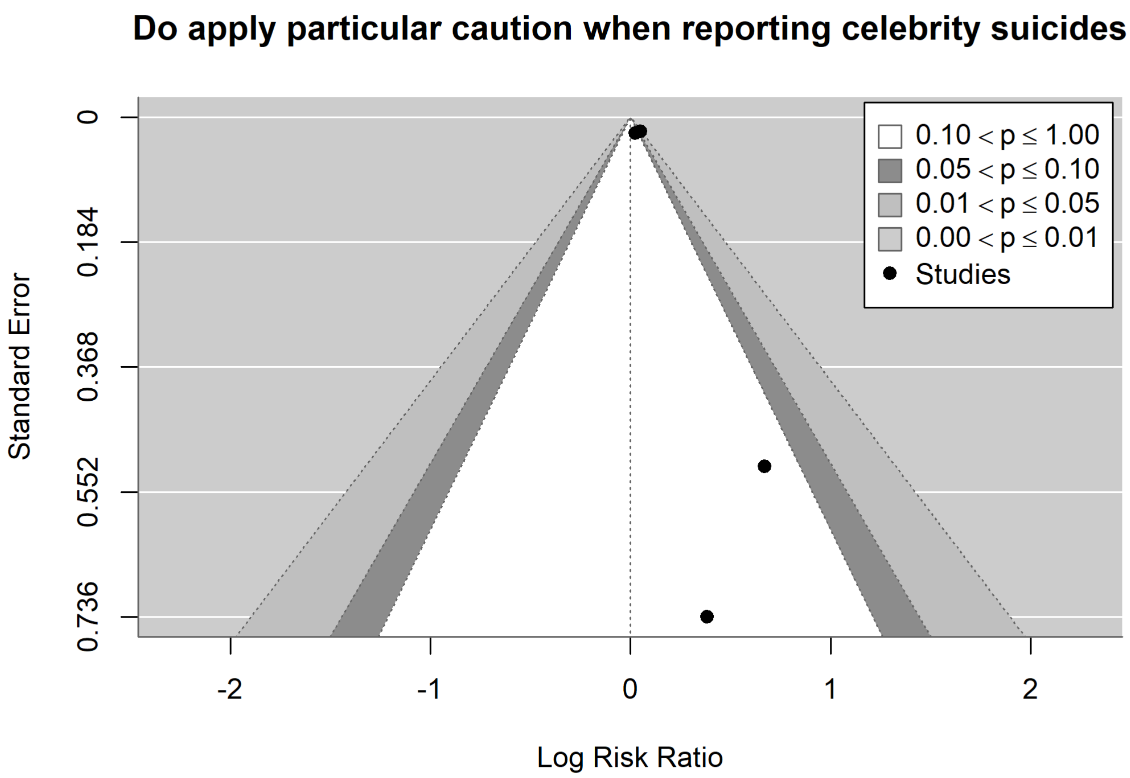

Do apply caution when interviewing bereaved family or friends or persons with lived experience

No studies with no gap between guideline release and the start of the post-intervention assessment period assessed this recommendation.

Don't position suicide-related content as the top story and don't unduly repeat such stories

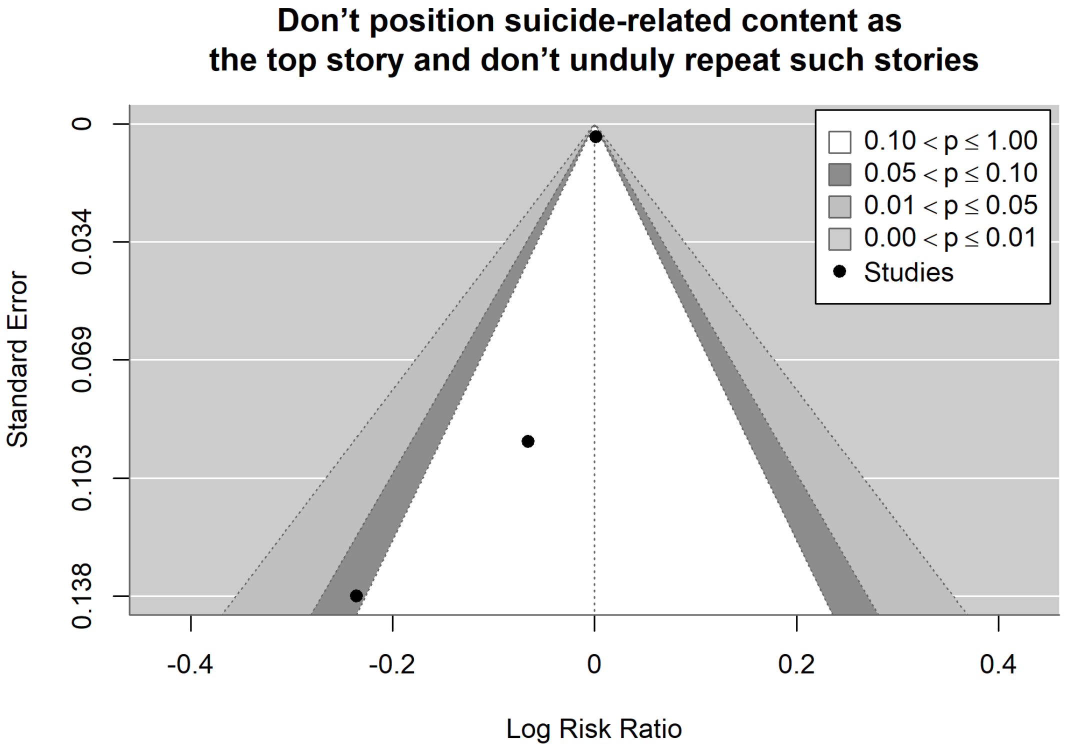

Don't describe the method used

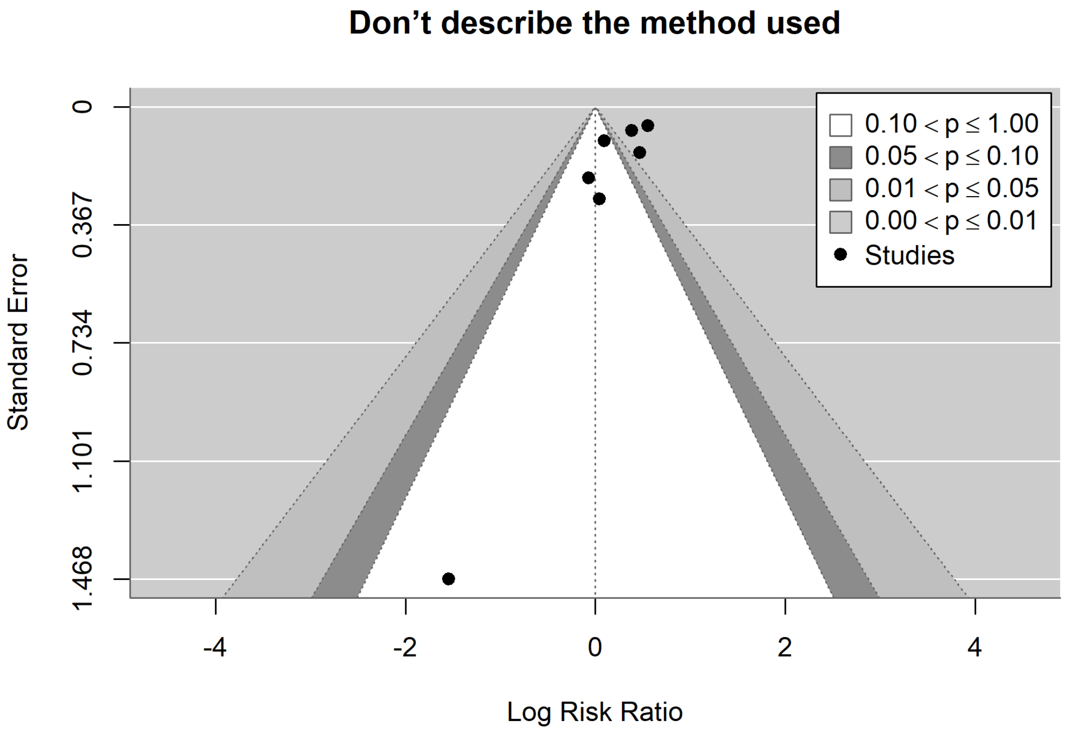

Don't name or provide details about the site/location

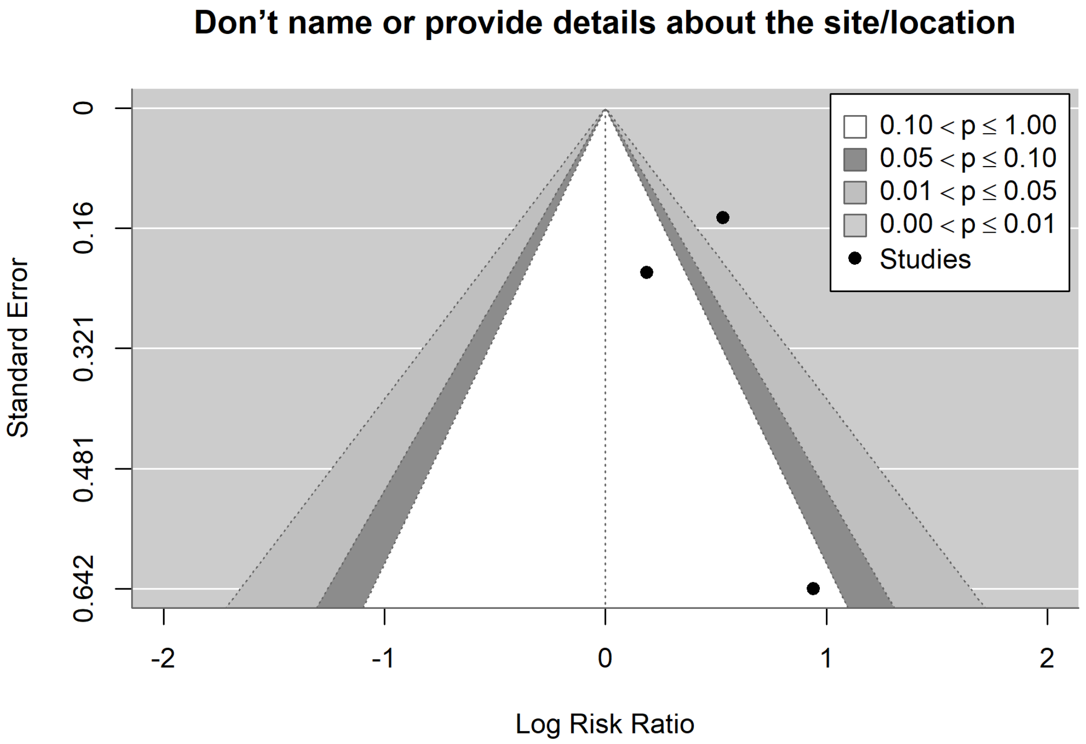

Don't use language/content which sensationalizes, romanticizes or normalizes suicide, or that presents it as a viable solution to problems

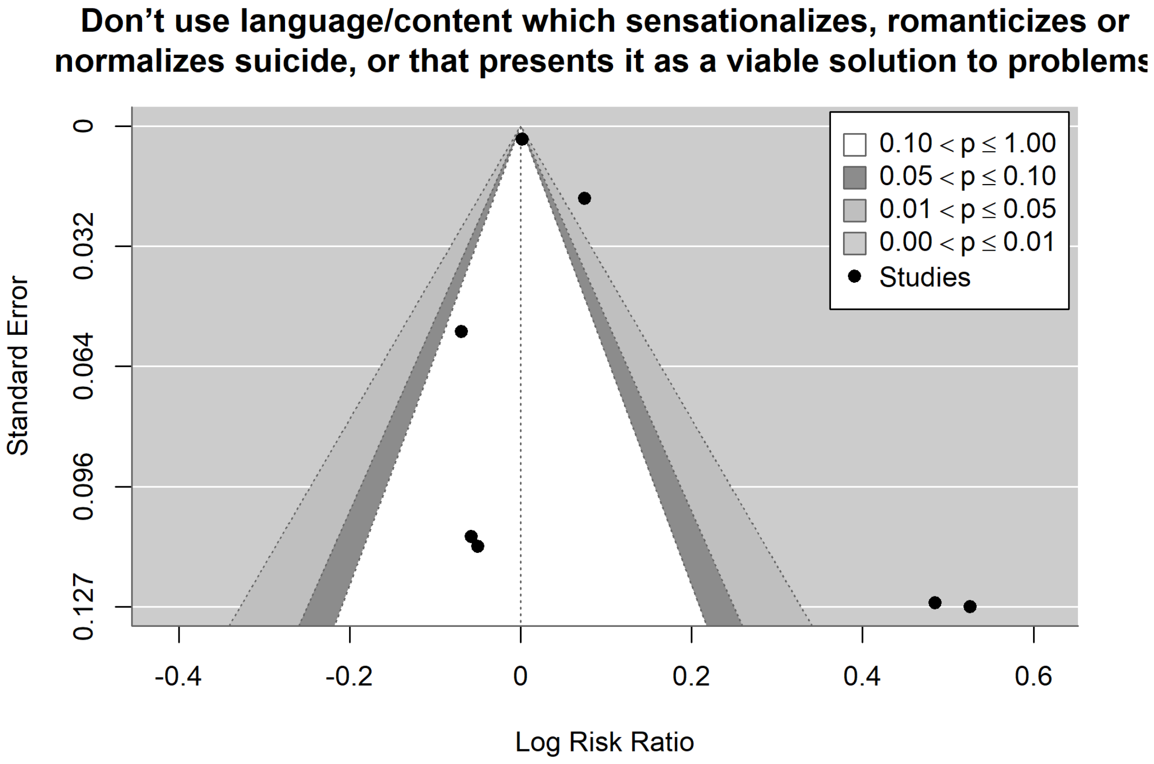

Don't oversimplify the reason for a suicide or reduce it to a single factor

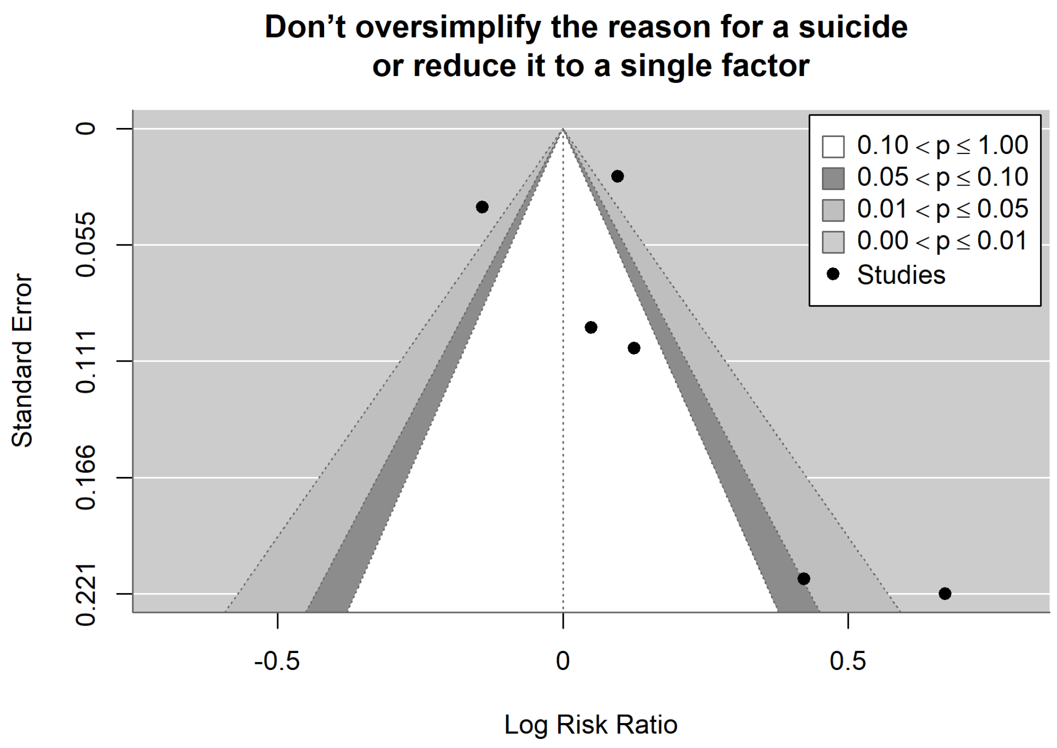

Don't use sensational language in headlines

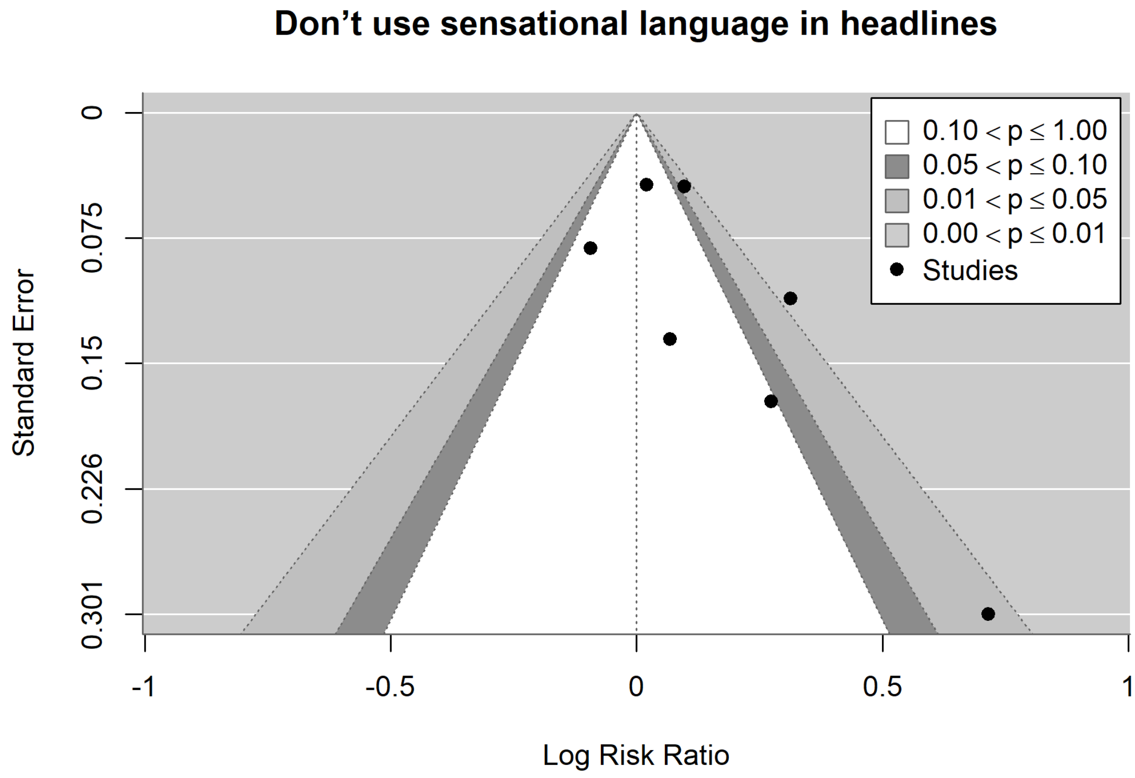

Don't use photographs, video footage, audio recordings, digital or social media links

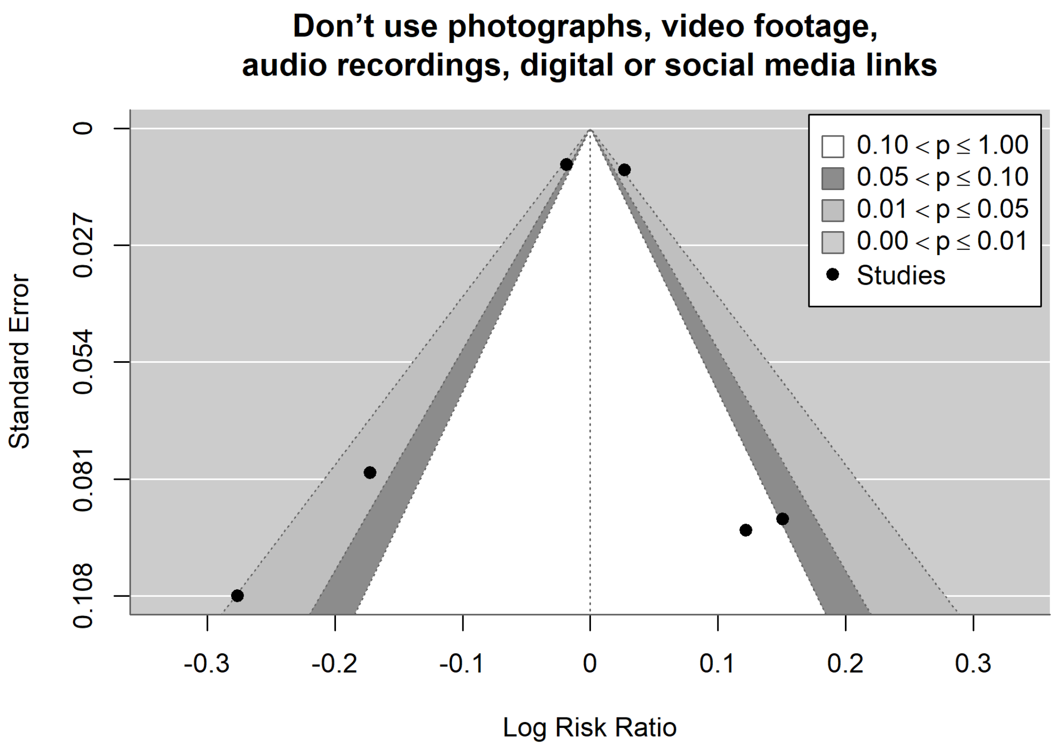

Don't report the details of a suicide note

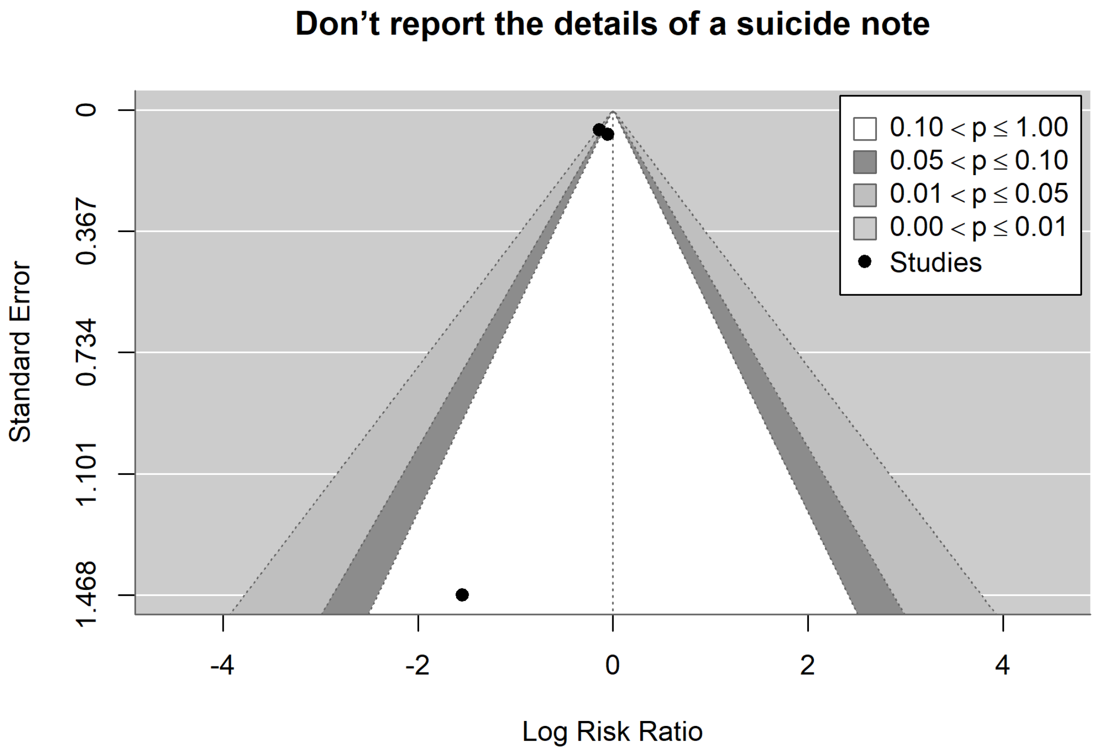

Supplement: Supplementary file 1 [file cri_47_3_188_esm1.pdf]
